# Resurrecting the Regulatory Properties of the *Ostreococcus tauri* ADP-Glucose Pyrophosphorylase Large Subunit

**DOI:** 10.3389/fpls.2018.01564

**Published:** 2018-10-30

**Authors:** Carlos M. Figueroa, Misty L. Kuhn, Benjamin L. Hill, Alberto A. Iglesias, Miguel A. Ballicora

**Affiliations:** ^1^Department of Chemistry and Biochemistry, Loyola University Chicago, Chicago, IL, United States; ^2^Instituto de Agrobiotecnología del Litoral, Universidad Nacional del Litoral, CONICET, Facultad de Bioquímica y Ciencias Biológicas, Santa Fe, Argentina

**Keywords:** 3-phosphoglycerate, ADP-glucose pyrophosphorylase, inorganic orthophosphate, *Ostreococcus tauri*, starch

## Abstract

ADP-glucose pyrophosphorylase (ADP-Glc PPase) catalyzes the first committed step for the synthesis of glycogen in cyanobacteria and starch in green algae and plants. The enzyme from cyanobacteria is homotetrameric (α_4_), while that from green algae and plants is heterotetrameric (α_2_β_2_). These ADP-Glc PPases are allosterically regulated by 3-phosphoglycerate (3PGA, activator) and inorganic orthophosphate (Pi, inhibitor). Previous studies on the cyanobacterial and plant enzymes showed that 3PGA binds to two highly conserved Lys residues located in the C-terminal domain. We observed that both Lys residues are present in the small (α) subunit of the *Ostreococcus tauri* enzyme; however, one of these Lys residues is replaced by Arg in the large (β) subunit. In this work, we obtained the K443R and R466K mutants of the *O. tauri* small and large subunits, respectively, and co-expressed them together or with their corresponding wild type counterparts. Our results show that restoring the Lys residue in the large subunit enhanced 3PGA affinity, whereas introduction of an Arg residue in the small subunit reduced 3PGA affinity of the heterotetramers. Inhibition kinetics also showed that heterotetramers containing the K443R small subunit mutant were less sensitive to Pi inhibition, but only minor changes were observed for those containing the R466K large subunit mutant, suggesting a leading role of the small subunit for Pi inhibition of the heterotetramer. We conclude that, during evolution, the ADP-Glc PPase large subunit from green algae and plants acquired mutations in its regulatory site. The rationale for this could have been to accommodate sensitivity to particular metabolic needs of the cell or tissue.

## Introduction

**FIGURE 1:**
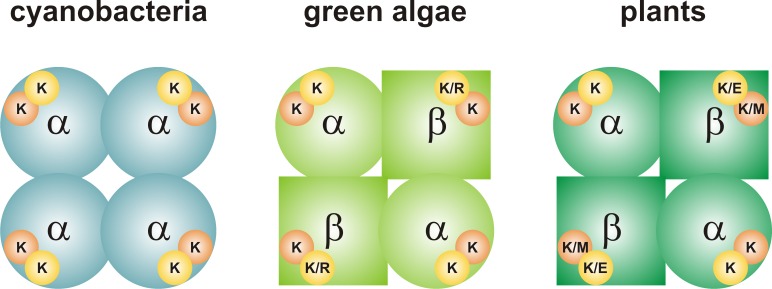
Quaternary structure of ADP-Glc PPases from oxygenic photosynthetic organisms. The enzyme from cyanobacteria (left) is a homotetramer (α_4_). Each subunit has a 3PGA-binding site, which contains two Lys residues. The enzymes from green algae and plants (center and right, respectively) are heterotetramers (α_2_β_2_). The 3PGA-binding site from the α subunit contains two Lys residues, while amino acid composition is less conserved in the β subunit. Orange circle, 3PGA-anchoring site 1; yellow circle, 3PGA-anchoring site 2. Further information on the composition of the 3PGA-anchoring sites can be found in Figures 2, 3.

ADP-glucose pyrophosphorylase (ADP-Glc PPase; EC 2.7.7.27) catalyzes the conversion of Glc1P and ATP into ADP-glucose and inorganic pyrophosphate (PPi), in the presence of a divalent cation (Mg^2+^). This is the first committed step for glycogen biosynthesis in bacteria and starch biosynthesis in green algae and plants. The native, catalytically active enzyme from cyanobacteria is a homotetramer (α_4_), whereas that from green algae and higher plants is a heterotetramer (α_2_β_2_) (Figure 1; Ballicora et al., 2003, 2004). The sequence identity between α subunits (also called small, S) from different species is higher than that of β subunits (also called large, L), and it has been proposed that L subunits differentially emerged through a process of gene duplication followed by subfunctionalization (Ballicora et al., 2005; Georgelis et al., 2008; Kuhn et al., 2009). In this proposed process, some S subunits retained a particular role, while the counterpart L subunits kept a different one. Originally, the S subunit from potato tuber (*Stu*S) was described as mostly catalytic, whereas the L subunit modulated the regulation of the S subunit (Ballicora et al., 1995); however, different alternatives were also observed. For instance, the L subunit can also be catalytically active in some organisms or in different isoforms within the same organism (Ventriglia et al., 2008; Kuhn et al., 2009).

While catalytic properties of the S and L subunits vary across species, their roles in regulation and sensitivity to effectors can also differ. For example, S subunits from potato tuber and *Arabidopsis thaliana* (APS1) ADP-Glc PPases show a low apparent affinity for (3PGA, activator) when expressed as homotetramers (Ballicora et al., 1995; Crevillen et al., 2003). When the S subunit is combined with the L subunit, the apparent affinity for 3PGA significantly increases. In the case of *A. thaliana*, there is only one functional gene encoding the S subunit (*APS1*), while there are four genes that encode L subunits (*APL1-4*) (Crevillen et al., 2003). *APS1* is expressed in all tissues but the expression pattern and response to sugars for *APL1-4* differs according to the tissue (Crevillen et al., 2005). The combination of APS1 with different L subunits renders heterotetramers with distinct kinetic and regulatory properties (Crevillen et al., 2003).

A similar subfunctionalization scenario was reported for ADP-Glc PPase from the green alga *Ostreococcus tauri*, which is at the base of the green linage (Derelle et al., 2006). This species has only one gene encoding each subunit (S and L), which makes it a very suitable model system to study the evolution of structure to function relationships of this enzyme. In this particular case, the S subunit (*Ota*S) displays the typical low apparent affinity for the activator (3PGA) and is defective for catalysis, whereas the L subunit (*Ota*L) shows higher affinity for substrates, suggesting a leading role in catalysis (Kuhn et al., 2009). Moreover, *Ota*L could be considered an activator “specifier,” as it specifically increases the apparent affinity for 3PGA of *Ota*S (Kuhn et al., 2013).

**FIGURE 2:**
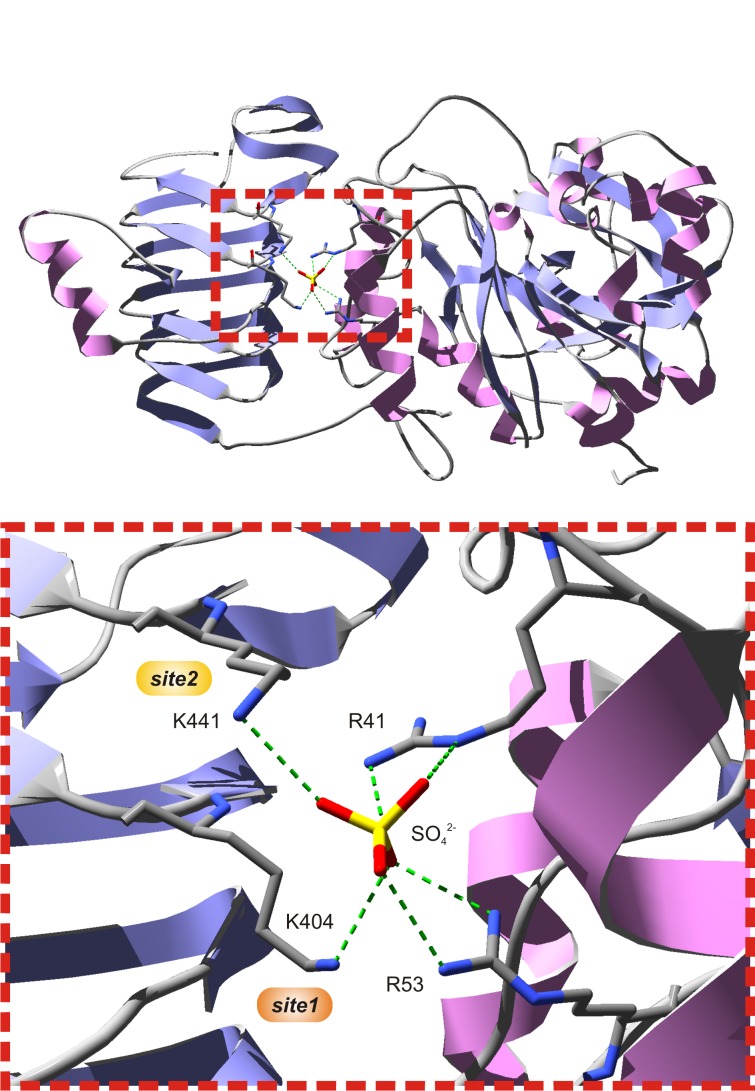
The putative 3PGA-binding site of the potato tuber S subunit. The top figure shows subunit A from the potato tuber ADP-Glc PPase S subunit homotetrameric structure (PDB ID 1YP4). The bottom figure is a zoom into the red, dotted box from the top figure. A sulfate ion from crystallization occupies the position where the phosphate moiety from 3PGA is hypothesized to be located, at the interface between the C- and N-terminal domains (left and right, respectively) of each subunit. This sulfate establishes H-bonds with Arg^41^ and Arg^53^ from the N-terminal domain, and Lys^404^ and Lys^441^ from the C-terminal domain. Lys^404^ (3PGA-anchoring site 1, orange) and Lys^441^ (3PGA-anchoring site 2, yellow) have been implicated in 3PGA binding by chemical modification and site-directed mutagenesis studies. Secondary structures and atoms were colored as follows: α-helices, pink; β-sheets, violet; loops, light gray; carbon, dark gray; nitrogen, blue; oxygen, red; sulfate, yellow. H-bonds are shown in green dashes.

Several studies have shown that Lys residues located at the C-terminal domain of cyanobacterial (Charng et al., 1994; Sheng et al., 1996) and plant (Morell et al., 1988; Ball and Preiss, 1994; Ballicora et al., 1998) ADP-Glc PPases play an important role for 3PGA binding. The three-dimensional structure of the potato tuber S subunit homotetramer (α_4_) showed that Lys^404^ and Lys^441^ make hydrogen bonds with a sulfate ion present in the crystal (Figure 2), where it is likely that the phosphate moiety of 3PGA also binds (Jin et al., 2005). Lys^404^ (from now on, 3PGA-anchoring site 1) is highly conserved in enzymes from cyanobacteria and plants (in both S and L subunits). Conversely, Lys^441^ (from now on, 3PGA-anchoring site 2) is usually present in cyanobacterial ADP-Glc PPases and the S subunit from plant enzymes, but is less conserved in plant L subunits (Ballicora et al., 1998; Jin et al., 2005). Our sequence analysis showed that both 3PGA-anchoring sites are present in *Ota*S (Lys^406^ and Lys^443^). However, only one Lys residue is found in *Ota*L (Lys^429^, 3PGA-anchoring site 1), while the second Lys is replaced by an Arg residue (Arg^466^, 3PGA-anchoring site 2).

Since the roles of the S and L subunits in green algae present unique characteristics, we wanted to explore how changes to the 3PGA-anchoring site affect the regulatory properties of the subunits. To the best of our knowledge, these residues have not been experimentally studied in green algae. Therefore, we further explored the importance of the 3PGA-anchoring site 2 in the *Ota*S/*Ota*L heterotetramer by co-expressing the wild-type *Ota*S or *Ota*L subunits with their corresponding mutant counterparts (*Ota*L_R466K_ or *Ota*S_K443R_) and analyzed their kinetic parameters. To better assess the individual contribution of *Ota*L to 3PGA activation, we also co-expressed *Ota*L and *Ota*L_R466K_ with the *Stu*S_K441A_ mutant (Ballicora et al., 1998), which mimics the mutation introduced in *Ota*S. Our experiments showed that introduction of a Lys residue in the 3PGA-anchoring site 2 of *Ota*L (to replace the native Arg residue) improved 3PGA activation. These results are discussed in the context of protein evolution and subunit subfunctionalization.

## Materials and Methods

### Chemicals

Substrates and effectors used for enzymatic assays were from Sigma-Aldrich (St. Louis, MO, United States). α-D-[^14^C(U)]-Glc1P was purchased from PerkinElmer (Waltham, MA, United States). All other reagents were of the highest quality available.

### Site-Directed Mutagenesis

Point mutations were introduced by PCR. Mutant sequences were amplified using pMAB5/*Ota*S and pMAB6/*Ota*L (Kuhn et al., 2009) as templates and the reverse primers *Ota*S_K443R_ (5′-GAGCTCTTAGATCACAGTGCCAGGTTTGATTACCGCATCgcgG ATGAC-3′) and *Ota*L_R466K_ (5′-GAGCTCTTAAATGATCGTACCATCTGGGATCGTGCAGTTtttCAGGAT-3′), respectively. Codons used to introduce point mutations are in lower-case letters and the *Sac*I restriction sites are underlined. Forward primers were the same used by Kuhn et al. (2009) to obtain the wild type sequences. Primers were synthesized by Integrated DNA Technologies (San Diego, CA, United States). The plasmid containing the mutant *Stu*S_K441A_ was obtained from Ballicora et al. (1998).

### Expression and Purification

Recombinant proteins were expressed in *Escherichia coli* AC70R1-504, which is deficient in ADP-Glc PPase activity (Iglesias et al., 1993). Enzyme purification was performed as described by Kuhn et al. (2013). Briefly, crude extracts were loaded on a 10 ml DEAE-Sepharose column (GE Healthcare, Piscataway, NJ, United States) and proteins were eluted with a linear NaCl gradient. Fractions containing ADP-Glc PPase activity were pooled and loaded onto two 1 ml Resource PHE columns (GE Healthcare) connected in tandem. Proteins were eluted with a linear ammonium sulfate gradient and fractions containing ADP-Glc PPase activity were pooled. Enzymes were concentrated using Amicon Ultra-15 centrifugal filter units (Millipore, Billerica, MA, United States) and stored at −80°C until use. Purity of all enzyme preparations used in this work was greater than 90%, as assessed by SDS–PAGE followed by densitometry.

### Activity Assays and Kinetic Analysis

Enzyme activity was determined by measuring the production of ADP-[^14^C]Glc from [^14^C]Glc1P, as described by Yep et al. (2004). Unless otherwise stated, the standard reaction mixture contained 50 mM HEPPS pH 8.0, 10 mM MgCl_2_, 1 mM [^14^C]Glc1P, 2.5 mM ATP, 0.2 mg/ml BSA, 0.75 U/ml inorganic pyrophosphatase, enzyme in a proper dilution, and varying amounts of activator/inhibitor. When activity of the hybrid heterotetramers (containing the *Stu*S_K441A_ subunit) was assayed, 2 mM DTT was added to the reaction media. Alternatively, enzyme activity was assayed by using a colorimetric method. In this case, radioactive Glc1P was replaced by unlabeled Glc1P and Pi (resulting from the hydrolysis of PPi by inorganic pyrophosphatase) was determined as previously described (Fusari et al., 2006). One unit of enzyme activity is defined as the amount of enzyme producing 1 μmol of ADP-[^14^C]Glc (with the radioactive method) or PPi (with the colorimetric method) in 1 min at 37°C.

Kinetic parameters were determined by plotting activity data against the variable concentration of substrate or effector using the program Origin 7.0 (OriginLab Corporation). Data were fitted to a modified Hill equation: *v* = *v*_0_+(*V*−*v*_0_)^∗^C*^n^*^H^/(*k^n^*^H^+C*^n^*^H^), where *v* is the initial velocity; *v*_0_ is the velocity in absence of the substrate or effector being analyzed; *V* is the maximal velocity (*V*_max_), activation or inhibition; C is the concentration of substrate or effector under study; *k* is the concentration of substrate or effector producing half of the maximal velocity (*S*_0.5_), activation (*A*_0.5_), or inhibition (*I*_0.5_), respectively, and *n*_H_ is the Hill coefficient. Standard deviations were calculated by the fitting software. Kinetic experiments were performed at least twice with similar results. The net activation fold (i.e., the activation that is over one) was calculated as (*V*_max_−*v*_0_)/*v*_0_ (Kuhn et al., 2013).

### Sequence Analysis

Protein sequences of ADP-Glc PPases from cyanobacteria, green algae, and plants were downloaded from the NCBI database^1^. Duplicated and incomplete sequences were discarded. The N-terminal sequence of enzymes from green algae and plants, including a chloroplast transit peptide, were manually trimmed. Sequences were aligned using the ClustalW multiple sequence alignment server^2^ (Jeanmougin et al., 1998). The resulting alignment was manually refined with BioEdit 7.0^3^ (Hall, 1999) based on structural data from the potato tuber ADP-Glc PPase (Jin et al., 2005). The 3PGA-anchoring sites 1 and 2 were analyzed using the WebLogo server^4^ (Schneider and Stephens, 1990; Crooks et al., 2004).

## Results

**FIGURE 3:**
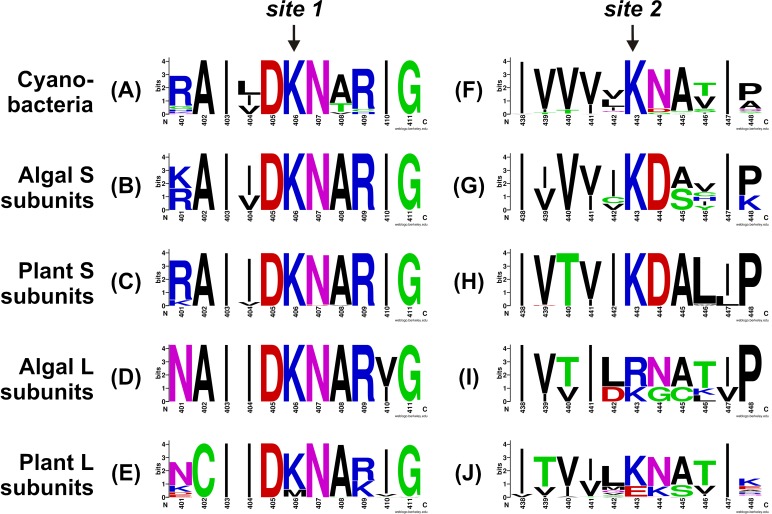
Sequence logos for the C-terminal 3PGA-anchoring site sequences of ADP-Glc PPases from oxygenic photosynthetic organisms. Aligned sequences were submitted to the WebLogo server and plots were generated for 3PGA-anchoring sites 1 **(A–E)** and 2 **(F–J)** from cyanobacteria **(A,F)**, algal S subunits **(B,G)**, plant S subunits **(C,H)**, algal L subunits **(D,I)**, and plant L subunits **(E,J)**. Sequence logos were prepared using the sequences from Supplementary Table S1 and Supplementary Figure S1. Numbers below the residues correspond to the *Ota*S sequence.

To better understand the conservation of the 3PGA binding site in ADP-Glc PPases from oxygenic photosynthetic organisms, we built sequence logos for the two C-terminal 3PGA-anchoring sites (Lys residues in Figure 2) using sequences from cyanobacteria, algae, and plants (Figure 3). Figures 1, 3A–D show that the Lys residue located at the 3PGA-anchoring site 1 is highly conserved in enzymes from cyanobacteria, the S subunits from algae and plants, and the L subunits from algae. However, there are some L subunits from plants where the Lys residue has been replaced by a Met (Figures 1, 3E). Figures 3A–E also shows that the Lys residue from 3PGA-anchoring site 1 is flanked by Asp and Asn, constituting a very well conserved domain. The scenario is less clear for the 3PGA-anchoring site 2 (Figures 3F–J). This Lys residue is present in all sequences from cyanobacteria and S subunits from algae and plants (Figures 1, 3F–H), but it is replaced (although not always) by Arg and Glu in L subunits from algae and plants, respectively (Figures 1, 3I,J). After a careful analysis of all sequences (Supplementary Figure S1 and Supplementary Table S1), some patterns could be distinguished. In the homotetrameric enzyme from cyanobacteria the Lys residue is usually followed by Asn (Figure 3F), while in S subunits from algae and plants the Lys residue is always followed by Asp (Figures 3G,H). In L subunits from algae and plants there are two main combinations: Arg-Asn/Lys-Gly and Lys-Asn/Glu-Lys, respectively (Figures 3I,J).

As mentioned above, *Ota*L has an Arg instead of a Lys in the 3PGA-anchoring site 2. To further explore the role of this residue in the S and L subunits of the *O. tauri* heterotetramer, we performed site-directed mutagenesis to obtain individual *Ota*S_K443R_ and *Ota*L_R466K_ mutant subunits. These mutants were co-expressed with each other or the wild type subunits to obtain the corresponding heterotetramers, i.e., *Ota*S/*Ota*L_R466K_, *Ota*S_K443R_/OtaL, and *Ota*S_K443R_/*Ota*L_R466K_. Table 1 shows the kinetic parameters for Glc1P and ATP in absence or presence of 3PGA for the wild type and mutant enzymes. In absence of 3PGA, *S*_0.5_ values for both substrates were similar for all enzymes. Addition of 5 mM 3PGA significantly reduced the *S*_0.5_ for Glc1P in *Ota*S/*Ota*L and *Ota*S/*Ota*L_R466K_ (25- and 17-fold, respectively; Table 1). The *S*_0.5_ for Glc1P of *Ota*S_K443R_/OtaL and *Ota*S_K443R_/*Ota*L_R466K_ also decreased in presence of 3PGA, but only fourfold and sevenfold, respectively (Table 1). The activator had a less pronounced effect on the *S*_0.5_ for ATP, which was reduced by twofold for *Ota*S/*Ota*L and *Ota*S_K443R_/*Ota*L and by fivefold for *Ota*S/*Ota*L_R466K_ and *Ota*S_K443R_/*Ota*L_R466K_ (Table 1).

**Table 1:** Substrate kinetic parameters.

		Glc1P		ATP	
	3PGA				
Enzyme	(mM)	*S*_0.5_ (mM)	*n*_H_	*S*_0.5_ (mM)	*n*_H_
*Ota*S/*Ota*L	0	2.2 ± 0.4	1.0	0.87 ± 0.05	1.1
	5	0.086 ± 0.002	1.5	0.37 ± 0.01	1.5
*Ota*S/*Ota*L_R466K_	0	1.8 ± 0.4	1.0	1.28 ± 0.07	1.1
	5	0.103 ± 0.005	1.5	0.25 ± 0.01	1.4
*Ota*S_K443R_/*Ota*L	0	1.5 ± 0.9	0.9	0.9 ± 0.2	1.6
	5	0.37 ± 0.04	0.9	0.58 ± 0.07	1.9
*Ota*S_K443R_/*Ota*L_R466K_	0	1.9 ± 0.5	0.8	1.7 ± 0.7	0.9
	5	0.28 ± 0.01	1.7	0.35 ± 0.05	1.3

*Saturation curves for Glc1P and ATP were performed in absence or presence of 5 mM 3PGA. Enzyme activity was assayed using the colorimetric method, as described in Section “Materials and Methods.”*

**FIGURE 4:**
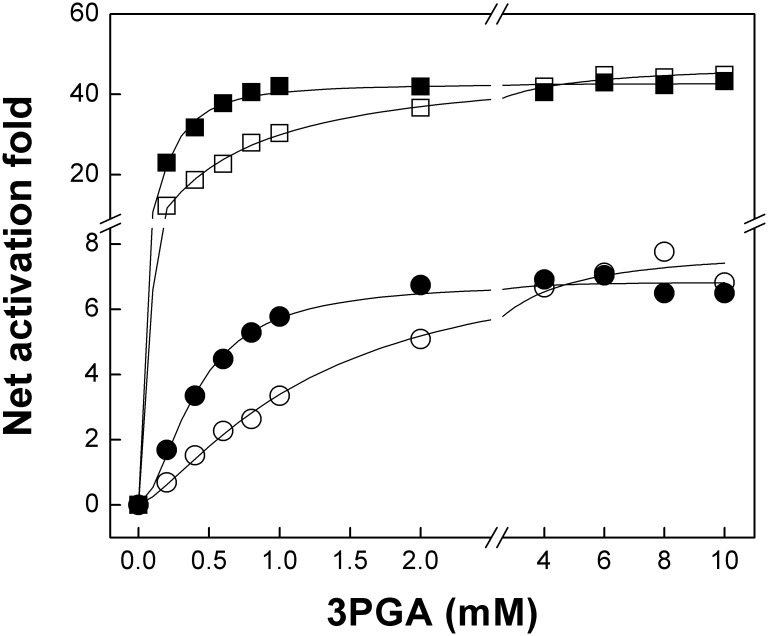
The *Ota*L_R466K_ mutant has increased sensitivity to 3PGA. The figure shows 3PGA curves for *Ota*S/*Ota*L (white squares), *Ota*S/*Ota*L_R466K_ (black squares), *Stu*S_K441A_/*Ota*L (white circles), and *Stu*S_K441A_/*Ota*L_R466K_ (black circles). Enzyme activity was assayed using the radioactive method, as described in Section “Materials and Methods.”

We then analyzed the activation of the mutant heterotetramers by 3PGA in more detail. Mutations introduced in both *Ota*S and *Ota*L reduced *v*_0_ by threefold and affected *V*_max_ to a different extent; thus, we used the net activation fold to compare mutants with their wild type counterparts (Table 2). The *A*_0.5_ for 3PGA was threefold lower in *Ota*S/*Ota*L_R466K_ than in *Ota*S/*Ota*L, but no significant changes were observed in the net activation fold (Table 2 and Figure 4). The *A*_0.5_ reduced twofold and the net activation fold increased threefold for *Ota*S_K443R_/*Ota*L_R466K_ compared to *Ota*S_K443R_/*Ota*L (Table 2). Similar results were obtained when we co-expressed the mutant *Stu*S_K441A_ (analogous to *Ota*S_K443R_) with *Ota*L or *Ota*L_R466K_. The *A*_0.5_ for 3PGA decreased threefold for *Stu*S_K441A_/*Ota*L_R466K_ compared to *Stu*S_K441A_/*Ota*L, while the net activation fold remained the same (Table 2 and Figure 4). Opposite changes were observed when we analyzed the heterotetramers containing the *Ota*S_K443R_ mutant with those containing the wild type S subunit. Table 2 shows that the *A*_0.5_ for 3PGA increased twofold for *Ota*S_K443R_/*Ota*L (compared to *Ota*S/*Ota*L) and fourfold for *Ota*S_K443R_/*Ota*L_R466K_ (compared to *Ota*S/*Ota*L_R466K_). The net activation fold decreased 14-fold and fourfold, respectively.

**Table 2:** Activation kinetic parameters.

Enzyme	Pi (mM)	*A*_0.5_ (mM)	*n*_H_	*v*_0_ (U mg^−1^)	*V*_max_ (U mg^−1^)	Net activation fold
*Ota*S/*Ota*L	0	0.61 ± 0.03	1.0	0.89	43.5 ± 0.8	48
	1	4.6 ± 0.2	2.1	0.60	36 ± 2	59
*Ota*S/*Ota*L_R466K_	0	0.19 ± 0.01	1.7	0.29	12.6 ± 0.2	42
	1	1.8 ± 0.1	2.2	0.34	11.9 ± 0.3	34
*Ota*S_K443R_*/Ota*L	0	1.6 ± 0.6	0.8	0.31	1.4 ± 0.1	3.5
	1	2.0 ± 0.1	1.6	0.34	1.04 ± 0.02	2.1
*Ota*S_K443R_*/Ota*L_R466K_	0	0.74 ± 0.08	1.7	0.38	4.1 ± 0.2	9.8
	1	2.2 ± 0.2	2.3	0.27	2.50 ± 0.09	8.3
*Stu*S_K441A_/*Ota*L	0	1.2 ± 0.1	1.4	1.54	13.6 ± 0.6	7.8
	1	4.4 ± 0.4	2.3	0.45	12.8 ± 0.8	27
*Stu*S_K441A_/*Ota*L_R466K_	0	0.40 ± 0.02	1.8	1.72	13.5 ± 0.2	6.8
	1	3.1 ± 0.1	2.5	0.36	14.1 ± 0.4	38

*Saturation curves for 3PGA were performed in absence or presence of 1 mM Pi. Enzyme activity was assayed using the radioactive method, as described in Section “Materials and Methods.”*

It has been shown that 3PGA activation and Pi inhibition are structurally interconnected (Ball and Preiss, 1994; Ballicora et al., 1998; Jin et al., 2005). Because the mutants analyzed in this work showed altered 3PGA activation, it was of interest to test whether Pi inhibition was also changed. Thus, we also performed 3PGA activation curves with the addition of 1 mM Pi. In all cases, the *A*_0.5_ for 3PGA increased in presence of the inhibitor; however, changes were more pronounced for heterotetramers containing the *Ota*L_R466K_ mutant compared to those with the *Ota*L subunit (Table 2). Namely, the *A*_0.5_ for *Ota*S/*Ota*L_R466K_, *Ota*_SK443R_/*OtaL* and *Stu*S_K441A_/*Ota*L_R466K_ increased 10-, 3-, and 8-fold, respectively; while the *A*_0.5_ for *Ota*S/*Ota*L, *Ota*S_K443R_/*OtaL* and *Stu*S_K441A_/*Ota*L increased 7-, 1.3-, and 4-fold, respectively. Finally, we analyzed Pi inhibition in presence of 3PGA (using a concentration equal to the *A*_0.5_ for 3PGA of each heterotetramer, see Table 2 for concentrations). We observed that *I*_0.5_ values for *Ota*S/*Ota*L and *Ota*S/*Ota*L_R466K_ were similar; however, the *I*_0.5_ for Pi increased ninefold in *Ota*S_K443R_/*Ota*L (with respect to *Ota*S/*Ota*L) and fivefold in *Ota*S_K443R_/*Ota*L_R466K_ (compared to *Ota*S_K443R_/*Ota*L; Table 3).

**Table 3:** Inhibition kinetic parameters.

Enzyme	*I*_0.5_ (mM)	*n*_H_
*Ota*S/*Ota*L	0.179 ± 0.004	1.9
*Ota*S/*Ota*L_R466K_	0.139 ± 0.004	1.6
*Ota*S_K443R_/*Ota*L	1.6 ± 0.2	1.7
*Ota*S_K443R_/*Ota*L_R466K_	0.75 ± 0.08	1.4

*Saturation curves for Pi were performed at the A_0.5_ for 3PGA of each enzyme (see Table 2). Enzyme activity was assayed using the radioactive method, as described in Section “Materials and Methods.”*

## Discussion

Early chemical modification studies performed with pyridoxal-phosphate (a structural analog of 3PGA) on the ADP-Glc PPase from spinach leaf demonstrated that 3PGA binds to Lys residues within the C-terminal domain of both S and L subunits (Morell et al., 1988; Ball and Preiss, 1994). Similar results were obtained with the enzyme from *Anabaena* sp. PCC 7120, where Lys^382^ and Lys^419^ were modified by pyridoxal-phosphate (Charng et al., 1994). These results were confirmed by site-directed mutagenesis of the *Anabaena* ADP-Glc PPase (Charng et al., 1994; Sheng et al., 1996) and both subunits of the potato tuber enzyme (Ballicora et al., 1998). Mutation of Lys^404^ and Lys^441^ in *Stu*S had larger effects on 3PGA activation than mutation of the corresponding residues in the L subunit (Lys^417^ and Lys^455^, respectively). Random-mutagenesis studies performed with the L subunit of the potato tuber enzyme provided evidence that Asp^416^ is important for 3PGA binding (Greene et al., 1996). This acidic residue precedes Lys^417^ in the 3PGA-anchoring site 1 (Figures 3A–E). The Lys residue located in this site is surrounded by highly conserved amino acids (Asp and Asn), which agrees with the higher importance of the 3PGA-anchoring site 1 for activation (Ballicora et al., 1998). Interestingly, some L subunits from grass species have a Met replacing the Lys residue from 3PGA-anchoring site 1 (Supplementary Table S1 and Supplementary Figure S1). We speculate that such a change could be responsible for the low 3PGA sensitivity displayed by ADP-Glc PPases purified from barley (Kleczkowski et al., 1993) and wheat (Gomez-Casati and Iglesias, 2002) endosperm. The recombinant enzyme from maize endosperm shows a very particular response to 3PGA, which slightly increases the *V*_max_ but significantly reduces the *S*_0.5_ for both substrates, ATP and Glc1P (Boehlein et al., 2010, 2013). Further experiments would contribute to better understand the role of these residues for 3PGA activation in ADP-Glc PPases from grasses.

As shown in Figures 3F–J, the 3PGA-anchoring site 2 is less conserved; however, we found some patterns for residues located at this site. All S subunits from green algae and plants analyzed in this work have a Lys followed by an Asp residue (an opposite distribution compared to the 3PGA-anchoring site 1, Figures 3G,H). However, most enzymes from cyanobacteria and some L subunits from plants have a Lys followed by an Asn residue, while other L subunits from plants (including the potato tuber L subunit) have a Glu followed by a Lys (Figures 3F,J). Interestingly, L subunits from green algae show very different amino acid combinations: Arg-Asn and Lys-Gly (Figures 1, 3I), which could provide particular allosteric properties to these enzymes and should be investigated in the future.

Kuhn et al. (2009) suggested that the *Ota*L protein underwent a different subfunctionalization process than L subunits from plants, which allowed *Ota*L to maintain a high affinity for both substrates (Glc1P and ATP) and the activator (3PGA). However, the activation of the *Ota*S_D148A_/*Ota*L mutant (whose S subunit lacks catalytic activity) was only threefold (Kuhn et al., 2009). We hypothesized that the presence of Arg^466^ in the 3PGA-anchoring site 2 of *Ota*L could be responsible for the low activation fold displayed by this subunit. To test our hypothesis, we obtained the mutant *Ota*L_R466K_ and the opposite mutation for the S subunit (*Ota*S_K443R_) and co-expressed them with their corresponding wild type counterparts. In presence of 3PGA, only minor changes in substrate kinetic parameters were observed for the mutant heterotetramers (Table 1), as was described for other ADP-Glc PPases with residues of 3PGA-anchoring sites 1 and 2 mutated (Charng et al., 1994; Sheng et al., 1996; Ballicora et al., 1998). Particularly, the *S*_0.5_ for Glc1P increased threefold to fourfold in enzymes containing the *Ota*S_K443R_ subunit (Table 1). We also found some differences that could be related to the presence of a Lys residue at the 3PGA-anchoring site 2. For example, reduction of the *S*_0.5_ for ATP in presence of 3PGA was more pronounced in heterotetramers containing the *Ota*L_R466K_ mutant than in those with the *Ota*L subunit (Table 1). Similarly, the *S*_0.5_ for Glc1P was significantly reduced in heterotetramers containing *Ota*S compared to those with the *Ota*S_K443R_ mutant (Table 1). These results suggest that the 3PGA-anchoring site 2 in *Ota*L is important for increasing the apparent affinity of the heterotetramer for ATP, while the same site in *Ota*S plays a similar role but for Glc1P binding. Thus, cross-talk between the subunits seems to play a critical role in this enzyme. As it was shown before in potato tuber and *A. thaliana*, the presence of the L subunit altered the regulation of the enzyme (Crevillen et al., 2003; Ballicora et al., 2005; Ventriglia et al., 2008). In *Ota*L, the presence of the S subunit seems to be important for this role (Kuhn et al., 2009). An allosteric network that involves the subunit-subunit interface may be very important for the regulation. Indeed, it has been suggested that kinetic and regulatory properties of ADP-Glc PPases result from the synergistic interaction between S and L subunits (Hwang et al., 2005). Georgelis et al. (2009) also showed that certain areas of the L subunit that interact with the S subunit are important for the allosteric properties of the maize endosperm ADP-Glc PPase.

Activation kinetics performed with the *Ota*S/*Ota*L_R466K_ mutant showed that the *A*_0.5_ for 3PGA was reduced compared with the wild type (Table 2 and Figure 4). This result is in good agreement with previous reports showing that a Lys residue in the 3PGA-anchoring site 2 is important for activation (Morell et al., 1988; Ball and Preiss, 1994; Charng et al., 1994; Sheng et al., 1996; Ballicora et al., 1998). As expected, the *A*_0.5_ for 3PGA increased for the *Ota*S_K443R_/OtaL mutant compared with the wild type, confirming the importance of the Lys residue in the 3PGA-anchoring site 2 of the S subunit. In this case, we observed a significant drop in the net activation fold (from 48 to 3, Table 2); confirming that 3PGA binding to the S subunit plays an important role for activation of the heterotetramer. Interestingly, the double mutant *Ota*S_K443R_/*Ota*L_R466K_ showed a higher activation fold and a lower *A*_0.5_ for 3PGA than the *Ota*S_K443R_/*Ota*L heterotetramer, which shows that introduction of a Lys residue in the 3PGA-anchoring site 2 of *Ota*L improves 3PGA activation (Table 2). Results obtained with the hybrids *Stu*S_K441A_*/Ota*L and *Stu*S_K441A_/*Ota*L_R466K_ were similar to those observed for *Ota*S/*Ota*L and *Ota*S/*Ota*L_R466K_, respectively; i.e., the heterotetramers containing the *Ota*L_R466K_ mutant showed lower *A*_0.5_ for 3PGA but retained the activation fold of the *Ota*L heterotetramers (Table 2 and Figure 4).

We also performed 3PGA curves for these enzymes in presence of 1 mM Pi. This strategy has been used to analyze the interplay of the regulators and to test the importance of critical residues for binding of the effectors in the potato tuber enzyme (Ballicora et al., 1998). Addition of Pi to the assay mixture increases the *A*_0.5_ and *n*_H_ for 3PGA and *vice versa*; i.e., addition of 3PGA to the reaction increases the *I*_0.5_ and *n*_H_ for Pi, not only in the potato tuber ADP-Glc PPase but also in enzymes from other oxygenic photosynthetic organisms (Ballicora et al., 2004). Thus, we used this strategy to boost the effect of the mutations introduced in both *Ota*S and *Ota*L subunits. Overall, addition of the inhibitor to the assay mixture increased the *A*_0.5_ for 3PGA of all heterotetramers (including the hybrids containing the *Stu*S_K441A_ mutant), although changes were more pronounced for those containing the *Ota*L_R466K_ mutant (Table 2). This indicates that introduction of a Lys residue in the 3PGA-anchoring site 2 of *Ota*L makes the heterotetramer more sensitive to 3PGA and, consequently, more resilient to Pi. Inhibition kinetics for Pi showed similarly low *I*_0.5_ values for the heterotetramers containing the *Ota*S subunit, but higher values for those with the *Ota*S_K443R_ mutant (Table 3). Only a minimal difference (twofold) was observed when comparing the *I*_0.5_ for Pi of the heterotetramers containing the *Ota*L_R466K_ mutant (Table 3). These results suggest that the Lys residue located at the 3PGA-anchoring site 2 plays a key role for Pi inhibition in *Ota*S, but it is less relevant for inhibition of *Ota*L.

Overall, our results show that 3PGA activation of the *O. tauri* ADP-Glc PPase could be improved (lower *A*_0.5_ for 3PGA than the wild type) by restoring the Lys residue in the 3PGA-anchoring site 2 of the L subunit. Sequence conservation of the S subunit is higher compared to the L subunit, suggesting more relaxed evolutionary constraints for the latter (Georgelis et al., 2008). It seems that after the gene duplication that generated the S and L subunits, mutations were introduced in the 3PGA-anchoring site 2 of the L subunit from algal and plant ADP-Glc PPases without affecting the overall role of the heterotetramer. The overlap of roles in both subunits generates a redundancy that leads to a subfunctionalization process. In the case of *O. tauri*, the integrity of the allosteric site in the S subunit prevailed and allowed deleterious changes in the allosteric site of the L subunit to occur. In the context of evolution, these changes could have been important to accommodate the regulatory properties of the enzyme to the metabolic needs of each species/tissue (Crevillen et al., 2003; Kuhn et al., 2013). Tissue-specific evolutionary tuning of the regulatory properties has been already described in plants. For instance, we have observed that different *A. thaliana* tissues require different apparent affinities for the activator 3-PGA, ranging from ∼20 to ∼900 μM (Crevillen et al., 2003). This effect results from combining different L subunits with a common S subunit. There, evolution may have tweaked the affinity of the L subunits with the activator or the inter-subunit interactions between the S subunit and the different L subunits to yield different allosteric behaviors. Here, we show the regulatory site of the *Ota* L subunit enzyme was mutated and the affinity for the activator was consequently decreased. The subfunctionalization process and the overlapping of roles, which increased the landscape to explore new roles or the optimization of existing ones, may have facilitated this tuning.

## Author Contributions

MB and AI conceived the research. CF, MK, and BH performed the experiments. CF prepared the main text, figures, and tables. All authors analyzed the data and revised the manuscript.

## Conflict of Interest Statement

The authors declare that the research was conducted in the absence of any commercial or financial relationships that could be construed as a potential conflict of interest.

## Footnotes

1 https://www.ncbi.nlm.nih.gov/

2 http://www.genome.jp/tools/clustalw/

3 http://www.mbio.ncsu.edu/bioedit/bioedit.html

4 http://weblogo.berkeley.edu/

## Abbreviations

3PGA: 3-phosphoglycerate
ADP-Glc PPase: ADP-glucose pyrophosphorylase
Glc1P: glucose 1-phosphate
L: ADP-Glc PPase large subunit
*Ota*: *Ostreococcus tauri*
Pi: inorganic orthophosphate
S: ADP-Glc PPase small subunit
*Stu*: *Solanum tuberosum*
