# Supplementary material for: Resurrecting the Regulatory Properties of the Ostreococcus tauri ADP-Glucose Pyrophosphorylase Large Subunit
Source: Front Plant Sci. 2018 Oct 30;9:1564. doi: 10.3389/fpls.2018.01564 (PMC6218581; doi:10.3389/fpls.2018.01564)
Supplement: Supplementary file 1 [file Image_1.PDF]

Figure S1. Protein sequence alignment of ADP-Glc PPases from cyanobacteria, green algae, and plants. Numbers correspond to data presented in Table S1. Residues were colored based on their chemical properties. Sequences were obtained from Kuhn et al. (2013).

```
             10        20        30        40        50        60        70        80        90       100
     ....|....|....|....|....|....|....|....|....|....|....|....|....|....|....|....|....|....|....|....|
1    SRS--V--LGIILG--GGAGTRLYPLTKKRAKPAVPLGANYRL--IDIPVSNCLN--SNI--SKIYVLT--QFNS--ASLNRHLSRA--YASNMGGYKNE
2    SRS--V--LGIILG--GGAGTRLYPLTKKRAKPAVPLGANYRL--IDIPVSNCLN--SNI--SKIYVLT--QFNS--ASLNRHLSRA--YASNMGGYKNE
3    SRS--V--LGIILG--GGAGTRLYPLTKKRAKPAVPLGANYRL--IDIPVSNCLN--SNI--SKIYVLT--QFNS--ASLNRHLSRA--YASNMGGYKNE
4    SRS--V--LGIILG--GGAGTRLYPLTKKRAKPAVPLGANYRL--IDIPVSNCLN--SNI--SKIYVLT--QFNS--ASLNRHLSRA--YASNMGGYKNE
5    SSS--V--LGIILG--GGAGTRLYPLTKKRAKPAVPLGANYRL--IDIPVSNCLN--SNI--SKIYVLT--QFNS--ASLNRHLSRA--YASNMGGYKNE
6    SSS--V--LGIILG--GGAGTRLYPLTKKRAKPAVPLGANYRL--IDIPVSNCLN--SNI--SKIYVLT--QFNS--ASLNRHLSRA--YASNMGGYKNE
7    SRS--V--LGIILG--GGAGTRLYPLTKKRAKPAVPLGANYRL--IDIPVSNCLN--SNI--SKIYVLT--QFNS--ASLNRHLSRA--YASNLGGYKNE
8    SRS--V--LGIILG--GGAGTRLYPLTKKRAKPAVPLGANYRL--IDIPVSNCLN--SNI--SKIYVLT--QFNS--ASLNRHLSRA--YASNMGGYKNE
9    SRS--V--LGIILG--GGAGTRLYPLTKKRAKPAVPLGANYRL--IDIPVSNCLN--SNV--SKIYVLT--QFNS--ASLNRHLSRA--YASNMGGYKNE
10   SRS--V--LGIILG--GGAGTRLYPLTKKRAKPAVPLGANYRL--IDIPVSNCLN--SNV--SKIYVLT--QFNS--ASLNRHLSRA--YASNMGGYKNE
11   SRS--V--LGIILG--GGAGTRLYPLTKKRAKPAVPLGANYRL--IDIPVSNCLN--SNI--SKIYVLT--QFNS--ASLNRHLSRA--YASNMGGYKNE
12   SRS--V--LGIILG--GGAGTRLYPLTKKRAKPAVPLGANYRL--IDIPVSNCLN--SNI--SKIYVLT--QFNS--ASLNRHLSRA--YASNMGGYKNE
13   SRS--V--LGIILG--GGAGTRLYPLTKKRAKPAVPLGANYRL--IDIPVSNCLN--SNI--SKIYVLT--QFNS--ASLNRHLSRA--YASNMGGYKNE
14   SRS--V--LGIILG--GGAGTRLYPLTKKRANRAVPLGANYRL--IDIPVSNCLN--SNI--SKIYVLT--QFNS--ASLNRHLSRA--YASNMGGYKNE
16   SRS--V--LGIILG--GGAGTRLYPLTKKRAKPAVPLGANYRL--IDIPVSNCLN--SNI--SKIYVLT--QFNS--ASLNRHLSRA--YASNLGGYKNE
17   SRS--V--LGIILG--GGAGTRLYPLTKKRAKPAVPLGANYRL--IDIPVSNCLN--SNI--SKIYVLT--QFNS--ASLNRHLSRA--YASNLGGYKNE
18   SRS--V--LGIILG--GGAGTRLYPLTKKRAKPAVPLGANYRL--IDIPVSNCLN--SNV--SKIYVLT--QFNS--ASLNRHLSRA--YASNMGGYKNE
19   SRS--V--LGIILG--GGAGTRLYPLTKKRAKPAVPLGANYRL--IDIPVSNCLN--SNI--SKIYVLT--QFNS--ASLNRHLSRA--YASNLGGYKNE
20   SRS--V--LGIILG--GGAGTRLYPLTKKRAKPAVPLGANYRL--IDIPVSNCLN--SNI--SKIYVLT--QFNS--ASLNRHLSRA--YASNMGGYKNE
21   SRS--V--LGIILG--GGAGTRLYPLTKKRAKAAVPLGANYRK--IDIPVSNCLN--SNI--SKIYVLT--QFNS--ASLNRHLSRA--YA-VIWWLQNE
22   SRR--V--LGIILG--GGAGTRLYPLTKKRAKPAVPLGANYRL--IDIPVSNCLN--SNI--SKIYVLT--QFNS--ASLNRHLSRA--YASNMGGYKNE
23   SRS--V--LGIILG--GGAGTRLYPLTKKRAKPAVPLGANYRL--IDIPVSNCLN--SNI--SKIYVLT--QFNS--ASLNRHLSRA--YASNMGGYKNE
24   SRS--V--LGIILG--GDGRTRLYPLTKKRAKPAVPLGANYRL--IDIPVSNCLN--SNV--SKIYVLT--QFNS--ASLNRHLSRA--YASNMGGYKNE
25   SRS--V--LGIILG--GGAGTRLYPLTKKRAKPAVPLGANYRL--IDIPVSNCLN--SNI--SKIYVLT--QFNS--ASLIRHLSRA--YASNMGGYKNE
26   SRS--V--LGIILG--GGAGTRLYPLTKKRAKPAVPLGANYRL--IDIPVSNCLN--SNV--SKIYVLT--QFNS--ASLIRHLSRA--YASNMGGYKNE
27   NFS--E--LAFFLE---WSGTRLYLLRKKRAKPAVPIGANYRL--NDIPVSNCLN--SNV--SKIYVLT--QFNS--AFLNRHLSRA--YASNMGGYKNE
28   SRS--V--LGIILG--SGAGTKLYPLTKKRAKPAVPFGANYRL--IDIPVSNCLN--SNI--SKIYVLT--QFNS--ASLNRHLSRA--YASNMGGYKNE
29   SKS--V--LGIILG--GGAGTRLYPLTKKRAKPAVPLGANYRL--IDIPVSNCLN--SNV--SKIYVLT--QFNS--ASLNRHLSRA--YASNMGGYKNE
30   SRS--V--LGIILG--GGAGTRLYPLTKKRAKPAVPLGANYRL--IDIPVSNCLN--SNI--SKIYVLT--QFNS--ASLNRHLSRA--YASNLGGYKNE
31   SRS--V--LGIILG--GGAGTRLYPLTKKRAKPAVPLGANYRL--IDIPVSNCLN--SNI--SKIYVLT--QFNS--ASLNRHLSRA--YASNLGGYKNE
32   STS--V--LGIILG--GGAGTRLYPLTKKRAKPAVPLGANYRL--IDIPVSNCLN--SNV--SKIYVLT--QFNS--ASLNRHLSRA--YGNNIAGYKNE
```

```
33   HDS--V--LGIILG--GGAGTRLYPLTKKRAKPAVPLGANYRL--IDIPVSNCLN--SNI--SKIYVLT--QFNS--ASLNRHLSRA--YGSNIGGYKNE
34   STS--V--LGIILG--GGAGTRLYPLTKKRAKPAVPLGANYRL--IDIPVSNCLN--SNI--SKIYVLT--QFNS--ASLNRHLSRA--YGSNIGGYKNE
36   STS--V--LGIILG--GGAGTRLYPLTKKRAKPAVPLGANYRL--IDIPVSNCLN--SNI--SKIYVLT--QFNS--ASLNRHLSRA--YGNNIGGYKND
37   DES--V--LGIILG--GGAGTRLYPLTKKRAKPAVPLGANYRL--IDIPVSNCLN--SNI--SKIYVLT--QFNS--ASLNRHLSRA--YGNNIGGYKNE
38   STS--V--LGIILG--GGAGTRLYPLTKKRAKPAVPLGANYRL--IDIPVSNCLN--SNV--SKIYVLT--QFNS--ASLNRHLSRA--YGNNIGGYKNE
39   STS--V--LGIILG--GGAGTRLYPLTKKRAKPAVPLGANYRL--IDIPVSNCLN--SNI--SKIYVLT--QFNS--ASLNRHLSRA--YGNNIGGYKNE
41   NDS--V--LGIILG--GGAGTRLYPLTKKRAKPAVPLGANYRL--IDIPVSNCLN--SNI--SKIYVLT--QFNS--ASLNRHLSRA--YGSNIGGYKNE
42   STS--V--LGIILG--GGAGTRLYPLTKKRAKPAVPLGANYRL--IDIPVSNCLN--SNI--SKIYVLT--QFNS--ASLNRHLSRA--YGSNIGGYKNE
43   STS--V--LGIILG--GGAGTRLYPLTKKRAKPAVPLRANYRL--IDIPVSNCLN--SNV--SKIYVLT--QFNS--ASLNRHLSRA--YGNNIAGYKNE
44   STS--V--LGIILG--GGAGTRLYPLTKKRAKPAVPLGANYRL--IDIPVSNCLN--SNV--SKIYVLT--QFNS--ASLNRHLSRA--YGNNIGGYKND
45   IDS--V--LGIILG--GGAGTRLYPLTKKRAKPAVPLGANYRL--IDIPVSNCLN--SNI--SKIYVLT--QFNS--ASLNRHLSRA--YGSNIGGYXNE
46   STS--V--LGIILG--GGAGTRLYPLTKKRAKPAVPLGANYRL--IDIPVSNCLN--SNI--SKIYVLT--QFNS--ASLNRHLSRA--YGSNIGGYKNE
47   STS--V--LGIILG--GGAGTRLYPLTKKRAKPAVPLGANYRL--IDIPVSNCLN--SNV--SKIYVLT--QFNS--ASLNRHLSRA--YGNNIGGYKND
48   DDS--V--LGIILG--GGAGTRLYPLTKKRAKPAVPLGANYRL--IDIPVSNCLN--SNI--SKIYVRT--QFNS--ASLNRHLSRA--YGSNIGGYKNE
49   PKD--V--AAVILG--GGEGTKLFPLTSRTATPAVPVGGCYRL--IDIPMSNCIN--SAI--NKIFVLT--QYNS--APLNRHIART--Y-FGNGVSFGD
50   PKA--V--ASVILG--GGVGTRLFPLTSRRAKPAVPIGGCYRL--IDVPMSNCIN--SGI--RKIFILT--QFNS--FSLNRHLA-T--YNFGNGVGFGD
51   ART--V--VAIILG--GGAGTRLFPLTKRRAKPAVPMGGAYRL--IDVPMSNCIN--SGI--NKVYILT--QFNS--ASLNRHIARA--YNFGNGVTFES
52   PRT--V--ASIILG--GGAGTRLFPLTKRRAKPAVPIGGAYRL--IDVPMSNCIN--SGI--NKVYILT--QYNS--ASLNRHLARA--YNS-NGLGFGD
53   PKN--V--ASIILG--GGAGTRLFPLTSKRAKPAVPIGGCYRL--IDIPMSNCIN--SGI--RKIFILT--QFNS--FSLNRHLSRT--YNFGNGVNFGD
54   PKN--V--AAIILG--GGDGAKLFPLTKRAATPAVPVGGCYRM--IDIPMSNCIN--SCI--NKIFVLT--QFNS--ASLNRHLART--Y-FGNGINFGD
55   PQN--V--AAIILG--GGNGAKLFPLTMRAATPAVPVGGCYRL--IDIPMSNCIN--SCI--NKIFVLT--QFNS--ASLNRHLART--Y-FGNGINFGG
56   PKD--V--AAVILG--GGEGTKLFPLTSRTATPAVPVGGCYRL--IDIPMSNCIN--SAI--NKIFVLT--QYNS--AALNRHIART--Y-FGNGVSFGD
57   PKA--V--ASVILG--GGVGTRLFPLTSRRAKPAVPIGGCYRL--IDVPMSNCIN--SGI--RKIFILT--QFNS--FSLNRHLART--YNFGNGVGFGD
58   ART--V--VAIILG--GGGGTRLFPLTKRRAKPAVPIGGAYRL--IDVPMSNCIN--SGI--NKVYILT--QFNS--ASLNRHIARA--YNFGNGVTFGD
59   PKD--V--AAVILG--GGEGTKLFPLTSRTATPAVPVGGCYRL--IDIPMSNCIN--SAI--NKIFVLT--QYNS--AALNRHIART--Y-FGNGVSFGD
60   PKA--V--ASVILG--GGVGTRLFPLTSRRAKPAVPIGGCYRV--IDVPMSNCIN--SGI--RKIFILT--QFNS--FSLNRHLART--YNFGNGVGFGD
61   SRT--V--ASIILG--GGAGTRLFPLTKRRAKPAVPIGGAYRL--IDVPMSNCIN--SGI--NKVYILT--QYNS--ASLNRHLARA--YNS-NGVGFGD
62   PKN--V--ASIILG--GGAGTRLFPLTSKRAKPAVPIGGCYRL--IDIPMSNCIN--SGI--RKIFILT--QFNS--FSLNRHLSCT--YNFGNGVNFGD
63   PKN--V--AAIILG--GGDGAKLFPLTKRAATPAVPVGGCYRM--IDIPMSNCIN--SSI--NKIFVLT--QFNS--ASLNRHLART--Y-FGNGINFGD
64   PQN--V--AAIILG--GGNGAKLFPLTMRAATPAVPVGGCYRL--IDIPMSNCIN--SCI--NKIFVLT--QFNS--ASLNRHLART--Y-FGNGINFGG
66   PKT--V--ASIILG--GGAGTRLFPLTQRRAKPAVPFGGCYRL--VDIPMSNCIN--SGI--NKIYVLT--QFNS--QSLNRHIART--YNWGGCINFGG
67   PRT--V--LAVILG--GGAGTRLFPLTKRRAKPAVPIGGAYRL--IDVPMSNCIN--SGI--NKVYILT--QFNS--ASLNRHIARA--YNSGNGVTFGD
68   PEN--V--AAIILG--GGAGTRLFPLTSTRAKQAVPIAGCYRL--IDIPMSNCIN--SGI--RKVYVLT--QFNS--FSLNGHLSRT--YNFGNGVNFGG
69   PEN--V--VAIILG--GGAGTRLFPLTSTRAKQAVPIAGCYRL--IDIPMSNCIN--SGI--RKVYVLT--QFNS--FSLNGHLSRT--YNFGNGVNFGG
70   PKN--V--VSIILG--GGPGIQLFPLTKRAATPAVPVGGCYRL--IDIPMSNCIN--SGL--NKIFVLT--QFNS--ASLNRHISRT--Y-FGNGINFGD
71   PKN--V--VSVILG--GGPGIQLFPLTKRAATPAVPVGGCYRL--IDIPMSNCIN--SGL--NKIFVLT--QFNS--ASLNRHISRT--Y-FGNGINFGD
72   PRT--V--LAVILG--GGAGTRLFPLTKRRAKPAVPIGGAYRL--IDVPMSNCIN--SGI--NKVYILT--QFNS--ASLNRHIARA--YNSGNGVTFGD
73   PKS--V--ASIILG--GGAGTRLFPLTGRRAKPAVPIGGCYRL--IDIPMSNCIN--SGI--RKIFILT--QFNS--FSLNRHLSRA--YSFGNGMTFGD
```

74	PKN--V--VSIILG--GGPGTQLFPLTKRAATPAVPVGGCYRL--IDIPMSNCLN--SGI--NKIFVLT--QFNS--ASLNRHIART--Y-FGNGINFGD
75	PKS--V--ASIILG--GGAGTRLFPLTGRRAKPAVPIGGCYRL--IDIPMSNCIN--SGI--RKIFILT--QFNS--FSLNRHLSRA--YSFGNGITFGD
76	PKN--V--ISIILG--GGPGTQLFPLTKRAATPAVPVGGCYRL--IDIPMSNCLN--SGI--NKIFVLT--QFNS--ASLNRHIART--Y-FGNGINFGD
77	PKT--V--ASIILG--GGAGTRLFPLTQRRAKPAVPFGGCYRL--VDIPMSNCIN--SGI--NKIYVLT--QFNS--QSLNRHIAQT--YNLGGCINFGG
80	PKN--V--ASIVLG--GGPGVQLFPLTKRAATPAVPVGGCYRL--IDIPMSNCIN--SGI--NKIFVLT--QFNS--ASLNRHIART--Y-FGNGINFGD
81	PKT--V--AAIILG--GGAGTRLFPLTRRRAKPAVPIGGCYRL--IDVPMSNCIN--SGI--NKIYILT--QFNS--QSLNRHIART--YNQGNGVDFGD
82	PKN--V--ASIILG--GGAGTQLFPLTRRAATPAVPLGGCYRL--IDIPMSNCIN--SGI--NKIFVLT--QFNS--TSLNRHLART--Y-FGNGIIFGD
84	PRT--V--VAVILG--GGAGTRLFPLTKRRAKPAVPIGGSYRL--IDVPMSNCIN--SGI--NKVYILT--QYNS--ASLNRHLARA--YNLGNGVSFGD
87	PRT--V--VAIILG--GGAGTRLFPLTKRRAKPAVPIGGAYRL--IDVPMSNCIN--SGI--NKVYILT--QFNS--ASLNRHLARA--YNFGNGINFGD
88	PKT--V--ASIILG--GGAGTRLFPLTRTRAKPAVPIGGCYRL--IDVPMSNCIN--SGI--NKIYILT--QFNS--QSLNRHIART--YNSGNGVNFGD
89	PKN--V--ASIILG--GGAGTQLFPLTRRAATPAVPVGGCYKL--IDIPMSNCIN--SGI--NKIFVLT--QFNS--ASLNRHLART--Y-FGNGINFGD
90	PKK--V--ASIILG--GGAGTRLFPLTSKRAKPAVPIGGCYRL--IDIPMSNCIN--SGI--RKIFIMT--QFNS--FSLNRHLART--YNFGNGVNFGD
91	PSS--V--AAIILG--GGAGTRLFPLTSRRAKPAVPIGGCYRL--IDVPMSNCIN--SGI--RKIFILT--QFNS--ASLNRHIARI--YNFGNGVNFGD
92	PKT--V--ASIILG--GGAGTRLFPLTKSRAKPAVPIGGCYRL--IDVPMSNCIN--SGI--NKIYILT--QFNS--QSLNRHIART--YNLGSGVNFGD
94	PRT--V--VAVILG--GGAGTRLFPLTKRRAKPAVPIGGSYRL--IDVPMSNCIN--SGI--NKVYILT--QFNS--ASLNRHLARA--YNFGHGVNFGD
95	PKN--V--ASIILG--GGAGTQLFPLTIRQATPAVPVGGCYRL--IDIPMSNCIN--SNI--NKIFILT--QFNS--ASLNRHIART--Y-FGNGVNFGD
96	PGN--V--ASIILG--GGAGTRLFPLTSKRAKPAVPIGGCYRL--IDIPMSNCIN--SGI--KKIFILT--QFNS--FSLNRHIART--YNLGNGVSFGD
97	PKN--V--ASIILG--GGAGTQLFPLTRRAATPAVPVGGCYRL--IDIPMSNCIN--SGI--NKIFVLT--QFNS--ASLNRHLAHT--Y-FGNGINFGD
98	PSN--V--ASIILG--GGAGTRLFPLTSRRAKPAVPIGGCYRL--IDIPMSNCIN--SGI--KKIFILT--QFNS--FSLNRHLART--YNFGNGVSFGD
99	SKT--V--VAVILG--GGAGTRLFPLTRRRAKPAVPIGGAYRL--IDVPMSNCIN--SGI--NKVYILT--QFNS--QSLNRHLSRA--YDFSNGVAIGD
100	ANR--V--SAIILG--GGTGSQLFPLTSTRATPAVPVGGCYRL--IDIPMSNCFN--SGI--NKIFVMS--QFNS--TSLNRHIHRT--YLE-GGINFAD
101	PNE--V--AAVILG--GGTGTQLFPLTSTRATPAVPIGGCYRL--IDIPMSNCFN--SGI--NKIFVMT--QFNS--ASLNRHIHRT--YLG-GGINFTD
102	PDT--V--ASIILG--GGAGTRLFPLTRTRAKPAVPVGGCYRL--IDIPMSNCIN--SKI--NKIYVLT--QFNS--QSLNRHIART--YNFGEGVGFSG
103	PNE--V--AAVILG--GGTGTQLFPLTSTRATPAVPIGGCYRL--IDIPMSNCFN--SGI--NKIFVMT--QFNS--ASLNRHIHRT--YLG-GGINFTD
104	PDT--V--ASIILG--GGAGTRLFPLTRTRAKPAVPVGGCYRL--IDIPMSNCIN--SKI--NKIYVLT--QFNS--QSLNRHIART--YNFGEGVGFGN
105	ART--V--VAVILG--GGAGTRLFPLTKRRAKPAVPIGGAYRL--IDVPMSNCIN--SGI--NKVYVLT--QFNS--ASLNRHLSRA--YNFSNGVGFGD
106	SKT--V--VAVILG--GGAGTRLFPLTKRRAKPAVPIGGAYRL--IDVPMSNCIN--SGI--NKVYILT--QFNS--ASLNRHLSRA--YNFSNGVAFGD
107	PNE--V--AAVILG--GGTGTQLFPLTSTRATPAVPIGGCYRL--IDIPMSNCFN--SGI--NKIFIMT--QFNS--ASLNRHIHRT--YLG-GGINFTD
108	PDT--V--ASIILG--GGAGTRLFPLTRTRAKPAVPVGGCYRL--IDIPMSNCIN--SKI--NKIYVLT--QFNS--QSLNRHIART--YNIGEGVGFGD
109	ASH--V--SAVILG--GGTGVQLFPLTSTRATPAVPVGGCYRL--IDIPMSNCFN--SGI--NKIFVMT--QFNS--ASLNRHIHHT--YLG-GGINFTD
110	PNE--V--AAVILG--GGTGTQLFPLTSTRATPAVPIGGCYRL--IDIPMSNCFN--SGI--NKIFVMT--QFNS--ASLNRHIHRT--YLG-GGINFTD
111	ANH--V--SAIILG--GGTGSQLFPLTSTRATPAVPVGGCYRL--IDIPMSNCFN--SGI--NKIFVMT--QFNS--TSLNRHIHRT--YLG-GEINFAD
112	SRT--V--VAVILG--GGAGTRLFPLTKRRAKPAVPIGGAYRL--IDVPMSNCIN--SGI--NKVYILT--QFNS--QSLNRHLSRA--YDCTNGVAFGD
113	PNE--V--AAVILG--GGTGTQLFPLTSTRATPAVPIGGCYRL--IDIPMSNCFN--SGI--NKIFVMT--QFNS--ASLNRHIHRT--YLG-GGINFTD
114	PRT--V--VAVILG--GGAGTRLFPLTKRRAKPAVPIGGAYRL--IDVPMSNCIN--SGI--NKVYVLT--QFNS--ASLNRHLFRA--YNFSNGVGFGD
115	RPS--V--AAVILG--GGTGTQLFPLTSTRATPAVPIGGCYRL--IDIPMSNCFN--SGI--NKIFVMT--QFNS--ASLNRHIHRT--YLG-GGINFTD
116	PRT--V--VAVILG--GGAGTRLFPLTRQRAKPAVPIGGAYRL--IDVPMSNCIN--SGI--NKVYILT--QFNS--ASLNRHLLRA--YNFSNGIGFGD
117	PNE--V--AAVILG--GGTGTQLFPLTSTRATPAVPIGGCYRL--IDIPMSNCFN--SGI--NKIFVMT--QFNS--ASLNRHIHRT--YLG-GGINFTD

```
118 PRT--V--VAVILG--GGAGTRLFPLTKRRAKPAVPIGGAYRL--IDVPMSNCIN--SGI--NKVYVLT--QFNS--ASLNRHLSRA--YNFSNGVGFGD
119 PNE--V--AAVILG--GGTGTQLFPLTSTRATPAVPIGGCYRL--IDIPMSNCFN--SGI--NKIFVMT--QFNS--ASLNRHIHRT--YLG-GGINFTD
120 PNE--V--AAVILG--GGTGTQLFPLTSTRATPAVPIGGCYRL--IDIPMSNCFN--SGI--NKIFVMT--QFNS--ASLNRHIHRT--YLG-GGINFTD
121 MKR--V--LAIILG--GGAGTRLYPLTKMRAKPAVPLAGKYRL--IDIPISNCIN--SNI--TKMYVLT--QFNS--ASLNRHLSQT--YDLSA--GFGQ
122 MKQ--V--LAVILG--GGAGTRLYPLTKMRAKPAVPLAGKYRL--IDIPISNCIN--SEI--LKIYILT--QFNS--ASLNRHIART--YNFS---GFTD
123 MKK--V--LAIILG--GGAGTRLYPLTKMRAKPAVPVAGKYRL--IDIPVSNCIN--SEI--FKIYVLT--QFNS--ASLNRHIART--YNFT---GFNE
124 MKK--V--LAIILG--GGAGTRLYPLTKLRAKPAVPLAGKYRL--IDIPVSNCIN--AEI--LKIYVLT--QFNS--ASLNRHLTRT--YNFT---GFHD
125 MNN--V--LSIILG--GGAGTRLYPLTKTRAKPAVPLAGKHRL--IDIPISNCIN--SSI--HKIYVLT--QFNS--ASLNQHISRS--YNFS---GFQQ
126 MKR--V--LAIILG--GGAGTRLYPLTKMRAKPAVPLAGKYRL--IDIPISNCIN--SGI--NKMYVLT--QFNS--ASLNRHLTQS--YNLSS--GFGQ
127 MKR--V--LAIILG--GGAGTRLYPLTKMRAKPAVPLAGKYRL--IDIPISNCIN--SDI--NKMYVLT--QFNS--ASLNRHLTQS--YNLSS--GFGQ
128 MKR--V--LAIILG--GGAGTRLYPLTKMRAKPAVPLAGKYRL--IDIPISNCIN--SGI--NKIYVLT--QFNS--ASLNRHLTQS--YNLSA--GFGQ
129 MKK--V--LSIILG--GGAGTRLYPLTKLRAKPAVPLAGKYRL--IDIPISNCIN--SEI--LKIYVLT--QFNS--ASLNRHISRA--YNFS---GFTD
130 MKR--V--LAIILG--GGAGTRLYPLTKMRAKPAVPLAGKYRL--IDIPISNCIN--SSI--TKMYVLT--QFNS--ASLNRHLSQT--YDLSA--GFGQ
131 MKR--V--LAIILG--GGAGTRLYPLTKMRAKPAVPLAGKYRL--IDIPISNCIN--SDI--HKMYVMT--QFNS--ASLNRHLSQT--FNLSN--SFGG
132 MKR--V--LAIILG--GGAGTRLYPLTKMRAKPAVPLAGKYRL--IDIPISNCIN--SNI--NKMYVLT--QFNS--ASLNRHLSQT--YNLSA--GFGQ
133 MRQ--V--TAIILG--GGRGTRLYPLTKRRAKPAVPIGGKYRL--IDIPVSNCIN--SGI--QHIYILT--QFNS--ASLNRHVSQT--YQFS---RFSD
134 MKR--V--LAIILG--GGKGSRLYPLTKMRAKPAVPLAGKYRL--IDIPISNCIN--SGI--NKMYVLT--QFNS--ASLNRHIGRT--YNLSA--PFGQ
135 MKR--V--LAIILG--GGKGSRLYPLTKMRAKPAVPLAGKYRL--IDIPISNCIN--SNI--TKMYVLT--QFNS--ASLNRHLAQT--YNLSS--PFAQ
136 MKR--V--LAIILG--GGAGTRLYPLTKRRAKPAVPLAGKYRL--IDIPVSNCIN--SEI--HNIYVLT--QFNS--ASLNRHIART--YTFP---GLTG
137 MKK--V--LAIILG--GGAGTRLYPLTKLRAKPAVPLAGKYRL--IDIPLSNCIN--SEI--LKIYVLT--QFNS--ASLNRHLTRT--YNFT---GFSD
138 MKK--V--LSIILG--GGAGTRLYPLTKLRAKPAVPVAGKYRL--IDIPVSNCIN--SEI--FKIYVLT--QFNS--ASLNRHIART--YNFT---GFNE
139 MKR--V--LAIILG--GGAGTRLYPLTKMRAKPAVPLAGKYRL--IDIPISNCIN--SNI--NKMYVLT--QFNS--ASLNRHLGQS--YNLSA--AFGQ
140 MRD--V--LAIILG--GGRGTRLYPLTKRRAKPAVPLAGKYRL--IDIPVSNCIN--SDI--EKIYVLT--QFNS--ASLNRHIVNT--YRLS---PFTG
141 MRD--V--LAIILG--GGRGTRLYPLTKRRAKPAVPLAGKYRL--IDIPVSNCIN--SDI--DKIYVLT--QFNS--ASLNRHIINT--YRMS---PFTG
142 MKR--V--LAIILG--GGAGTRLYPLTKMRAKPAVPLAGKYRL--IDIPISNCIN--SSI--NKMYVLT--QFNS--ASLNRHLGQS--YNLSA--AFGQ
143 MKR--V--LAIILG--GGAGTRLYPLTKMRAKPAVPLAGKYRL--IDIPISNCIN--SNI--NKMYVMT--QFNS--ASLNRHLSQT--FNLSA--SFGQ
144 MKK--V--LAIILG--GGAGTRLYPLTKLRAKPAVPVAGKYRL--IDIPVSNCIN--SEI--FKIYVLT--QFNS--ASLNRHIART--YNFS---GFSE
145 MKR--V--LAIILG--GGKGSRLYPLTKMRAKPAVPLAGKYRL--IDIPISNCIN--SNI--HKMYVLT--QFNS--ASLNRHISQT--YNLSS--PFAQ
146 MKR--V--LAIILG--GGKGSRLYPLTKMRAKPAVPLAGKYRL--IDIPISNCIN--SGI--KKMYVLT--QFNS--ASLNRHIGRT--YNLNG--PFGQ
147 MKR--V--LAIILG--GGKGSRLYPLTKMRAKPAVPLAGKYRL--IDIPISNCIN--SDI--SKMYVLT--QFNS--ASLNRHIAQT--YNLSG--PFGQ
148 MKR--V--LAIILG--GGKGSRLYPLTKMRAKPAVPLAGKYRL--IDIPISNCIN--SGI--EKMYVLT--QFNS--ASLNRHIGRT--YNLNG--PFGQ
149 MKR--V--LAIILG--GGKGSRLYPLTKMRAKPAVPLAGKYRL--IDIPISNCIN--SGI--NKMYVLT--QFNS--ASLNRHIGRT--YNLSA--PFGQ
150 MKR--V--LAIILG--GGAGTRLQPLTKMRAKPAVPLAGKYRL--IDIPISNCIN--SSI--NKMYVLT--QFNS--ASLNRHLSQT--YNLNA--GFGQ
151 MKR--V--LAIILG--GGAGTRLYPLTKLRAKPAVPLAGKYRL--IDIPVSNCIN--SEI--TKIYVLT--QFNS--ASLNRHLSRT--YNFT---GFND
152 MKR--V--LAIILG--GGKGSRLYPLTKMRAKPAVPLAGKYRL--IDIPISNCIN--SGI--EKMYVLT--QFNS--ASLNRHIGRT--YNLNG--PFGQ
153 VKR--V--LAIILG--GGAGTRLYPLTKLRAKPAVPLAGKYRL--IDIPVSNCIN--SEI--VKIYVLT--QFNS--ASLNRHISRA--YNFS---GFQE
154 MKK--V--LAIILG--GGAGTRLYPLTKLRAKPAVPLAGKYRL--IDIPLSNCIN--SEI--LKIYVLT--QFNS--ASLNRHLTRT--YNFT---GFSD
155 MKN--V--LAIILG--GGAGSRLYPLTKQRAKPAVPLAGKYRL--IDIPVSNCIN--ADI--NKIYVLT--QFNS--ASLNRHLSQT--YNLSS--GFGN
```

```
156  MKK--V--LAIILG--GGAGTRLYPLTKQRAKPAVPLAGKYRL--IDIPVSNCIN--SEI--THVYVLT--QFNS--ASLNRHIART--YNFS---GFSD
157  MKN--V--LAIILG--GGAGSRLYPLTKQRAKPAVPLAGKYRL--IDIPVSNCIN--ADI--NKIYVLT--QFNS--ASLNRHLSQT--YNLSS--GFGN
158  MKR--V--LAIILG--GGAGTRLYPLTKMRAKPAVPLAGKYRL--IDIPISNCIN--SDI--NKMYVMT--QFNS--ASLNRHLSQT--YNLSN--SFGG
159  MKR--V--LAIILG--GGAGTRLYPLTKMRAKPAVPLAGKYRL--IDIPISNCIN--SDI--HKMYVMT--QFNS--ASLNRHLSQT--FNLSN--SFGG
160  MKR--V--LSIILG--GGAGTRLYPLTKLRAKPAVPLAGKYRL--IDIPVSNCIN--SDI--NKIYVLT--QFNS--ASLNRHLSRG--YNFS---NFTE
161  MNR--V--LAIVLG--GGAGTRLYPLTKQRAKPAVSLAGKYRL--IDIPMSNCIN--SEI--NKIYVMT--QFNS--ASLNRHISQT--YHFS---SFSD
162  MKN--V--LSIILG--GGAGTRLYPLTKLRAKPAVPLAGKYRL--IDIPISNCIN--SEI--QKIYVLT--QFNS--ASLNRHITRT--YNFS---GFSD
163  MKK--V--LAIILG--GGAGTRLYPLTKLRAKPAVPVAGKYRL--IDIPVSNCIN--SEI--FKIYVLT--QFNS--ASLNRHIART--YNFS---GFSE
164  MNN--V--LSIILG--GGAGTRLYPLTKTRAKPAVPLAGKHRL--IDIPISNCIN--SNL--LKIYVLT--QFNS--ASLNQHISRS--YNFS---GFQQ
165  MKK--V--LAIILG--GGAGTRLYPLTKLRAKPAVPLAGKYRL--IDIPVSNCIN--AEI--LKIYVLT--QFNS--ASLNRHLTRT--YNFT---GFHD
166  MKR--V--LGIILG--GGAGTRLYPLTKLRAKPAVPLAGKYRL--IDIPVSNCIN--SEI--HKIYILT--QFNS--ASLNRHISRT--YNFT---GFTE
167  MKR--V--LAIILG--GGKGSRLYPLTKMRAKPAVPLAGKYRL--IDIPISNCIN--SGI--EKMYVLT--QFNS--ASLNRHIGRT--YNLNG--PFGQ
168  MKR--V--LAIILG--GGKGSRLYPLTKMRAKPAVPLAGKYRL--IDIPISNCIN--SDI--SKMYVLT--QFNS--ASLNRHIAQT--YNLSG--PFGQ
169  MKR--V--LAIILG--GGAGTRLYPLTKMRAKPAVPLAGKYRL--IDIPVSNCIN--SGI--NKIYVLT--QFNS--ASLNRHIAQT--FNLSS--GFDQ
170  MKR--V--LAIILG--GGAGTRLYPLTKMRAKPAVPLAGKYRL--IDIPISNCIN--SNI--NKMYVLT--QFNS--ASLNRHLSQT--YNLSA--GFGQ
171  MKK--V--LAIILG--GGAGTRLYPLTKLRAKPAVPLAGKYRL--IDIPVSNCIN--SQI--DKIYVLT--QFNS--ASLNRHLNRT--YNFT---GFSD
172  MKR--V--LAIILG--GGKGSRLYPLTKMRAKPAVPLAGKYRL--IDIPISNCIN--SGI--EKMYVLT--QFNS--ASLNRHIGRT--YNLNG--PFGQ
173  MKR--V--LAIILG--GGAGTRLYPLTKMRAKPAVPLAGKYRL--IDIPISNCIN--SEI--NKIYVLT--QFNS--ASLNRHLSMS--YNLSA--GFGQ
174  MKK--V--LSIILG--GGAGTRLYPLTKLRAKPAVPLAGKYRL--IDIPVSNCIN--SEI--TKIYVLT--QFNS--ASLNRHLSRT--YNFN---GFND
175  MKK--V--LAIILG--GGAGTRLYPLTKLRAKPAVPVAGKYRL--IDIPVSNCIN--SEI--FKIYVLT--QFNS--ASLNRHIARA--YNFS---GFSD
176  MKK--V--LGIILG--GGAGSRLYPLTKPRAKPAVSLAGKYRL--IDIPVSNCIN--SEI--YKIYVLT--QFNS--ASLNRHITRA--YNFS---GFTE
177  MKK--V--LAIILG--GGAGTRLYPLTKLRAKPAVPLAGKYRL--IDIPVSNCIN--SEI--FKIYVLT--QFNS--ASLNRHIART--YSFA---GFTE
178  MKK--V--LAIILG--GGAGTRLYPLTKLRAKPAVPLAGKYRL--IDIPVSNCIN--AEI--LKIYVLT--QFNS--ASLNRHLTRT--YNFT---GFHD
179  MNN--V--LSIILG--GGAGTRLYPLTKTRAKPAVPLAGKHRL--IDIPISNCIN--SNL--LKIYVLT--QFNS--ASLNQHISRS--YNFS---GFQQ
180  MKR--V--LAIILG--GGAGTRLQPLTKMRAKPAVPLAGKYRL--IDIPISNCIN--SSI--NKMYVLT--QFNS--ASLNRHLSQT--YNLNA--GFGQ
181  MKK--V--LAIILG--GGAGTRLYPLTKLRAKPAVPLAGKYRL--IDIPISNCIN--AKI--QKIYVLT--QFNS--ASLNHHLTHT--YNFG---PFSG
182  MKK--V--LAIILG--GGVGTRLYPLTKLRAKPAVPLAGKYRL--IDIPVSNCIN--SEI--VKIYVLT--QFNS--ASLNRHISRT--YQFS---GFTE
183  MKK--V--LAIILG--GGVGTRLYPLTKLRAKPAVPLAGKYRL--IDIPVSNCIN--SEI--LKIYVLT--QFNS--ASLNRHISRA--YNFS---GFSD
184  MKR--V--LAIILG--GGAGTRLYPLTKLRAKPAVPVAGKYRL--IDIPVSNCIN--SEI--FKIYVLT--QFNS--ASLNRHIARA--YNFS---GFSD
185  MKR--V--LAIILG--GGAGTRLYPLTKLRAKPAVPVAGKYRL--IDIPVSNCIN--SEI--FKIYVLT--QFNS--ASLNRHIARA--YNFS---GFSD
186  VKQ--V--LAVILG--GGAGTRLYPLTKMRAKPAVPLAGKYRL--IDIPISNCIN--SEI--LKIYILT--QFNS--ASLNRHIART--YNFS---GFTD
187  MKK--V--LAIILG--GGAGTRLYPLTKLRAKPAVPLAGKYRL--IDIPISNCIN--AEI--LKIYVLT--QFNS--ASLNRHLTRT--YNFT---GFSD
188  MKR--V--LAIILG--GGAGTRLYPLTKMRAKPAVPLAGKYRL--IDIPISNCIN--SDI--NKMYVMT--QFNS--ASLNRHLSQT--YNLSN--SFGG
189  SKS--V--AAVILG--GGAGTRLYPLTKSRAKPAVPIGGAYRL--IDVPMSNCLN--SGI--SKMYILT--QFNS--VSLNRHLART--YNFGNGIMYGG
190  TDN--V--LAIILG--GGAGTRLYPLTKKRAKPAVPLGANYRL--IDIPVSNCIN--SDI--NKMYCLT--QFNS--ASLNRHLSQA--YNSNVGSGLRQ
191  SKS--V--AAVILG--GGAGTRLYPLTKSRAKPAVPIGGAYRL--IDVPMSNCLN--SGI--SKMYILT--QFNS--VSLNRHLART--YNFGNGIMYGG
192  TDN--V--LGIILG--GGAGTRLYPLTKTRAKPAVPLGANYRL--IDLPVSNCIN--SDI--NKMYCLT--QFNS--ASLNRHLSQA--YNNNVGSYNRQ
193  GEV--C--SSIILG--GGAGTRLFPLTKSRAKPAVPIGGAYRL--IDVPMSNCIN--SGI--SKIYILT--QFNS--TSLNRHLGRA--YNMGSGVRFGG
```

```
194  SKT--V--LGIILG--GGAGTRLYPLTKKRAKPAVPLGANYRL--IDIPVSNCLN--SNV--TKIYCLT--QFNS--ASLNRHLSQA--YNSSVGGYNSR
195  TKT--V--AAVILG--GGAGTRLYPLTKSRAKPAVPIGGAYRL--IDVPMSNCLN--SGI--SKVYILT--QFNS--ASLNRHLART--YNFGNGIMYGG
196  MDN--V--LSIILG--GGAGTRLYPLTKKRAKPAVPLGANYRL--IDIPVSNCIN--SDI--NKVYCLT--QFNS--ASLNRHLAQA--YNTNIGTHTRQ
197  SKT--V--LGIILG--GGAGTRLYPLTKKRAKPAVPLGANYRL--IDIPVSNCLN--SNV--TKIYCLT--QFNS--ASLNRHLSQA--YNSSVGGYNTR
198  TNT--V--LSIILG--GGAGTRLFPLTKQRAKPAVPIGGAYRL--IDVPMSNCIN--SGI--SKIYILT--QFNS--TSLNRHLARA--YNMGSGVRFGG
199  SKT--V--AAVILG--GGAGTRLYPLTKSRAKPAVPIGGAYRL--IDVPMSNCLN--SGI--SKVYILT--QFNS--ASLNRHLART--YNFGNGIMYGG
200  MDN--V--LSIILG--GGAGTRLYPLTKKRAKPAVPLGANYRL--IDIPVSNCIN--SDI--NKVYCLT--QFNS--ASLNRHLSQA--YNTNIGTYTRQ

                  110       120       130       140       150       160       170       180       190       200
     ....|....|....|....|....|....|....|....|....|....|....|....|....|....|....|....|....|....|....|....|
1    G---FVEVLAA--QQS--PENPDWFQG--TADAVR--QYL--WLFE--EH----TV--LEYLILAGDHL--YRMDY--EKFIQAHRE--TDA--DITVAA
2    G---LVEVLAA--QQS--PENPDWFQG--TADAVR--QYL--WLFE--EH----TV--LEYLILAGDHL--YRMDY--EKFIQAHRE--TDA--DITVAA
3    G---FVEVLAA--QQS--PENPNWFQG--TADAVR--QYL--WLFE--EH----NV--LEYLILAGDHL--YRMDY--EKFIQAHRE--TDA--DITVAA
4    G---FVEVLAA--QQS--PENPDWFQG--TADAVR--QYL--WLFE--EH----TV--LEYLILAGDHL--YRMDY--EKFIQAHRE--TDA--DITVAA
5    G---FVEVLAA--QQS--PENPNWFQG--TADAVR--QYL--WLFE--EH----NV--LEYLILAGDHL--YRMDY--EKFIQAHRE--TDA--DITVAA
6    G---FVEVLAA--QQS--PENPNWFQV--TADAVR--QYL--WLFE--EH----NV--LEYLILAGDHL--YRMDY--EKFIQAHRE--TDA--DITVAA
7    G---FVEVLAA--QQS--PENPNWFQG--TADAVR--QYL--WLFE--EH----NV--LEYLILAGDHL--YRMDY--EKFIQAHRE--TDA--DITVAA
8    G---FVEVLAA--QQS--PENPNWFQG--TADAVR--QYL--WLFE--EH----NV--LEYLVLAGDHL--YRMDY--ERFIQAHRE--SDA--DITVAA
9    G---FVEVLAA--QQS--PENPNWFQG--TADAVR--QYL--WLFE--EH----NV--LEFLVLAGDHL--YRMDY--EKFIQAHRE--TDA--DITVAA
10   G---FVEVLAA--QQS--PENPNWFQG--TADAVR--QYL--WLFE--EH----NV--LEFLVLAGDHL--YRMDY--EKFIQAHRE--TDA--DITVAA
11   G---FVEVLAA--QQS--PENPNWFQG--TADAVR--QYL--WLFE--EH----NV--LEFLVLAGDHL--YRMDY--ERFIQAHRE--TDA--DITVAA
12   G---FVEVLAA--QQS--PENPNWFQG--TADAVR--QYL--WLFE--EH----NV--LEFLILAGDHL--YRMDY--ERFIQAHRE--TDA--DITVAA
13   G---FVEVLAA--QQS--PENPNWFQG--TADAVR--QYL--WLFE--EH----NV--LEFLVLAGDHL--YRMDY--ERFIQAHRE--TDA--DITVAA
14   G---FVEVLAA--QQS--PENPNWFQG--TADAVR--QYL--WLFE--EH----NV--LEFLVLAGDHL--YRMDY--EKFIQAHRE--TDA--DITVAA
16   G---FVEVLAA--QQS--PENPNWFQG--TADAVR--QYL--WLFE--EH----NV--LEYLVLAGDHL--YRMDY--ERFIQAHRE--SDA--DITVAS
17   G---FVEVLAA--QQS--PENPNWFQG--TADAVR--QYL--WLFE--EH----NV--LEYLILAGDHL--YRMDY--EKFIQAHRE--SDA--DITVAA
18   G---FVEGLAA--QQS--PENPNWFQG--TADAVR--QYL--WLFE--EH----NV--LEYLVLAGDHL--YRMDY--ERFIQAHRE--SDA--DITVAA
19   G---FVEVLAA--QQS--PENPNWFQG--TADAVR--QYL--WLFE--EH----NV--LEFLILAGDHL--YRMDY--EKFIQAHRE--TDA--DITVAA
20   G---FVEVLAA--QQS--PENPNWFQG--TADAVR--QYL--WLFE--EQ----NV--LEYLVLAGDHL--YRMDY--ERFIQAHRE--TDA--DITVAA
21   G---FVEVLAA--QQS--PENPNWFQG--TADAVR--QYL--WLFE--EQ----NV--LEYLVLAGDHL--YRMDY--ERFIQAHRE--TDA--DITVAA
22   G---FVEVLAA--QQS--PENPNWFQG--TADAVR--QYL--WLFE--EH----NV--LEFLVLAGDHL--YRMDY--ERFIQAHRE--TDA--DITVAA
23   G---FVEVLAA--QQS--PENPNWFQG--TADAVR--QYL--WLFE--EH----NV--LEFLVLAGDHL--YRMDY--ERFIQAHRE--TDA--DITVAA
24   G---FVEVLAA--QQS--PENPNWFQG--TADAVR--QCL--WLFE--EH----NV--LEFLVLAGDHL--YRMDY--EKFIQAHRE--TDA--DITVAA
25   G---FVEVLAA--QQS--PENPNWFQG--TADAVR--QYL--WLFE--EH----NV--LEFLVLAGDHL--YRMDY--ERFIQAHRE--TDA--DITVAA
26   G---FVEVLAA--QQS--PENPNWFQG--TADAVR--QYL--WLFE--EH----NV--LEFLVLAGDHL--YRMDY--ERFIQAHRE--TDA--DITVAA
27   G---FVEVFAA--QQS--PENPNWFQG--TADAVR--QYL--WLFE--EH----DV--LEYLVLAGDHL--YRMDY--EKFVQSHRE--TDA--DITVAA
28   G---FVEVLAA--QQS--PENPNWFQG--TADAVR--QYL--WLFE--EH----NV--LEFLILAGDHL--YRMDY--ERFIQAHRE--TDA--DITVAA
29   G---FVEVLAA--QQS--PENPNWFQG--TADAVR--QYL--WLFE--EH----NV--LEYLVLAGDHL--YRMDY--EKFIQVHRE--SDA--DITVAA
```

```
30   G---FVEVLAA--QQS--PENPNWFQG--TADAVR--QYL--WLFE--EH----NV--LEYLVLAGDHL--YRMDY--ERFIQAHRE--SDA--DITVAA
31   G---FVEVLAA--QQS--PENPNWFQG--TADAVR--QYL--WLFE--EH----NV--LEYLILAGDHL--YRMDY--EKFIQAHRE--SDA--DITVAA
32   G---FVEVLAA--QQS--PENPNWFQG--TADAVR--QYM--WLFE--EH----NI--MEFLILAGDHL--YRMDY--QKFIQAHRE--TDA--DITVAA
33   G---FVEVLAA--QQS--PDNPNWFQG--TADAVR--QYL--WLFE--EH----NV--MEFLILAGDHL--YRMDY--EKFIQAHRE--TNA--DITVAA
34   G---FVEVLAA--QQS--PDNPNWFQG--TADAVR--QYL--WLFE--EH----NV--MEFLILAGDHL--YRMDY--EKFIQAHRE--TNA--DITVAA
36   G---FVEVLAA--QQS--PDNPNWFQG--TADAVR--QYL--WLFE--EH----NV--MEFLILAGDHL--YRMDY--EKFIQAHRE--TDA--DITVAA
37   G---FVEVLAA--QQS--PDNPNWFQG--TADAVR--QYL--WLFE--EH----NV--MEFLILAGDHL--YRMDY--EKFIQAHRE--TDS--DITVAA
38   G---FVEVLAA--QQS--PENPNWFQG--TADAVR--QYL--WLFE--EH----NV--MEFLILAGDHL--YRMDY--QKFIQAHRE--TNA--DITVAA
39   G---FVEVLAA--QQS--PDNPNWFQG--TADAVR--QYL--WLFE--EH----NV--MEFLILAGDHL--YRMDY--EKFIQAHRE--TDS--DITVAA
41   G---FVEVLAA--QQS--PDNPNWFQG--TADAVR--QYL--WLFE--EH----NV--MEYLILAGDHL--YRMDY--EKFIQAHRE--TDA--DITVAA
42   G---FVEVLAA--QQS--PDNPNWFQG--TADAVR--QYL--WLFE--EH----NV--MEYLILAGDHL--YRMDY--EKFIQAHRE--TDA--DITVAA
43   G---FVEVLAA--QQS--PENPNWFQG--TADAVR--QYM--WLFE--EH----NI--MEFLILAGDHL--YRMDY--QKFIQAHRE--TDA--DITVAA
44   G---FVEVLAA--QQS--PENPNWFQG--TADAVR--QYL--WLFE--EH----NV--MEFLILAGDHL--YRMDY--QKFIQAHRE--TDA--DITVAA
45   G---FVEVLAA--QQS--PDNPDWFQG--TADAVR--QYL--WLFE--EH----NV--MEYLILAGDHL--YRMDY--EKFIQAHRE--TDA--DITVAA
46   G---FVEVLAA--QQS--PDNPDWFQG--TADAVR--QYL--WLFE--EH----NV--MEYLILAGDHL--YRMDY--EKFIQAHRE--TDA--DITVAA
47   G---FVEVLAA--QQS--PESPDWFQG--TADAVR--QYL--WLFE--EH----NV--MEFLILAGDHL--YRMDY--QKFIQAHRE--TDA--DITVAA
48   G---FVEVLAA--QQS--PDNPDWFQG--TADAVR--QYL--WLFE--EH----NV--MEYLILAGDHL--YRMDY--EKFIQAHRE--TDA--DITVAA
49   G---FVEVLAA--TQTPGEAGKKWFQG--TADAVR--KFI--WVFE--DAK-NKNI--ENIVVLSGDHL--YRMDY--MELVQNHID--RNA--DITLSC
50   G---FVEVLAG--TQTPGDGRKMWFQ---AADAVR--EFI--WVFE--NQK-NKNV--EHIIILSGDHL--YRMNY--MDFVQKHID--TNA--DITVSC
51   G---YVEVLAA--TQTPGELGKRWFQG--TAHAVR--QFH--WLFE--DAR-SKDI--EDVLILSGDHL--YRMDY--LHFVQSHRQ--SGA--DITISS
52   G---YVEVLAA--TQTPGESGKRWFQG--TADAVR--QFH--WLFE--DAR-SKDI--EDVLILSGDHL--YRMDY--MDFIQDHRQ--SGA--DISISC
53   G---FVEVLAA--TQTSGDAGKKWFQG--TADAVR--QFI--WVFE--DAK-TKNV--EHVLILSGDHL--YRMDY--MNFVQKHIE--SNA--DITVSC
54   G---FVEVLAA--TQTPGEAGKKWFQG--TADAVR--KFL--WVFE--DAK-NRNI--ENIIILSGDHL--YRMNY--MDFVQHHVD--SKA--DITLSC
55   G---FVEVLAA--TQTPGEAGKKWFQG--TADAVR--KFL--WVFE--DAK-NRNI--ENILILSGDHL--YRMNY--MDFVQSHVD--SNA--DITLSC
56   G---FVEVLAA--TQTPGEAGKKWFQG--TADAVR--KFI--WVFE--DAK-NKNI--ENILVLSGDHL--YRMDY--MELVQNHID--RNA--DITLSC
57   G---FVEVLAA--TQTPGDAGKMWFQG--TADAVR--QFI--WVFE--NQK-NKNV--EHIIILSGDHL--YRMNY--MDFVQKHID--ANA--DITVSC
58   G---YVEVLAA--TQTPGELGKRWFQG--TADAVR--QFH--WLFE--DAR-SKDI--EDVLILSGDHL--YRMDY--LHFVQSHRQ--SGA--DITISS
59   G---FVEVLAA--TQTPGEAGKKWFQG--TADAVR--KFI--WVFE--DAK-NKNI--ENILVLSGDHL--YRMDY--MELVQNHID--RNA--DITLSC
60   G---FVEVLAA--TQTPGDAGKMWFQG--TADAVR--QFI--WVFE--NQK-NKNV--EHIIILSGDHL--YRMNY--MDFVQKHID--ANA--DITVSC
61   G---YVEVLAA--TQTPGESGKRWFQG--TADAVR--QFH--WLFE--DAR-SKDI--EDVLILSGDHL--YRMDY--MDFVQDHRQ--SGA--DISISC
62   G---FVEVLAA--TQTSGDAGKKWFQG--TADAVR--QFI--WVFE--DAK-TKNV--EHVLILSGDHL--YRMDY--MNFVQKHIE--SNA--DITVSC
63   G---FVEVLAA--TQTPGEAGKKWFQG--TADAVR--KFL--WVFE--DAK-NRNI--ENILILSGDHL--YRMNY--MDFVQYHVD--SKA--DITLSC
64   G---FVEVLAA--TQTPGEAGKMWFQG--TADAVR--KFL--WVFE--DAK-NRNI--ENILILSGDHL--YRMNY--MDFVQSHVD--SNA--DITLSC
66   G---FVEVLAA--TQTPGESGKKWFQG--TADAVR--QFL--WLFE--DAD-HKNI--ENILILCGDQL--YRMDY--MEIVQKHIN--SCA--DISVSC
67   G---YVEVLAA--TQTPGEAGKKWFQG--TADAVR--QFH--WLFE--DPR-SKDI--EDVLILSGDHL--YRMDY--MDFVQNHRE--SGA--DITLSC
68   G---FVEVLAA--TKTPGESGNKWFQG--TADAVR--RFI--WVFE--DAK-NKDI--ENILIISGDHL--CRMDY--MKLLEKHIG--TNA--DITVSC
69   G---FVEVLAA--TLTNGEAGNKWFQG--TADAVR--RFS--WVFE--DAK-NKNI--EHILIISGDHL--CRMDY--MKLVEKHIG--TNA--DITVSC
70   G---CVEVLAA--TQTQGETGKNWFQG--TADAVR--QFT--WVFE--DAK-HTNI--ENVLILAGDHL--YRMDY--MDLVQSHVD--RNA--DITVSC
```

71 G---CVEVLAA--TQTQGEAGNNWFQG--TADAVR--QFT--WVFE--DAK-HANI--ENVLILAGDHL--YRMNY--MDLVQSHVD--RNA--DITVSC
72 G---YVEVLAA--TQTPGEAGKKWFQG--TADAVR--QFH--WLFE--DPR-SKDI--EDVLILSGDHL--YRMDY--MDFVQNHRE--SGA--DITLSC
73 G---FVEVLAA--TQTPGEAGKKWFQG--TADAVR--QFI--WVFE--DAK-NKNV--EHILILSGDHL--YRMDY--MDFVQRHVD--TNA--DITVSC
74 G---IVEVLAA--TQTPGEAGKNWFQG--TADAVR--QFT--WVFE--DAK-NTNV--ENVLILAGDHL--YRMDY--MDLVQSHVD--RNA--DITVSC
75 G---FVEVLAA--TQTPGEAGKKWFQG--TADAVR--QFI--WVFE--DAK-NKNV--EHILILSGDHL--YRMDY--MNFVQRHVD--TNA--DITVSC
76 G---IVEVLAA--TQTPGEAGKNWFQG--TADAVR--QFT--WVFE--DAK-NTNV--ENVLILAGDHL--YRMDY--MDLVQSHVD--RNA--DITVSC
77 G---FVEVLAA--TQTPGESGKKWFQG--TADAVR--QFL--WLFE--DAD-HKNI--ENILILCGDQL--YRMDY--MEIVQKHIN--SCA--DISVSC
80 G---YVEVLAA--TQTPGEAGKNWFQG--TADAVR--QFT--WVFE--DAK-NTNI--ENVIILAGDHL--YRMDY--MDLVQSHID--RNA--DITVSC
81 G---FVEVLAA--TQTPGESGKKWFQG--TADAVR--QFI--WLFE--DAK-LRNI--ENILVLSGDHL--YRMDY--MDFLQKHIE--SGA--DICVSC
82 G---FVEVLAA--TQTPGEAGMKWFQG--TADAVR--QFT--WVFE--DAK-NRNI--ENILVLSGDHL--YRMDY--MDFVQHHID--SNA--DFTISC
84 G---FVEALAA--TQTPGEAGKKWFQG--TADAVR--QFH--WLFE--GPR-SKEI--EDVLILSGDHL--YRMDY--MDFVQNHRQ--GGA--DITLSC
87 G---FVEVLAA--TQTPGEAGKRWFQG--TADAVR--QFH--WLFE--DAR-SKDI--DDVLVLSGDHL--YRMDY--MDFVQNHRQ--SGA--DITISC
88 G---FVEVLAA--TQTPGESGKKWFQG--TADAVR--QFL--WLFE--DAK-HSHI--ENILILSGDHL--YRMDY--MDFLQKHID--SGA--DITVSC
89 G---FVEVLAA--TQTPGEAGMNWFQG--TADAVR--QFT--WVFE--DAK-NRNV--ENILILSGDHL--YRMDY--MDFVQHHVD--SNA--DITISC
90 G---FVEVLAA--TKTPGEAGNKWFQG--TADAVR--QFI--WVFE--DAK-NKNV--ENVLILSGDHL--YRMDY--MEFVQKHID--SGA--DITVSC
91 G---FVEVLAA--TQTPGEAGQKWFQG--TADAVR--QFI--WVFE--DAK-NKNV--EHILILSGDHL--YRMDY--MDFVQKHID--SNA--DITVSC
92 G---FVEVLAA--TQTSGESGKKWFQG--TADAVR--QFI--WLFE--DAR-HRHI--ENILILSGDHL--YRMDY--MEFVQKHID--SDA--DISVSC
94 G---YVEALAA--TQTPGEAGKRWFQG--TADAVR--QFH--WLFE--DQR-SKEI--EDVLILSGDHL--YRMDY--MDFVQNHRQ--SGA--DITISC
95 G---FVEVLAA--TQTPGEAGMKWFEG--TADAVR--KFI--WVFE--DAK-NKNI--ENILILSGDHL--YRMDY--MDLVQNHID--RKA--DITVSC
96 G---FVEVLAA--TQTPGETGKKWFQG--TADAVR--QFI--WVFE--DAR-NKNV--EHVLILSGDHL--YRMNY--MEFVQKHID--TNA--DVTVSC
97 G---FVEVLAA--TQTPGEAGMKWFQG--TADAVR--QFT--WVFE--DAK-NRSI--ENILILSGDHL--YRMDY--MDFVQHHVD--SNA--DITISC
98 G---FVEVLAA--TQTPGEAGKKWFQG--TADAVR--QFI--WMFE--DAR-TKNV--EHVLILSGDHL--YRMNY--MEFVQKHID--TNA--DITVSC
99 G---FVEVLAA--TQRPGTEGKRWFQG--TADAVR--QFD--WLFD--DAK-SKDI--EDVLILSGDHL--YRMDY--MDFVQSHRQ--RGA--GISICC
100 G---SVQVLAA--TQMPEEPAG-WFQG--TADSIR--KFI--WVLE--DYYSHKSI--DNIVILSGDQL--YRMNY--MELVQKHVE--DDA--DITISC
101 G---SVEVLAA--TQMPGEAAG-WFQG--TADAVR--KFI--WVLE--DYYKHKAI--EHILILSGDQL--YRMDY--MELVQKHVD--DNA--DITLSC
102 G---SVEVLAA--TQTAGESGKKWFQG--TADAVR--QFL--WLFE--DAR-LKCI--ENILILSGDHL--YRMDY--MDFVQKHVD--SGA--DISVAC
103 G---SVEVLAA--TQMPGEAAG-WFRG--TADAVR--KFI--WVLE--DYYKHKSI--EHILILSGDQL--YRMDY--MELVQKHVD--DNA--DITLSC
104 G---FVEVLAA--TQTAGESGKRWFQG--TADAVR--QFL--WLFE--DAR-LKRI--ENILILSGDHL--YRMDY--MDFVQKHVD--SGA--DISVAC
105 G---FVEVLAA--TQRPGLEGKRWFQG--TADAVR--QFD--WLFD--DAK-AKDI--EDVIILSGDHL--YRMDY--MDFVQSHRQ--RDA--GISICC
106 G---FVEVLAA--TQTPGSEGKRWFQG--TADAVR--QFD--WLFD--DAK-AKDI--DDVLILSGDHL--YRMDY--MDFVQSHRQ--RGA--DISICC
107 G---SVEVLAA--TQMPGEAAG-WFQG--TADAVR--KFI--WVLE--DYYKHKAI--EHILILSGDQL--YRMDY--MELVQKHVD--DNA--DITLSC
108 G---FVEVLAA--TQTTGESGKRWFQG--TADAVR--QFL--WLFE--DAR-LKRI--ENILILSGDHL--YRMDY--MDFVQKHVD--KGA--DISVAC
109 G---SVQVLAA--TQMPDEPAG-WFQG--TADAIR--KFM--WILE--DHYNQNNI--EHVVILCGDQL--YRMNY--MELVQKHVD--DNA--DITISC
110 G---SVEVLAA--TQMPGEAAG-WFQG--TADAVR--KFI--WVLE--DYYKHKAI--EHILILSGDQL--YRMDY--MELVQKHVD--DNA--DITLSC
111 G---SVQVLAD--TQMPEEPDG-WFQG--TADSVR--KFI--WVLE--DYYNHKSI--EHIVILSGDQL--YQMNY--MELVQKHVE--DNA--DITVSC
112 G---FVEVLAA--TQRPGSEGKRWFQG--TADAVR--QFD--WLFD--DAK-SKDI--DDVLILSGDHL--YRMDY--MDFVQSHRQ--RGA--GISICC
113 G---SVEVLAA--TQMPGEAAG-WFRG--TADAVR--KFI--WVLE--DYYKHKSI--EHILILSGDQL--YRMDY--MELVQKHVD--DNA--DITLSC
114 G---FVEVLAA--TQRPGSEGKRWFQG--TADAVR--QFA--WLFD--DAK-SKDI--EDVLILSGDHL--YRMDY--MDFVQSHRQ--RDA--GISICC

```
115  G---SVEVLAA--TQMPGEAAG-WFRG--TADAVR--KFI--WVLE--DYYKHKSI--EHILILSGDQL--YRMDY--MELVQKHVD--DNA--DITLSC
116  G---FVEVLAA--TQTPGEAGKKWFQG--TADAVR--QFH--WLFE--DAK-GKEI--EDVLILSGDHL--YRMDY--MDFVQSHRQ--SGA--DITISC
117  G---SVEVLAA--TQMPGEAAG-WFRG--TADAVR--KFI--WVLE--DYYKNKSI--EHILILSGDQL--YRMDY--MELVQKHVD--DNA--DITLSC
118  G---FVEVLAA--TQRPGSEGKTWFQG--TADAVR--QFA--WLFD--DAK-SKDI--EDVLILSGDHL--YRMDY--MDFVQSHRQ--RDA--GISICC
119  G---SVEVLAA--TQMPGEAAG-WFRG--TADAVR--KFI--WVLE--DYYKNKSI--EHILILSGDQL--YRMDY--MELVQKHVD--DNA--DITLSC
120  G---SVEVLAA--TQMPGEAAG-WFRG--TADAWR--KII--WVLE--DYYKNKSI--EHILILSGDQL--YRMDY--MELVQKHVD--DNA--DITLSC
121  G---FVEVLAA--QQTP-ESPS-WFEG--TADAVR--KYQ--WLFQ--EW----DV--DEYLILSGDQL--YRMDY--SLFVEHHRQ--SGA--DLTVAA
122  G---FAEVLAA--QQT-SVTNPQWFQG--TADAVR--QYL--WLME--EW----DV--EHFLILSGDHL--YRMDY--RDFVQRHID--TGA--DITLSV
123  G---FVEVLAA--QQT--PENPNWFQG--TADAVR--QYL--WLLN--EW----DA--DEYLILSGDHL--YRMDY--RQFIQRHRE--TGA--DITLSV
124  G---FVEVLAA--QQT--TENPSWFQG--TADAVR--QYG--WLFD--EW----DV--DEYLILSGDHL--YRMDY--SDFVKRHRE--TGA--DITLSV
125  G---FVEILAA--QQT--PENMNWFQG--TADAVR--QYL--WLFD--RA----EA--DEYLILSGDHL--YRMDY--RDFIQHHRE--TNA--DISLSV
126  G---FVEVLAA--QQT--PESPSWFEG--TADAVR--KYQ--WLFQ--EW----DV--DHYLILSGDQL--YRMDY--STFVDHHIA--TGA--DVSIGA
127  G---FVEVLAA--QQT--PESPSWFEG--TADAVR--KYQ--WLFQ--EW----DV--DHYLILSGDQL--YRMDY--SEFVNHHIA--TGA--DISIGA
128  G---FVEVLAA--QQT--PDSPTWFEG--TADAVR--KYQ--WLFQ--EW----DV--DQYLILSGDQL--YRMDY--SRFVDHHIQ--SGA--DLTVGA
129  G---FTEVLAA--QQT--ASNPNWFQG--TADAVR--QYI--WLFA--EW----DV--DYFLILSGDHL--YRMDY--REFVQRHID--TKA--DITLSV
130  G---FVEVLAA--QQT--PESPSWFEG--TADAVR--KYQ--WLFQ--EW----DV--DEYLILSGDQL--YRMDY--SLFVEHHRK--SGA--DLTVAA
131  G---FVEVLAA--QQT--PDSPTWFEG--TADAVR--KYQ--WLFQ--EW----DV--DEYLILSGDQL--YRMDY--SLFLEHHRR--TGA--KLTVAA
132  G---FVEVLAA--QQT--PESPSWFEG--TADAVR--KYQ--WLFQ--EW----DV--DEYLILSGDQL--YRMDY--SLFINHHRS--TGA--DLTVAA
133  G---FCEILAA--EQT--DENPNWFQG--TADAVR--QYL--WLLE--PS----GS--TEYLILSGDHL--YRMDY--SKFVRRHRE--TNA--DVTIAV
134  G---FVEVLAA--QQT--PDSPKWFEG--TADAVR--KYQ--WLFQ--EW----DV--DEYLILSGDQL--YRMDY--SLFVQHHRD--NKA--DLTVAA
135  G---FVEVLAA--QQT--PESPSWFEG--TADAVR--KYQ--WLFQ--EW----DV--DEYLILSGDQL--YRMDY--SLFVEHHRE--TGA--DLTVAA
136  G---FVEVLAA--QQT--PENPNWFQG--TADAVR--QYL--WLLA--DW----DV--DEYLILSGDHL--YRMDY--RLFVQRHRD--TGA--DVTLSV
137  G---FVEVLAA--QQT--AENPKWFQG--TADAVR--QYL--WAFQ--EW----DI--DEYLILSGDHL--YRMDY--RDFIQRHRE--TGA--DITLSV
138  G---FVEVLAA--QQT--PENPNWFQG--TADAVR--QYL--WLME--EW----DV--EEYLILSGDHL--YRMDY--RQFIQRHRD--TGA--DITLSV
139  G---FVEVLAA--QQT--PESPSWFEG--TADAVR--KYQ--WLFQ--EW----DV--DEYLILSGDQL--YRMDY--SLFVEHHRR--SGA--DLTVAA
140  G---FVDVLAA--QQT--PDNPDWFQG--TADAVR--QYL--WLMD--SW----KP--RDFLILSGDHL--YRMDY--RPFIHHHRQ--VGA--DVTLAV
141  G---FVDILAA--QQT--PDNPDWFQG--TADAVR--QYL--WLMD--SW----KP--RDFLILSGDHL--YRMDY--RPFIHYHRQ--TGA--DVTLAV
142  G---FVEVLAA--QQT--PESPSWFEG--TADAVR--KYQ--WLFQ--EW----DV--DEYLILSGDQL--YRMDY--SLFVEHHRR--SGA--DLTVAA
143  G---FVEVLAA--QQT--PDSPSWFEG--TADAVR--KYQ--WLFQ--EW----DV--DEYLILSGDQL--YRMDY--SLFVEHHRS--TGA--DLTVAA
144  G---FVEVLAA--QQT--PENPNWFQG--TADAVR--QYL--WMLQ--EW----DV--DEFLILSGDHL--YRMDY--RLFIQRHRE--TNA--DITLSV
145  G---FVEVLAA--QQT--PESPSWFEG--TADAVR--KYQ--WIFQ--EW----DV--DEYLILSGDQL--YRMDY--SQFVNHHRT--TGA--DLTVAA
146  G---FVEVLAA--QQT--PDSPKWFEG--TADAVR--KYQ--WLFQ--EW----DV--DEYLILSGDQL--YRMDY--SLFVQHHRD--NGA--DLTVAA
147  G---FVEVLAA--QQT--PETPSWFEG--TADAVR--KYQ--WLFQ--EW----DV--DEYLILSGDQL--YRMDY--SLFVEQHRK--TGA--DLTVAA
148  G---FVEVLAA--QQT--PDSPKWFEG--TADAVR--KYQ--WLFQ--EW----DV--DEYLILSGDQL--YRMDY--SLFVQHHRD--NGS--DLTVAA
149  G---FVEVLAA--QQT--PDSPKWFEG--TADAVR--KYQ--WLFQ--EW----DV--DEYLILSGDQL--YRMDY--SLFVQHHRD--NGA--DLTVAA
150  G---FVEVLAA--QQT--LDSPSWFEG--TADAVR--QYQ--TLFR--EW----DV--DEYLILSGDQL--YRMDY--SRFVEHHRS--TGA--DLTVAA
151  E---FVEVLAA--QQT--AENPSWFQG--TADAVR--QYL--WLME--EW----DV--DEYLILSGDHL--YRMDY--REYIQRHRE--TKA--DITLSV
152  G---FVEVLAA--QQT--PDSPKWFEG--TADAVR--KYQ--WLFQ--EW----DV--DEYLILSGDQL--YRMDY--SLFVQHHRD--NGA--DLTVAA
```

153 G---FVEVLAA--QQT--KDNPDWFQG--TADAVR--QYL--WLFR--EW----DV--DEYLILSGDHL--YRMDY--AQFVKRHRE--TNA--DITLSV
154 G---FVEVLAA--QQT--AENPKWFQG--TADAVR--QYL--WAFQ--EW----DI--DEYLILSGDHL--YRMDY--RDFIQRHRE--TGA--DITLSV
155 G---FVEVLAA--QIT--PENPNWFQG--TADAVR--QYL--WLIK--EW----DV--DEYLILSGDHL--YRMDY--SQFIQRHRD--TNA--DITLSV
156 G---FVEVLAA--QQT--PENPDWFQG--TADAVR--QYL--WLLS--DW----EV--DYYLILSGDHL--YRMDY--RLFVNRHRD--TNA--DITLSV
157 G---FVEVLAA--QIT--PENPNWFQG--TADAVR--QYL--WLIK--EW----DV--DEYLILSGDHL--YRMDY--SQFIQRHRD--TNA--DITLSV
158 G---FVEVLAA--QQT--PDSPTWFEG--TADAVR--KYQ--WLFQ--EW----DV--DEYLILSGDQL--YRMDY--SLFIGHHRR--SGA--DLTVAA
159 G---FVEVLAA--QQT--PDSPSWFEG--TADAVR--KYQ--WLFQ--EW----DV--DEYLILSGDQL--YRMDY--SLFLEHHRR--TGA--NLTVAA
160 G---FVEVLAA--QQT--PDNSGWFEG--TADAVR--QYL--QLLK--EW----DV--DEYLILSGDHL--YRMDY--SRFVQRHRD--TNA--DITISV
161 G---FAEVLAA--QQT--PENPNWFQG--TADAVR--QYM--WMFA--EQR---DV--DEILILSGDHL--YRMDY--SVFIERHRS--TNA--DITLSV
162 G---FVEVLAA--QQT--KDNPEWFQG--TADAVR--KYI--WLFK--EW----DI--DYYLILSGDHL--YRMDY--RDFVQRHID--TKA--DITLSV
163 G---FVEVLAA--QQT--PENPNWFQG--TADAVR--QYL--WMLQ--EW----DV--DEFLILSGDHL--YRMDY--RLFIQRHRE--TNA--DITLSV
164 G---FVEILAA--QQT--PENMNWFQG--TADAVR--QYL--WLFD--RA----EA--DEYLILSGDHL--YRMDY--RDFIQHHRK--TNA--DITLSV
165 G---FVEVLAA--QQT--TENPSWFQG--TADAVR--QYG--WLFD--EW----DV--DEYLILSGDHL--YRMDY--SDFVKRHRE--TGA--DITLSV
166 G---FTEVLAA--QQT--KENPDWFQG--TADAVR--QYS--WLLE--DW----DV--DEYIILSGDHL--YRMDY--REFIQRHRD--TGA--DITLSV
167 G---FVEVLAA--QQT--PDSPKWFEG--TADAVR--KYQ--WLFQ--EW----DV--DEYLILSGDQL--YRMDY--SLFVQHHRD--NEA--DLTVAA
168 G---FVEVLAA--QQT--PETPSWFEG--TADAVR--KYQ--WLFQ--EW----DV--DEYLILSGDQL--YRMDY--SLFVEQHRN--TGA--DLTVAA
169 G---FVEVLAA--QQT--PDSPSWFEG--TADAVR--KYE--WLLQ--EW----DI--DEVLILSGDQL--YRMDY--AHFVAQHRA--SGA--DLTVAA
170 G---FVEVLAA--QQT--PESPSWFEG--TADAVR--KYQ--WLFQ--EW----DV--DEYLILSGDQL--YRMDY--SLFINHHRS--TGA--DLTVAA
171 G---FVEVLAA--QQT--MENPQWFQG--TADAVR--QYI--WTMK--DW----DI--DEYLILSGDHL--YRMDY--SKFIERHRE--TNA--DITLSV
172 G---FVEVLAA--QQT--PDSPKWFEG--TADAVR--KYQ--WLFQ--EW----DV--DEYLILSGDQL--YRMDY--SLFVQHHRD--NEA--DLTVAA
173 G---FVEVLAA--QQT--PDSPSWFEG--TADAVR--KYQ--WLFQ--EW----DV--DHYLILSGDQL--YRMDY--SRFVQHHID--TGA--DLSVGA
174 E---FVEVLAA--QQT--PENPGWFQG--TADAVR--QYL--WLME--EW----DI--DEYLILSGDHL--YRMDY--RQFIQRHRE--TNA--DITLSV
175 G---FVEVLAA--QQT--PENPNWFQG--TADAVR--QYI--WMLQ--DW----DV--DEFLILSGDHL--YRMDY--RLFIQRHRE--TNA--DITLSV
176 G---FVEILPA--QKT--AENPSWFQG--TADAVR--QYL--WLFN--GW----DV--DEYLILSGDHL--YRMDY--RLFVQRHRD--TGA--DITLSV
177 G---FVEVLAA--QQT--PENLSWFQG--TADAVR--QYL--WLFE--EW----DV--DEYLILSGDHL--YRMDY--RQFIQRHRE--TGA--DITLSV
178 G---FVEVLAA--QQT--TENPSWFQG--TADAVR--QYG--WLFD--EW----DV--DEYLILSGDHL--YRMDY--SDFVKRHRE--TGA--DITLSV
179 G---FVEILAA--QQT--PENMNWFQG--TADAVR--QYL--WLFD--RA----EA--DEYLILSGDHL--YRMDY--RDFIQHHRK--TNA--DITLSV
180 G---FVEVLAA--QQT--LDSPSWFEG--TADAVR--QYQ--TLFS--EW----DV--DEYLILSGDQL--YRMDY--SRFVEHHRS--TGA--DLTVAA
181 G---FVEVLAA--QQT--KENPSWFQG--TADAVR--QYL--WLFN--EW----DV--DEYLILSGDHL--YRMDY--DDFIKQHRI--TGA--DITLAV
182 G---FVEVLAA--QQT--QENPNWFQG--TADAVR--QYL--SLLE--QW----DV--DEYLILSGDHL--YRMDY--QKFVQRHRE--TNA--DITLSV
183 G---FVEVLAA--QQT--KENPNWFQG--TADAVR--QYL--WLFE--EW----DI--DHYLILSGDHL--YRMDY--REFVQRHLD--TKA--DITLSV
184 G---FVEVLAA--QQT--PENPNWFQG--TADAVR--QYI--WMLQ--EW----DV--DEFLILSGDHL--YRMDY--RLFIQRHRE--TNA--DITLSV
185 G---FVEVLAA--QQT--PENPNWFQG--TADAVR--QYI--WMLE--EW----DV--DEFLILSGDHL--YRMDY--RLFVQRHRE--TNA--DITLSV
186 G---FAEVLAA--QQT-SVTNPQWFQG--TADAVR--QYL--WLME--EW----DV--EHFLILSGDHL--YRMDY--RDFVQRHID--TGA--DITLSV
187 G---FVEVLAA--QQT--AENPSWFQG--TADAVR--QYL--WLFD--EW----DV--DQYLILSGDHL--YRMDY--SDFVRRHQE--TGA--DITLSV
188 G---FVEVLAA--QQT--PDSPTWFEG--TADAVR--KYQ--WLFQ--EW----DV--DEYLILSGDQL--YRMDY--SLFIEHHRR--SGA--DLTVAA
189 SG--FVEVLAA--TQTPGLGGKEWFQG--TADAVR--QYS--WLFE--DVK-NKDV--QDVVILSGDHL--YRMDY--MAFVDRHRE--VNA--DITIGC
190 G---FVEVLAA--QQS--PKSKVWFQG--TADAVR--QYM--WLFN--ES----KC--EEYIILSGDHL--YRMDY--KPFILEHRK--TGA--DITVSA

```
191   NG--FVEVLAA--TQTPGLGGKEWFQG--TADAVR--QYS--WLFE--DIK-NKDV--QDIVILSGDHL--YRMDY--MAFVARHRE--VNA--DITIGC
192   G---FVEVLAA--QQS--PKNKDWFQG--TADAVR--QYI--WLFN--ES----KC--DEYIILSGDHL--YRMDY--KPFILKHRQ--TKA--DITVSA
193   DG--FVEVLAA--TQTP-TD-KEWFQG--TADAVR--QYS--WLLE--DTK-NRAI--EDVLILSGDHL--YRMDY--MKFVNYHRE--TNA--DITIGC
194   G---FVEVLAA--SQS--SANKSWFQG--TADAVR--QYM--WLFE--EAV-REGV--EDFLILSGDHL--YRMDY--RDFVRKHRN--SGA--AITIAA
195   NG--FVEVLAA--TQTPGQGGKEWFQG--TADAVR--QYS--WLFN--DVK-NKDV--EDIVILAGDHL--YRMDY--MKFVEAHRE--SNA--DISVGT
196   G---FVEVLAA--QQS--PVNKAWFQG--TADAVR--QYL--WLFE--ES----KC--EEYLILSGDHL--YRMDY--RPFIMKHRE--TEA--AITVAA
197   G---FVEVLAA--SQS--SANKSWFQG--TADAVR--QYM--WLFE--EAV-REGV--EDFLILSGDHL--YRMDY--RDFVRKHRE--SGA--AITIAA
198   DG--FVEVLAA--TQTP-TD-KEWFQG--TADAVR--QYS--WLLE--DTK-NRAI--EDVLILSGDHL--YRMDY--MKFVNYHRE--TNA--DITIGC
199   NG--FVEVLAA--TQTPGQGGKEWFQG--TADAVR--QYS--WLFN--DVK-NKDV--EDIVILAGDHL--YRMDY--MKFVEAHRE--SNA--DITVGT
200   G---FVEVLAA--QQS--PINKAWFQG--TADAVR--QYL--WLFA--ES----GC--EEYLILSGDHL--YRMDY--RPFIRDHRA--KNA--DITVAA

                     210       220       230       240       250       260       270       280       290       300
      ....|....|....|....|....|....|....|....|....|....|....|....|....|....|....|....|....|....|....|....|
1     LPM--D--EKRAT--A--FGLMKID--EE---GRIIEFAEK--PQG---EQLQA--MKVDTTILGLDDKR-AKEMP--FIASM--G---IYVISK--DVM
2     LPM--D--EKRAT--A--FGLMKID--EE---GRIIEFAEK--PQG---EQLQA--MKVDTTILGLDDKR-AKEMP--LVASM--G---IYVISK--DVM
3     LPM--D--EKRAT--A--FGLMKID--EE---GRIIEFAEK--PQG---EQLQA--MKVDTTILGLDDKR-AKEMP--FIASM--G---IYVISK--DVM
4     LPM--D--EKRAT--A--FGLMKID--EE---GRIIEFAEK--PQG---EQLQA--MKVDTTILGLDDKR-AKEMP--FIASM--G---IYVISK--DVM
5     LPM--D--EQRAT--A--FGLMKID--EE---GRIIEFAEK--PKG---EHLKA--MKVDTTILGLDDQR-AKEMP--FIASM--G---IYVVSR--DVM
6     LPM--D--EQRAT--A--FGLMKID--EE---GRIVEFAEK--PKG---EHLKA--MKVDTTILGLDDKR-AKEMP--YIASM--G---IYVVSR--DVM
7     LPM--D--EKRAT--A--FGLMKID--EE---GRIIEFSEK--PKG---DQLQA--MKVDTTILGLDDER-AKEMP--FIASM--G---IYVISK--NVM
8     LPM--D--EARAT--A--FGLMKID--EE---GRIIEFAEK--PKG---EQLKA--MKVDTTILGLDDER-AKEMP--YIASM--G---IYVVSK--HVM
9     LPM--D--EARAT--A--FGLMKID--EE---GRIIEFAEK--PKG---EQLKA--MKVDTTILGLDDER-AKEMP--YIASM--G---IYVVSK--NVM
10    LPM--D--EKRAT--A--FGLMKID--EE---GRIIEFAEK--PKG---EQLKA--MKVDTTILGLDDER-AKELP--YIASM--G---IYVVSK--NVM
11    LPM--D--EKRAT--A--FGLMKID--EE---GRIIEFAEK--PKG---EQLKA--MKVDTTILGLDDER-AKEMP--YIASM--G---IYVVSK--NVM
12    LPM--D--EKRAT--A--FGLMKID--EE---GRIIEFAEK--PKG---EQLKA--MKVDTTILGLDDER-AKEMP--YIASM--G---IYVVSK--NVM
13    LPM--D--EKRAT--A--FGLMKID--EE---GRIIEFAEK--PKG---EQLKA--MKVDTTILGLDDER-AKEMP--YIASM--G---IYVVSK--DVM
14    LPM--D--EKRST--A--FGLMKID--DE---GRIIEFAEK--PKG---EQLKA--MKVDTTILGLDDER-AKEIP--FIASM--G---IYVVSK--NVM
16    LPM--D--EARAT--A--FGLMKID--EE---GRIVEFSEK--PKG---EQLKA--MKVDTTILGLDDER-AKEMP--YIASM--G---IYVVSK--HVM
17    LPM--D--EKRAT--A--FGLMKID--EE---GRIIEFAEK--PKG---EQLKA--MKVDTTILGLDDER-AKEMP--FIASM--G---IYVISK--NVM
18    LPM--D--ELRAT--A--FGLMKID--EE---GRIIEFAEK--PKG---EQLKA--MKVDTTILGLDEER-AKEMP--YIASM--G---IYVVSK--HVM
19    LPM--D--EKRAT--A--FGLMKID--EE---GRIIEFAEK--PKG---DQLNA--MKVDTTILGLDDER-AKEMP--FIASM--G---IYVISK--NVM
20    LPM--D--EKRAT--A--FGLMKID--EE---GRIIEFAEK--PKG---EQLKT--MKVDTTILGLDDAR-GKEMP--FIASM--G---IYVISK--DVM
21    LPM--D--EKRAT--A--FGLMKID--EE---GRIIEFAEK--PKG---EQLKA--MKVDTTILGLDDER-AKEMP--YIASM--G---IYVISK--DVM
22    LPM--D--EKLAT--A--FGLMKID--EE---GRIIEFSEK--PKG---EQLKA--MKVDTTILGLDDAR-GKEMP--FIASM--G---IYVISK--DVM
23    LPM--D--EKRAT--A--FGLMKID--EE---GRIIEFSEK--PKG---EQLKA--MKVDTTILGLDDER-AKEMP--YIASM--G---IYVISK--DVM
24    LPM--D--EKRAT--A--FGLMKID--DE---GRIIEFAEK--PKG---EQLKA--MKVDTTILGLDDER-AKEMP--YIASM--G---IYVVSK--NVM
25    LPM--D--EKRAT--A--FGLMKID--EE---GRIIEFAEK--PKG---EQLKA--MKVDTTLFGLDDQR-AKEMP--YIASM--G---IYVVSK--DVM
26    LPM--D--EKRAT--A--FGLMKID--EE---GRIIEFAEK--PKG---EQLKA--MKVDTTIFGLDDQR-AKELP--FIASM--G---IYVISK--NVM
```

```
27   LPM--D--EKRAT--A--FGLMKID--DE---GRIIEFSEK--PKG---EALKA--MRVDTTILGLDDER-AKEMP--YIASM--G---IYVFSK--NAM
28   LPM--D--EKRPT--A--FGLMKID--EE---GRIIEFAEK--PKG---EQLKA--MKVDTTILGLDDKR-AKEMP--YIASM--G---IYVVSK--DVM
29   LPM--D--ENRAT--A--FGLMKID--EE---GRIIEFAEK--PKG---EQLKA--MKVDTTIFGLDDER-AKEMP--YIASM--G---IYVVSK--NVM
30   LPM--D--EARAT--A--FGLMKID--EE---GRIIEFSEN--PKG---EQLKA--MKVDTTILGLDDDR-AKEMP--YIASM--G---IYVVSK--HVM
31   LPM--D--EKRAT--A--FGLMKID--EE---GRIIEFAEK--PKG---EQLKA--MKVDTTILGLDDER-AKEMP--FIASM--G---IYVISK--NVM
32   LPM--D--EQRAT--A--FGLMKID--DE---GRIVEFAEK--PKG---EKLRS--MMVDTTILGLDPER-AKELP--YIASM--G---IYVFSK--DVM
33   LPM--D--EKRAT--A--FGLMKID--EE---GRIIEFAEK--PKG---EQLKA--MMVDTTILGLDDVR-AKEMP--YIASM--G---IYVFSK--DVM
34   LPM--D--EKRAT--A--FGLMKID--EE---GRIIEFAEK--PKG---EQLKA--MMVDTTILGLDDVR-AKEMP--YIASM--G---IYVFSK--DVM
36   LPM--D--EKRAT--A--FGLMKID--EE---GRIIEFAEK--PKG---DQLKA--MMVDTTILGLDDER-AKEMP--YIASM--G---IYVFSK--DVM
37   LPM--D--EKRAT--A--FGLMKID--EE---GRIVEFAEK--PKG---EQLKA--MMVDTTILGLDDVR-AKEMP--YIASM--G---IYVISK--NVM
38   LPM--D--EERAT--A--FGLMKID--DE---GRIIEFAEK--PKG---EKLKS--MMVDTTILGLDTER-AKELP--YIASM--G---IYVFSK--DVM
39   LPM--D--EKRAT--A--FGLMKID--EE---GRIVEFAEK--PKG---EQLKA--MMVDTTILGLDDVR-AKEMP--YIASM--G---IYVISK--NVM
41   LPM--D--EERAT--A--FGLMKID--EE---GRIIEFAEK--PKG---EQLKA--MMVDTTILGLDDVR-AKEMP--YIASM--G---IYVISK--HVM
42   LPM--D--EERAT--A--FGLMKID--EE---GRIIEFAEK--PKG---EQLKA--MMVDTTILGLDDVR-AKEMP--YIASM--G---IYVISK--HVM
43   LPM--D--EQRAT--A--FGLMKID--DE---GRIVEFAEK--PKG---EKLRS--MMVDTTILGLDPER-AKELP--YIASM--G---IYVFSK--DVM
44   LPM--D--EERAT--A--FGLMKID--DE---GRIVEFSEK--PKG---EKLKA--MMVVTTILGLDSER-AKELP--YIASM--G---IYVFSK--DAM
45   LPM--D--EERAT--A--FGLMKID--EE---GRIIEFAEK--PKG---EQLKA--MMVDTTILGLEDAR-AKEMP--YIASM--G---IYVISK--HVM
46   LPM--D--EERAT--A--FGLMKID--EE---GRIIEFAEK--PKG---EQLKA--MMVDTTILGLEDAR-AKEMP--YIASM--G---IYVISK--HVM
47   LPM--D--EERAT--A--SGLMKID--DE---GRIVEFSEK--PKG---EKLKA--MMVDTTILGLDSER-AKELP--YIASM--G---IYVFSK--DAM
48   LPM--D--EERAT--A--FGLMKID--EE---GRIIEFAEK--PKG---EQLKA--MMVDTTILGLDDAR-AKEMP--YIASM--G---IYVISK--HVM
49   APA--E--DSRAS--D--FGLVKID--SR---GRVVQFAEK--PKG---FDLKA--MQVDTTLVGLSPQD-AKKSP--YIASM--G---VYVFKT--DVL
50   VPM--D--DGRAS--D--FGLMKID--ET---GAIIQFAEK--PKG---PALKA--MQVDTSILGLSEQE-ASNFP--YIASM--G---VYVFKT--DVL
51   LPI--D--DSRAS--D--FGLMKID--DT---GRVMSFSEK--PKG---DDLKA--MAVDTTVLGLSPEE-AKEKP--YIASI--G--KVYVFKK--DIL
52   IPI--D--DRRAS--D--FGLMKID--DK---GRVISFSEK--PKG---DDLKA--MAVDTTILGLSKEE-AEKKP--YIASM--G---VYVFKK--EIL
53   LPM--D--ESRAS--D--FGLLKID--QS---GKIIQFSEK--PKG---DDLKA--MQVDTSILGLPPKE-AAESP--YIASM--G---VYVFRK--EVL
54   APV--D--ESRAS--E--YGLVNID--RS---GRVVHFSEK--PTG---IDLKS--MQTDTTMHGLSHQE-AAKSP--YIASM--G---VYCFKT--EAL
55   APV--S--ESRAS--N--FGLVKID--RG---GRVIHFSEK--PTG---VDLKS--MQTDTTMLGLSHQE-ATDSP--YIASM--G---VYCFKT--EAL
56   APA--E--DSRAS--D--FGLVKID--SR---GRVVQFAEK--PKG---FDLKA--MQVDTTLVGLSPQD-AKKSP--YIASM--G---VYVFKT--DVL
57   VPM--D--DGRAS--D--FGLMKID--ET---GRIIQFAEK--PKG---PALKV--MQVDTSILGLSEQE-ASNFP--YIASM--G---VYVFKT--DVL
58   LPI--D--DSRAS--D--FGLMKID--DT---GRVMSFSEK--PKG---DDLKA--MAVDTTVLGLSPEE-AKEKP--YIASM--G---VYVFKK--DIL
59   APA--E--DSRAS--D--FGLVKID--SR---GRVVQFAEN--QR----FELKA--MLVDTSLVGLSPQD-AKKSP--YIASM--G---VYVFKT--DVL
60   VPM--D--DGRAS--D--FGLMKID--ET---GRIIQFVEK--PKG---PALKA--MQVDTSILGLSEQE-ASNFP--YIASM--G---VYVFKT--DVL
61   IPI--D--DRRAS--D--FGLMKID--DK---GRVISFSEK--PRG---DELKA--MAVDTTILGLSKEE-AEKKP--YIASM--G---VYVFKK--EIL
62   LPM--D--ESRAS--D--FGLLKID--QS---GKIIQFSEK--PKG---DDLKA--MQVDTSILGLPPKE-AAESP--YIASM--G---VYVFRK--EVL
63   APV--D--ESRAS--D--YGLVNID--RS---GRVVHFSEK--PTG---IDLKS--MQTDTTMHGLSHQE-AAKSP--YIASM--G---VYCFKT--EAL
64   APV--S--ESRAS--N--FGLVKID--RG---GRVIHFSEK--PTG---VDLKS--MQTDTTMLGLSHQE-ATDSP--YIASM--G---VYCFKT--EAL
66   LPV--D--GSRAS--D--FGLVKVD--ER---GQICQFLEK--PKG---ELLRS--MHVDTSIFGLSAQE-ARKFP--YIASM--G---IYVFKI--DVL
67   LPM--D--DSRAS--D--FGLMRID--NK---GRILSFSEK--PKG---EELKA--MQVDTTVLGLSKDE-AQKKP--YIASM--G---VYVFKK--EIL
```

```
68  VPM--D--ESRAS--D--YELMKID--RK---GQITQFVEK--PEG---SDLQA--MHVDTTLLGLTAEE-AQTYP--YIAPM--G---VSVFRT--ETL
69  VPM--D--ESRAS--D--YELMKID--RK---GEITQFVEK--PEG---SDLKA--MHVDTTLLGLTAEE-AQTYP--YIAPM--G---VSVFRT--ETL
70  AAV--G--ESRAS--D--YGLVKAD--GR---GRIIQFSEK--PKG---ADLKA--MQVDTSVLGLPPHE-AKRSP--YIASM--G---VYVFKT--DVL
71  AAV--G--ESRAS--D--YGLVKAD--AR---GRIIQFSEK--PNG---ADLKA--MQVDTSVLGLPLHE-AKRSP--YIASM--G---VYVFKT--DVL
72  LPM--D--DSRAS--D--FGLMKID--NK---GRVLSFSEK--PKG---EELKA--MQVDTTVLGLSKDE-AQKKP--YIASM--G---VYVFKK--EIL
73  VPM--D--DSRAS--D--YGLMKID--KT---GRIIQFAEK--PKG---SDLKA--MRVDTTLLGLLPQE-AEKHP--YIASM--G---VYVFRT--ETL
74  AAV--G--DSRAS--D--YGLVKVD--DR---GRIIQFSEK--PKG---DDLKA--MQADTSLLGLSSQD-ALESP--YIASM--G---VYVFKT--DVL
75  VPM--D--DSRAS--D--YGLMKID--KT---GRIIQFAEK--PKG---SDLKA--MRVDTTLLGLSPQE-AEKYP--YIASM--G---VYVFRT--ETL
76  AAV--G--DSRAS--D--YGLVKVD--DR---GRIIQFSEK--PNG---DDLKA--MQADTSLLGLSPQD-ALKSP--YIASM--G---VYVFKT--DVL
77  LPV--D--GSRAS--D--FGLVKVD--ER---GQIRQFLEK--PKG---ELLRS--MHVDTSIFGLSAQE-ARKFP--YIASM--G---IYVFKI--DVL
80  AAV--G--DSRAS--D--YGLVKVD--SG---GRIIQFSEK--PKG---ADLKS--MQADTSLFGLSNQD-ALRSP--YIASM--G---VYVFKT--DVL
81  LPV--N--DSRAS--D--FGLVKID--ET---GQIRQFLEK--PKG---ENLKS--MKVDTTVLGLSAQE-ANKFP--YIASM--G---IYMFKT--DVL
82  AAV--G--ESRAS--D--YGLVKID--GR---GQVFQFAEK--PKG---SELRE--MRVDTTRLGLSPQD-AMKSP--YIASM--G---VYVFKT--DIL
84  LPM--D--DSRAS--D--FGLMKID--NK---GRVLSFSEK--PKG---VDLKA--MEVDTTVLGLSKEE-ALKKP--YIASM--G---VYVFKK--EIL
87  LPM--D--DSRAS--D--FGLMNID--NK---GRVLSFSEK--PKG---ADLKA--MAVDTTVLGLSKEE-AEKKP--YIASM--G---VYVFKK--EIL
88  LPV--D--ESRAS--D--FGLIKID--ET---GQIRQFLEK--PKG---ESLKS--MRVDTSTLGLSISD-ARKLP--YIASM--G---IYMFKT--DVL
89  AAV--G--ESRAS--D--YGLVKID--SR---GRIVHFAEK--PGG---AELKS--LKADTTQLGLSPQD-ALKSP--YIASM--G---VYVFRT--EIL
90  VPM--D--DSRAS--D--YGLMKID--NT---GRIIQFAEK--PKG---LDLKA--MQIDTKLLGLSKQD-ALQYP--YIASM--G---VYVFRT--EVL
91  VPM--D--DSRAS--D--YGLMKID--NT---GRIIQFSEK--PKG---PNLKA--MKVNTTLLGLSEKE-AEKCP--YIASM--G---VYVFRT--DVL
92  LPM--D--ESRAS--D--FGLIKID--EM---GQIRQFLEK--PKG---ETLKS--MRVDTTALGLSPVE-AKKFP--YIASM--G---IYLFKT--DVL
94  LPM--D--DSRAS--D--FGLMKID--NK---GRVLFFSEK--PKG---EDLKA--MEVDTKVLGLSREE-AEKKP--YIASM--G---VYVFKK--EIL
95  VPV--G--ESRAS--D--YGLLKMD--NR---GRIIQFAEK--PKG---ADLKA--MKVDTTRLGLSPQE-AMKSP--YIASM--G---VYVFKT--DIL
96  VPM--D--DSRAS--D--YGLMKID--NT---GRIVQFAEK--PKG---PDLKA--MQVDTTLLGLSRQE-AMQFP--YIASM--G---VYVFRT--DVL
97  VAV--G--ESRAS--D--YGLVKID--SK---GQIFQFTEK--PKG---SELRE--MQVDTTHLGLSPQD-ALKSS--YIASM--G---VYVFKT--DIL
98  VPM--D--DSRAS--D--YGLMKID--ST---GRIIQFAEK--PKG---TDLKA--MQVDTTLLGLSKQE-AMQFP--YIASM--G---VYVFRT--DVL
99  LPI--D--GSRAS--D--FGLMKID--DT---GRVISFSEK--PKG---DELKA--MQVDTTVLGLSKEE-AENKP--YIASM--G---IYIFKK--DIL
100 APV--D--ESRAS--K--NGLVKID--HT---GRVLQFFEK--PKG---ADLNS--MRVETNFLSYAIDD-AQKYP--YLASM--G---IYVFKK--DAL
101 APV--G--ESRAS--D--YGLVKFD--SS---GRVIQFSEK--PKG---AALEE--MKVDTSFLNFATCTLPAEYP--YIASM--G---VYVFKR--DVL
102 VPM--D--ESRAS--D--FGLMKAD--RN---GHITDFLEK--PKG---ADLES--MQVDMGLFGLSPEF-ASTYK--YMASM--G---IYVFKA--DVL
103 APV--G--ESRAS--E--YGLVKFD--SS---GRVIQFSEK--PKG---VDLEA--MKVDTSFLNFAIDD-PAKFP--YIASM--G---VYVFKR--DVL
104 VPM--D--ESRAS--D--FGLMKTD--RN---GRITDFLEK--PKG---ESLKS--MVVDMEIFGLSPEV-ANVYN--YLASM--G---IYVFKT--DVL
105 LPI--D--DSRAS--D--FGLMKID--DT---GRVISFSEK--PKG---DDLKA--MQVDTTVLGLSKEE-AEEKP--YIASM--G---VYIFKK--EIL
106 LPI--D--DSRAS--D--FGLMKID--DT---GRVIAFSEK--PKG---DDLKA--MQVDTTVLGLPQDE-AKEKP--YIASM--G---VYIFKK--EIL
107 APV--G--ESRAS--D--YGLVKFD--SS---GRVIQFSEK--PKG---TDLEA--MKVDTSFLNFAIDD-PTKFP--YIASM--G---VYVFKR--DVL
108 VPV--D--ESRAS--D--FGLMKTD--KN---GRITDFLEK--PKD---ESLKS--MQLDMGTFGLRPEV-ADTCK--YMASM--G---IYVFRT--DIL
109 API--D--GSRAS--D--YGLVKFD--DS---GRVIQFLEK--PEG---ADLES--MKVDTSFLSYAIDD-KQKYP--YIASM--G---IYVLKK--DVL
110 APV--G--ESRAS--D--YGLVKFD--SS---GRVIQFSEK--PKG---AALEE--MKVDTSFLNFAIDD-PTKYP--YIASM--G---VYVFKR--DVL
111 APV--D--ESRAS--N--NGLVKCD--HT---GRVLQFFEK--PKG---ADLNS--MRVDTNFLSYAIGD-AQKYQ--YIASM--G---IYVFKK--DAL
```

```
112  LPI--D--DSRAS--D--FGLMKID--DT---ARVISFSEK--PKG---DELKA--MQVDTTVLGLSKEE-AEKKP--YIASM--G---VYIFKK--DIL
113  APV--G--ESRAS--E--YGLVKFD--SS---GRVIQFSEK--PKG---DDLEA--MKVDTSFLNFAIDD-PAKYP--YIASM--G---VYVFKR--DVL
114  LPI--D--DSRAS--D--FGLMKID--DT---GRVISFSEK--PKG---ADLKA--MQVDTTLLGLPKEE-AEKKP--YIASM--G---VYIFKK--EIL
115  APV--G--ESRAS--E--YGLVKFD--SS---GRVIQFSEK--PKG---DDLEA--MKVDTSFLNFAIDD-PAKYP--YIASM--G---VYVFKR--DVL
116  VPM--D--VSRAS--D--FGLMKID--NN---GRVLSFSEK--PKG---QELKA--MEVDTSVLGLSREQ-AKKTP--FIASM--G---VYVFKK--EIL
117  APV--G--ESRAS--E--YGLVKFD--SS---GRVVQFSEK--PKG---DDLEA--MKVDTSFLNFAIDD-PAKYP--YIASM--G---VYVFKR--DVL
118  LPI--D--GSRAS--D--FGLMKID--DT---GRVISFSEK--PRG---ADLKA--MQVDTTLLGLPKEE-AEKKP--YIASM--G---VYIFKK--EIL
119  APV--G--ESRAS--E--YGLVKFD--SS---GRVVQFSEK--PKG---DDLEA--MKVDTSFLNFAIDD-PAKYP--YIASM--G---VYVFKR--DVL
120  APV--G--ESRAS--E--YGLVKFD--SS---GRVVQFSEQ--PKG---DDLEA--MKVDTSFLNFAIDD-PAKYP--YIASM--G---VYVFKR--DVL
121  LPV--D--PQQAE--A--FGLMRTD--AH---GTIQEFREK--PKG---DSLKE--MAVDTSRFGLSPES-AEQKP--YLASM--G---IYVFSR--KAL
122  LPV--D--EKRAS--S--FGLMKID--EST--GRIIDFSEK--PKG---EALKQ--MAVDTSTLGLSPEE-AAESP--YIASM--G---IYVFKK--DVL
123  IPI--D--KSRAS--D--FGLMKID--QS---GRVIDFSEK--PKG---DELDR--MQVDTSVLGLSPEQ-AKLQP--YIASM--G---IYVFKK--DVL
124  VPI--D--EKRAS--S--FGLMKIN--DN---GRIVDFAEK--PKG---EELKQ--MQVDTSILGLNPEQ-AKESP--YIASM--G---IYVFNK--KAL
125  LPV--D--EKQAS--S--FGLMKID--DT---GRIIDFQEK--PKG---DDLKR--MQVDTTTLGLSAEE-SKIKP--YIASM--G---IYLFKR--EVL
126  LPV--D--AAQAE--G--FGLMHTN--EH---GRIREFREK--PKG---EALKE--MWVDTSKLGLSADE-ALKRP--YLASM--G---IYVFSR--ETL
127  LPV--D--APQAE--A--FGLMHTD--EK---GKIREFREK--PKG---DALKE--MWVDTSRLGLSPEE-AEKRP--YLASM--G---IYVFSR--ETL
128  LPV--D--AEQAE--G--FGLMRTD--LD---GRIREFSEK--PKG---AALEA--MKVDTARLGLAEAE-ATRRP--YLASM--G---IYVFSR--DTL
129  LPI--D--EKRAS--D--FGLMKIN--DT---GRIIDFSEK--PKG---DALKK--MAVDTTTLGLSAEE-AKESP--YIASM--G---IYVFNR--EVL
130  LPV--D--PQQAE--A--FGLMRTD--EH---GTIQEFREK--PKG---DSLKE--MAVDTSRFGLSPES-AQSKP--YLASM--G---IYVFSR--KAL
131  LPV--D--AKQAE--S--FGLMRTD--SE---GNIQEFREK--PKG---DSLLE--MAVDTSRFGLSPES-AQERP--YLASM--G---IYVFSR--ETL
132  LPV--D--AKQAE--A--FGLMRTD--QD---GRILEFREK--PKG---DSLLE--MAVDTSRFGLSADS-AKERP--YLASM--G---IYVFSR--DTL
133  LPC--D--LERAS--D--FGLIKTD--AD---GRVVQFTEK--PKG---AELER--MRVDTTTLGLTLEE-AERRP--FVASM--G---IYVFRH--DVM
134  LPV--D--ESQAE--G--FGLMRTD--DL---GNIKEFSEK--PTG---EKLKS--MAVDTSKFGLTKES-ASEKP--YLASM--G---IYVFSR--KTL
135  LPV--D--GAQAE--G--FGLMRTD--ND---GNIREFKEK--PSG---EALKA--MAVDTSRFGLSPDS-AKERP--YLASM--G---IYVFSR--STL
136  LPV--E--EKAAS--G--FGLLKVD--GT---GRVTDFREK--PTG---DALRD--MRVDTTRYGLTIEE-AHRKP--YIASM--G---IYVFKR--QVL
137  VPI--D--EERAS--S--FGLMKID--DH---GRVVDFSEK--PKG---DELKQ--MQVDTTVLGLTPEQ-AKESP--YIASM--G---IYVFKK--EVL
138  IPI--D--ERRAS--D--FGLMKID--DS---GRIIDFSEK--PKG---EALTQ--MQVDTSVLGLTKEQ-AQKQP--YIASM--G---IYVFKK--EVL
139  LPV--D--AEQAE--G--FGLMRTD--SD---GNIQEFREK--PKG---ESLKA--MAVDTSRFGLSAES-AKNKP--YLASM--G---IYVFSR--ATL
140  LPC--E--EKVAS--G--FGLLKLG--EN---GRIVDFKEK--PTG---DLLKA--CQVDTQALGLSPEE-AKAKP--YIASM--G---IYVFKR--EAL
141  LPC--E--EKVAS--G--FGLLKID--AD---GRIVDFKEK--PQG---ELLKA--CQVDTQALGLSPEE-AKAKP--YIASM--G---IYVFRR--EAL
142  LPV--D--AEQAE--G--FGLMRTD--SD---GNIQEFREK--PKG---ESLKA--MAVDTSRFGLSAES-ARNKP--YLASM--G---IYVFSR--ATL
143  LPV--D--PKQAE--A--FGLMRTD--GD---GDIKEFREK--PKG---DSLLE--MAVDTSRFGLSANS-AKERP--YLASM--G---IYVFSR--DTL
144  IPI--D--DRRAS--D--FGLMKID--NS---GRVIDFSEK--PKG---EALTK--MRVDTTVLGLTPEQ-AASQP--YIASM--G---IYVFKK--DVL
145  LPV--D--SSQAE--A--FGLMRTD--GE---GNIKEFREK--PTG---DSLKA--MAVDTSRFGLTAQS-AKERP--YLASM--G---IYVFSR--ATL
146  LPV--D--EAQAE--G--FGLMRTD--DL---GNIKEFSEK--PTG---EKLKA--MAVDTSKFGLSKES-AAEKP--YLASM--G---IYVFSR--NTL
147  LPV--D--SAQAE--A--FGLMRTD--EA---GNIKEFREK--PTG---DSLKA--MAVDTSRFGLEANE-AKEKP--YLASM--G---IYVFSR--STL
148  LPV--D--EAQAE--G--FGLMRTD--DV---GNIKEFSEK--PSG---EKLKA--MAVDTSKFGLSKES-AAEKP--YLASM--G---IYVFSR--NTL
149  LPV--D--EAQAE--G--FGLMRTD--DL---GNIKEFSEK--PTG---EKLKS--MAVDTSKFGLTKES-ALEKP--YLASM--G---IYVFSR--KTL
```

```
150  LPV--D--AAQAE--A--FGLMRTD--EV---GNIKEFREK--PKG---DSLKA--MAVDTSRFGLSVES-SKERP--YLASM--G---IYVFSR--KTL
151  VPI--D--EKRAS--S--FGLMKID--DN---ARVVDFSEK--PKG---EALRQ--MQVDTSILGLSPDQ-ARKNP--YIASM--G---IYIFNR--EVL
152  LPV--D--EAQAE--G--FGLMRTD--DL---GNIKEFSEK--PTG---EKLKA--MAVDTSKFGLTKES-AAEKP--YLASM--G---IYVFSR--NTL
153  VPV--D--DRKAP--E--LGLMKID--AQ---GRITDFSEK--PQG---EALRA--MQVDTSVLGLSAEK-AKLNP--YIASM--G---IYVFKK--EVL
154  VPI--D--EERAS--S--FGLMKID--DH---GRVVDFSEK--PKG---DELKQ--MQVDTTVLGLTPEQ-AKESP--YIASM--G---IYVFKK--EVL
155  LPI--D--EKRAS--D--FGLMKLD--GS---GRVVEFSEK--PKG---DELRA--MQVDTTILGLDPVA-AAAQP--FIASM--G---IYVFKR--DVL
156  LPV--E--EQVAS--S--FGLLQVD--HS---GRVTAFSEK--PQG---EALTR--MRVDTTDFGLTPAE-AAHKP--YLASM--G---IYVFNR--QVL
157  LPI--D--EKRAS--D--FGLMKLD--GS---GRVVEFSEK--PKG---DELRA--MQVDTTILGLDPVA-AAAQP--FIASM--G---IYVFKR--DVL
158  LPV--D--PKQAE--A--FGLMRTD--EN---GSIKEFREK--PKG---DSLLE--MSVDTSRFGLSVES-AKERP--YLASM--G---IYVFSR--QTL
159  LPV--D--AKQAE--S--FGLMRTD--SD---GNIQEFREK--PKG---DSLRE--MAVDTSRFGLTPES-AQERP--YLASM--G---IYVFSR--DTL
160  VPM--D--ERRAS--A--FGLMKLD--ES---GRVGDFCEK--PSG---DELTQ--MQVDTTLLGLSAEQ-AREQP--YIASM--G---IYVFKK--EVL
161  LPI--D--AYRAP--A--FGLMKID--ESS--GRVVDFSEK--PQG---EELER--MKVDTTTLGLTPEE-AQEKP--FIASM--G---IYVFKK--DVL
162  LPI--D--EARAS--E--FGVMKID--NS---GRIVEFSEK--PKG---NALKA--MAVDTSILGVSPEI-ATKQP--YIASM--G---IYVFNK--DAM
163  IPI--D--DRRAS--D--FGLMKID--NS---GRVIDFSEK--PKG---EALTK--MRVDTTVLGLTPEQ-AASQP--YIASM--G---IYVFKK--DVL
164  LPV--D--EKQAS--S--FGLMKID--NT---GRIIDFQEK--PKG---DDLKR--MEVDTKTLGLSAQE-AKMKP--YIASM--G---IYLFKR--EVL
165  VPI--D--EKRAS--S--FGLMKID--DN---GRIVDFSEK--PKG---EELKQ--MQVDTSILGLNPEQ-AKESP--YIASM--G---IYVFNK--KAL
166  VPV--G--EKVAP--A--FGLMKID--AN---GRVVDFSEK--PTG---EALKA--MQVDTQSLGLDPEQ-AKEKP--YIASM--G---IYVFKK--QVL
167  LPV--D--EGQAE--G--FGLMRTD--DL---GNIKEFSEK--PTG---KKLKA--MAVDTSKFGLSKYS-AAEKP--YLASM--G---IYVFSR--NTL
168  LPV--D--PAQAE--A--FGLMRTD--EI---GNIKEFREK--PTG---DSLKA--MAVDTSRFGLEANE-AKEKP--YLASM--G---IYVFSR--STL
169  LPV--D--REQAQ--S--FGLMHTG--AE---ASITKFREK--PKG---EALDE--MSCDTASMGLSAEE-AHRRP--FLASM--G---IYVFKR--DVL
170  LPV--D--AKQAE--A--FGLMRTD--ED---GRILEFREK--PKG---DSLLE--MAVDTSRFGLSAES-AKERP--YLASM--G---IYVFSR--DTL
171  VPI--D--ERRAS--A--FGVMKIN--DS---GRIVDFYEK--PKG---AELER--MRVDTTILGLSPDQ-ARQSP--YIASM--G---IYVFKK--NVL
172  LPV--D--EGQAE--G--FGLMRTD--DL---GNIKEFSEK--PTG---EKLKA--MAVDTSKFGLSKDS-AAKKP--YLASM--G---IYVFSR--NTL
173  LPV--D--PVQAE--A--FGLMRTD--GE---GHIQEFREK--PKG---EALEA--MRVDTQSLGLSPEE-AAKRP--HLASM--G---IYVFSR--DTL
174  VPI--D--EKRAS--S--FGLMKID--DN---GRVIDFSEK--PKG---DALKQ--MQVDTTILGLSPDQ-ARKSP--YIASM--G---IYVFKK--DVL
175  IPI--D--DYRAS--D--FGLMKID--NS---GRVIDFSEK--PKG---EALAQ--MRVDTTVLGLTKEQ-AELQP--YIASM--G---IYVFKK--DVL
176  VPI--D--ETRAS--S--FGLMQIN--DR---GKVIDFREK--PTG---ELLKQ--MQVDTTVLGLTPEE-ARNSP--YIASM--G---IYVFSK--AVM
177  IPI--D--ERRAS--D--FGLMKIN--ES---GRVVDFSEK--PKG---EALKK--MRVDTTVLGLNQEQ-AEQQP--YIASM--G---IYVFKK--DVL
178  VPI--D--EKRAS--S--FGLMKID--DN---GRIVDFSEK--PKG---EELKQ--MQVDTSILGLNPEQ-AKESP--YIASM--G---IYVFNK--KAL
179  LPV--D--EKQAS--S--FGLMKID--NT---GRIIDFQEK--PKG---DDLKR--MEVDTKTLGLSAQE-AKMKP--YIASM--G---IYLFKR--EVL
180  LPV--D--AAQAE--A--FGLMRTD--ND---GNIKEFREK--PKG---DSLKE--MAVDTSRFGLSAES-SKERP--YLASM--G---IYVFSR--KTL
181  VPV--N--KTRAS--C--LGLTKIN--NQ---GKVIRFFEK--PSE---NELNQ--MQCKSSILGLSKEQ-AIKKP--YMASM--G---IYVFNK--KVL
182  LPM--D--EKRAS--D--FGLMKID--DK---GRIVSFSEK--PKG---DALKQ--MQVDTTTLGLTPQQ-AQESP--YIASM--G---IYVFKK--EVL
183  LPM--D--DKRAS--D--FGLMKTD--ED---GRIVSFSEK--PKG---EALKE--MQVDTTKLGLTAEQ-AKESP--YIASM--G---IYVFNK--DVL
184  IPI--D--GRRAS--D--FGLMKID--SA---GRVIDFSEK--PKG---EALAK--MQVDTTVLGLTSEE-ARSQP--YIASM--G---IYVFKK--DVL
185  IPI--D--GRRAS--D--FGLMKID--NG---GRVIDFSEK--PKG---EALAK--MQVDTTILGLTSEE-ARSQP--YIASM--G---IYVFKK--DVL
186  LPV--D--EKRAS--A--FGLMKID--EST--GRIIDFSEK--PKG---EALKQ--MAVDTSSLGLSPEE-AAESP--YIASM--G---IYVFKK--DVL
187  VPI--D--ERRAS--S--FGLMKID--DS---GRVVDFSEK--PKG---DALKQ--MQVDTSILGLNPEQ-AKESP--YIASM--G---IYVFNK--KAL
```

```
188   LPV--D--PKQAE--A--FGLMRTD--EN---GSIKEFREK--PKG---DSLLE--MAVDTSRFGLSADS-AKERP--YLASM--G---IYVFSR--KTL
189   LPM--D--GERAS--D--FGLMKID--KT---GRITEFAEK--PEG---NDLLA--MQVDTTVLGLSPEE-SQASP--YIASM--G---IYVFKK--SAL
190   VPM--D--AARAE--A--FGLMKID--DS---GRIIDFAEK--PKG---KELEA--MAVDTTILGLDKKL-AKEMP--YIASM--G---IYVFKA--SAM
191   LPM--D--DKRAS--D--FGLMKID--DT---GRITEFAEK--PNG---DALKA--MEVDTTILGLTAEE-ATSSP--YIASM--G---IYVFKK--SAL
192   VPM--D--EERAA--A--FGLMKID--DT---GKIIDFAEK--PTG---DALKA--MMVDTTILGLDAER-AKEMP--YIASM--G---IYVFNA--RAM
193   IAY--G--SDRAK--E--FGLMKID--EK---RRVTSFAEK--PKTQ--EALDA--MKVDTTVLGLTPEE-AAEKP--YIASM--G---IYVFKK--SVL
194   LPC--A--EKEAS--A--FGLMKID--EE---GRVIEFAEK--PKG---EALTK--MRVDTGILGVDPAT-AAAKP--YIASM--G---IYVMSA--KAL
195   LPI--D--EARAS--D--FGLMKID--ST---GRIVEFTEK--PKG---DALQA--MKVDTTVLGLTADE-AKEKP--FIASM--G---IYVFKK--SAL
196   LPC--D--EKRAS--S--FGLMKID--NT---GRVIEFAEK--PKG---AELQA--MKVDTTVLGLDADK-AQEMP--FIASM--G---IYVFDA--KKM
197   LPC--A--EKEAS--A--FGLMKID--DA---GRVVEFAEK--PKG---EALQR--MKVDTSILGVDPAT-AQSKP--FIASM--G---IYVMSA--KAL
198   IAY--G--SDRAK--E--FGLMKID--DK---RRVLSFAEK--PKTQ--EALDA--MKVDTTVLGLTPDE-AADKP--YIASM--G---IYVFKK--SVL
199   LPI--D--EERAS--D--FGLMKID--SS---GRIVEFTEK--PKG---DALQA--MKVDTTILGLTAAE-AEAKP--FIASM--G---IYVFKK--SML
200   LPT--D--EKRAS--S--FGLMKIN--EH---ATIIEFSEK--PKG---DALKA--MQCDTTILGLDAER-AKEMP--YIASM--G---IYVFNA--KAM

              310       320       330       340       350       360       370       380       390       400
      ....|....|....|....|....|....|....|....|....|....|....|....|....|....|....|....|....|....|....|....|
1     LNLLRD--KFPGANDFGSE--VIPGATS---LGM----RVQAYLY---DGYW--EDI--GT--IEAFYNANLGI--TKKPVPDFSFYDRSAPIYTQPRYL
2     LNLLRD--KFPGANDFGSE--VIPGATS---LGM----RVQAYLY---DGYW--EDI--GT--IEAFYNANLGI--TKKPVPDFSFYDRSAPIYTQPRYL
3     LNLLRD--KFPGANDFGSE--VIPGATS---LGM----RVQAYLY---DGYW--EDI--GT--IEAFYNANLGI--TKKPVPDFSFYDRSAPIYTQPRYL
4     LNLLRD--KFPGANDFGSE--VIPGATS---LGM----RVQAYLY---DGYW--EDI--GT--IEAFYNANLGI--TKKPVPDFSFYDRSAPIYTQPRYL
5     LDLLRN--QFPGANDFGSE--VIPGATS---LGL----RVQAYLY---DGYW--EDI--GT--IEAFYNANLGI--TKKPVPDFSFYDRSAPIYTQPRYL
6     LELLRD--KFPGANDFGSE--VIPGATS---LGL----RVQAYLY---DGYW--EDI--GT--IEAFYNANLGI--TKKPVPDFSFYDRSAPIYTQPRYL
7     LDLLRD--QFPGANDFGSE--VIPGATS---IGK----RVQAYLY---DGYW--EDI--GT--IEAFYNANLGI--TKKPVPDFSFYDRSSPIYTQPRYL
8     LDLLRD--KFPGANDFGSE--VIPGATE---LGM----RVQAYLY---DGYW--EDI--GT--IEAFYNANLGI--TKKPVPDFSFYDRSSPIYTQPRYL
9     LDLLRE--KFPGANDFGSE--VIPEYVR--HVCVYS--QVQAYLY---DGYW--EDI--GT--IEAFYNANLGI--TKKPVPDFSFYDRSSPIYTQPRYL
10    LDLLRE--KFPGANDFGSE--VIPGATS---IGMR---NVQAYLY---DGYW--EDI--GT--IEAFYNANLGI--TKKPVPDFSFYDRSSPIYTQPRYL
11    LDLLRE--KFPGANDFGSE--VIPGATS---IGM----RVQAYLY---DGYW--EDI--GT--IEAFYNANLGI--TKKPVPDFSFYDRSSPIYTQPRYL
12    LDLLRD--KFPGANDFGSE--VIPGATS---IGL----RVQAYLY---DGYW--EDI--GT--IEAFYNANLGI--TKKPIPDFSFYDRSSPIYTQPRYL
13    LDLLRD--QFPGANDFGSE--VIPGATS---LGL----RVQAYLY---DGYW--EDI--GT--IEAFYNANLGI--TKKPVPDFSFYDRSSPIYTQPRYL
14    LDLLRD--QFPGANDFGSE--VIPGATD---LGL----RVQAYLY---DGYW--EDI--GT--IEAFYNANLGI--TKKPVPDFSFYGRSAPIYTQPRYL
16    LDLLRD--KFPGANDFGSE--VIPGATE---LGL----RVQAYLY---DGYW--EDI--GT--IEAFYNANLGI--TKKPVPDFSFYDRSSPIYTQPRYL
17    LDLLRD--KFPGANDFGSE--VIPGATS---VGM----RVQAYLY---DGYW--EDI--GT--IEAFYNANLGI--TKKPVPDFSFYDRSSPIYTQPRYL
18    LDLLRE--KFPGANDFGSE--VIPGATN---IGM----RVQAYLY---DGYW--EDI--GT--IEAFYNANLGI--TKKPVPDFSFYDRSSPIYTQPRYL
19    LDLLSD--KFPGANDFGSE--VIPGATS---IGM----RVQAYLY---DGYW--EDI--GT--IEAFYNANLGI--TKKPVPDFSFYDRSSPIYTQPRYL
20    LSLLRD--KFPGANDFGSE--VIPGATS---IGM----RVQAYLY---DGYW--EDI--GT--IEAFYNANLGI--TKKPVPDFSFYDRSSPIYTQPRYL
21    LNLLRD--KFPGANDFGSE--VIPGATS---IGM----RVQAYLY---DGYW--EDI--GT--IEAFYNANLGI--TKKPVPDFTFYDRSSPIYTQPRYL
22    LSLLRD--KFPGANDFGSE--VIPGATS---IGM----RVQAYLY---DGYW--EDI--GT--IEAFYNANLGI--TKKPIPDFSFYDRSDPIYTQPRYL
23    LNLLRD--KFPGANDFGSE--VIPGATS---IGM----RVQAYLY---DGYW--EDI--GT--IEAFYNANLGI--TKKPIPDFSFYDRSAPIYTQPRYL
```

24 LDLLRE--KFPGANDFGSE--VIPGATS---IGL----RVQAYLY---DGYW--EDI--GT--IEAFYNANLGI--TKKPIPDFSFYDSSSPIYTQPRYL
25 VNLLRQ--KFPGANDFGSE--VIPGATS---IGL----RVQAYLF---DGYW--EDI--GT--IEAFYNANLGI--TKKPVPDFSFYDRSAPIYTQPRYL
26 LNLLRE--KFPGANDFGSE--VIPGATS---IGM----RVQAYLF---DGYW--EDI--GT--IEAFYNANLGI--TKKPVPDFSLYDRSAPIYTQPRYL
27 LNLLRD--KFPGANDFGSE--VIPGATS---VGL----RVQAYLY---DGYW--EDI--GT--IEAFYNANLGI--TKKPVPDFSFYDRSAPINTQPRYL
28 LNLLRD--EFPAANDFGSE--VIPGATA---MGL----RVQAYLF---DGYW--EDI--GT--IEAFYNANLGI--TKKPVPDFSFYDRSAPIYTQPRYL
29 LNLLRE--KFPAANDFGSE--VIPGATS---IGL----RVQAYLY---DGYW--EDI--GT--IEAFYNANLGI--TKKPVPDFSFYGRSSPIYTQPRYL
30 LDLLRD--KFPGANDFGSE--VIPGATE---LGM----RVQAYLY---DGYW--EDI--GT--IEAFYNANLGI--TKKPVPDFSFYDRSSPIYTQPRYL
31 LDLLRD--KFPGANDFGSE--VIPGATS---IGM----RVQAYLY---DGYW--EDI--GT--IEAFYNANLGI--TKKPVPDFSFYDRSSPIYTQPRYL
32 LRLLRE--NFPAANDFGSE--VIPGATE---IGL----RVQAYLY---DGYW--EDI--GT--IEAFYNANLGI--TKKPVPDFSFYDRSAPIYTQPRYL
33 LQLLRE--QFPEANDFGSE--VIPGATS---IGK----RVQAYLY---DGYW--EDI--GT--IAAFYNANLGI--TKKPIPDFSFYDRFAPIYTQPRHL
34 LQLLRE--QFPEANDFGSE--VIPGATS---IGK----RVQAYLY---DGYW--EDI--GT--IAAFYNANLGI--TKKPMPDFSFYDRFAPIYTQPRHL
36 LQLLRE--QFPGANDFGSE--VIPGATS---IGK----RVQAYLY---DGYW--EDI--GT--IEAFYNANLGI--TKKPIPDFSFYDRSAPIYTQPRHL
37 LQLLRE--QFPGANDFGSE--VIPGATN---IGM----RVQAYLY---DGYW--EDI--GT--IEAFYNANLGI--TKKPVPDFSFYDRSAPIYTQPRHL
38 LKLLRQ--NFPAANDFGSE--VIPGATE---IGM----RVQAYLY---DGYW--EDI--GT--IEAFYNANLGI--TKKPVPDFSFYDRSAAIYTQPRYL
39 LQLLRE--QFPGANDFGSE--VIPGATN---IGM----RVQAYLY---DGYW--EDI--GT--IEAFYNANLGI--TKKPVPDFSFYDRSAPIYTQPRHL
41 LQLLRE--QFPGANDFGSE--VIPGATS---TGM----RVQAYLY---DGYW--EDI--GT--IEAFYNANLGI--TKKPIPDFSFYDRSAPIYTQPRHL
42 LQLLRE--QFPGANDFGSE--VIPGATS---TGM----RVQAYLY---DGYW--EDI--GT--IEAFYNANLGI--TKKPIPDFSFYDRSAPIYTQPRHL
43 LRLLRE--NFPAANDFGSE--VIPGATE---IGL----RVQAYLY---DGYW--EDI--GT--IEAFYNANLGI--TKKPVPDFSFYDRSAPIYTQPRYL
44 LRLLRD--NFPSANDFGSE--VIPGATE---IGM----RVQAYLY---DGYW--EDI--GT--IEAFYNANLGI--TKKPVPDFSFYDRSAPIYTQSRYL
45 LQLLRE--QFPGANDFGSE--VIPGATS---TGM----RVQAYLY---DGYW--EDI--GT--IEAFYNANLGI--TKKPIPDFSFYDRSAPIYTQPRHL
46 LQLLRE--QFPGANDFGSE--VIPGATS---TGM----RVQAYLY---DGYW--EDI--GT--IEAFYNANLGI--TKKPIPDFSFYDRSAPIYTQPRHL
47 LRLLRD--NFPSANDFGSE--VIPGATE---IGM----RVQAYLY---DGYW--EDI--GT--IEAFYNANLGI--TKKPVPDFSFYDRSAPIYTQSRYL
48 LQLLRE--QFPGANDFGSE--VIPGATS---TGM----RVQAYLY---DGYW--EDI--GT--IEAFYNANLGI--TKKPIPDFSFYDRSAPIYTQPRHL
49 LKLLKW--SYPTSNDFGSE--IIPAAID---D-Y----NVQAYIF---KDYW--EDI--GT--IKSFYNASLAL--TQEF-PEFQFYDPKTPFYTSPRFL
50 LNLLKS--AYPSCNDFGSE--IIPSAVK---D-H----NVQAYLF---NDYW--EDI--GT--VKSFFDANLAL--TKQP-PKFDFNDPKTPFYTSARFL
51 LNLLRW--RFPTANDFGSE--IIPASTK---E-F----CVKAYLF---NDYW--EDI--GT--IRSFFRANLAL--TEHP-PRFSFYDATKPIYTSRRNL
52 LNLLRW--RFPTANDFGSE--IIPFSAK---E-F----YVNAYLF---NDYW--EDI--GT--IRSFFEANLAL--TEHP-GAFSFYDAAKPIYTSRRNL
53 LKLLRS--SYPTSNDFGSE--IIPLAVG---E-H----NVQAFLF---NDYW--EDI--GT--IGSFFDANLAL--TEQP-PKFQFYDQKTPFFTSPRFL
54 LKLLTW--RYPSSNDFGSE--IIPAAIK---D-H----NVQGYIY---RDYW--EDI--GT--IKSFYEANIAL--VEEH-PKFEFYDQNTPFYTSPRFL
55 LNLLTR--QYPSSNDFGSE--VIPAAIR---D-H----DVQGYIF---RDYW--EDI--GT--IKTFYEANLAL--VEER-PKFEFYDPETPFYTSPRFL
56 LKLLKW--SYPTSNDFGSE--IIPAAID---D-Y----NVQAYIF---KDYW--EDI--GT--IKSFYNASLAL--TQEF-PEFQFYDPKTPFYTSPRFL
57 LKLLKS--AYPSCNDFGSE--IIPSAVK---D-H----NVQAYLF---NDYW--EDI--GT--VKSFFDANLAL--TKQP-PKFDFNDPKTPFYTSARFL
58 LNLLRW--RFPTVNDFGSE--IIPASTK---E-F----CVKAYYL--FNDYW--EDI--GT--IRSFFEANLAL--TEHP-PRFSFYDATKPIYTSRRNL
59 LKLLKW--SYPTSNDFGSE--IIPAAID---D-Y----NVQAYIF---KDYW--EDI--GT--IKSFYNASLAL--TQEF-PEFQFYDPKTPFYTSPRFL
60 LNLLKS--AYPSCNDFGSE--IIPSAVK---D-H----NVQAYLF---NDYW--EDI--GT--VKSFFDANLAL--TKQP-PKFDFNDPKTPFYTSARFL
61 LNLLRW--RFPTANDFGSE--IIPFSAK---E-F----YVNAYLF---NDYW--EDI--GT--IRSFFEANLAL--TEHP-GAFSFYDAAKPIYTSRRNL
62 LKLLRS--SYPTSNDFGSE--IIPLAVR---E-H----NVQAFLF---NDYW--EDI--GT--IGSFFDANLAL--TEQP-PKFQFYDPKTPFFTSPRFL
63 LKLLTW--RYPSSNDFGSE--IIPAAIR---D-H----NVQGYIY---RDYW--EDI--GT--IKSFYEANLAL--VEEH-PKFEFYDQNTPFYTSPRFL

```
64   LNLLTR--QYPSSNDFGSE--VIPAAIR---D-H----DVQGYIF---RDYW--EDI--GT--IKTFYEANLAL--VEER-PKFEFYDPDTPFYTSPRFL
66   LKVLRG--CYPNANDFGSE--VIPMAAK---D-F----NVQACLF---NGYW--EDI--GT--IKSFFDANLAL--MDQR-PKFQLYDQSKPIFTCPRFL
67   LNLLRW--RFPTANDFGSE--VIPASAR---E-F----YMKAYLF---NDYW--EDI--GT--IRSFFEANLAL--TEHP-PRFSFYDAAKPMYTSRRNL
68   LKLLRW--SCPSCNDFGSE--IIPSALR---D-H----KVQAYMF---RDYW--KDI--GT--IKSFFEANLEL--TKQS-PNFEFYDQETPFFTSPRFL
69   LKLLRW--SCPSCNDFGSE--IIPSALR---D-H----KVQAYMF---RDYW--KDI--GT--IKSFFEANLEL--TKQS-PNFEFYDQESPFFTSPRFL
70   LKLLKW--RYPTSNDFGSE--IIPAAVR---E-N----NVQAYFF---NDYW--EDI--GT--IKSFYDANLAL--TEEN-PMFKFYDPKTPIYTSPRFL
71   LRLLKW--RYPTSNDFGSE--IIPAAVR---E-N----NVQAYFF---IDYW--EDI--GT--IKSFYDANLAL--TEEN-PMFKFYDPKTPIYTSPRFL
72   LNLLRW--RFPTANDFGSE--VIPASAR---E-F----YMKAYLF---NDYW--EDI--GT--IRSFFEANLAL--TEHP-PRFSFYDAAKPMYTSRRNL
73   LQLLRW--KCSSCNDFGSE--IIPSAVN---E-H----NVQAYLF---NDYW--EDI--GT--IKSFFDANLAL--TEQP-PKFEFYDPKTPFFTSPRFL
74   LNLLKW--RYPTSNDFGSE--IIPAAVR---D-H----NVQSYFF---GDYW--EDI--GT--IKSFYNANLAL--TEES-HKFEFYDPKIPIYTSPGFL
75   LQLLRW--NGSSCNDFGSE--IIPSAVN---E-H----NVQAYLF---NDYW--EDI--GT--IKSFFDANLAL--TEQP-PKFEFYDPKTPFFTSPRFL
76   LNLLKW--RYPTSNDFGSE--IIPAAVR---D-H----DVQSYFF---EDYW--EDI--GT--IKSFYDANLAL--TEES-HKFEFYDPKIPIYTSPGFL
77   RKVLRG--CYPNANDFGSE--VIPMAAK---D-F----NVQACLF---NGYW--EDI--GT--IKSFFDANLAL--MDQR-PKFQLYDQSKPIFTCPRFL
80   LKLLKW--RYPTSNDFGSE--IIPASVK---E-Y----NVQAFFF---GDYW--EDI--GT--IKSFYDANMAL--TEES-PMFKFYDPKTPIFTSPGFL
81   LKLLRW--NYPTANDFGSE--IIPMSTK---E-Y----NVQAYLF---NGYW--EDI--GT--IKSFFDANLAL--TDQP-PNFHFFDPLKPIFTSPRFL
82   LKLLRW--RYPTANDFGSE--IIPAAVM---E-H----NVQAYIF---KDYW--EDI--GT--IKSFYEANLAL--AEEP-PKFEFYDPKTPFYTSPRFS
84   LNLLRW--RFPTANDFGSE--IIPASAK---E-F----YMKAYLF---NDYW--EDI--GT--IRSFFAANLAL--TEHP-PRFSFYDAAKPMYTSRRNL
87   LNLLRW--RFPTANDFGSE--IIPASAK---E-F----FIKAYLF---NDYW--EDI--GT--IQSFFAANLAL--TEHP-PRFSFYDAAKPMYTSRRNL
88   LKLLRW--HYPTANDFGSE--IIPLSAK---D-Y----NVRAYLF---NDYW--EDI--GT--IKSFFDSNLAL--TDQP-PEFQFFDPLKPIFTSPRFL
89   LKLLRW--RFPTSNDFGSE--IIPAAVM---E-H----NIQSYNF---RDYW--EDI--GT--IKSFYEANLAL--TEEP-PTFEFYDPKTPFYTSPRFL
90   CKLLRW--SYPSCIDFGSE--VIPYAVK---D-H----NVQAYLF---NDYW--EDI--GT--IKSFFDANLAL--TEQP-PKFEFYDPKTPFFTSPRFL
91   LKLLTR--KYLSCNDFGSE--IIPLAVK---D-H----NVQAYLF---NDYW--EDI--GT--IKSFFDANLAL--TEQP-PKFEFYDPKTPFYTSPRFL
92   LKLLRW--SYPTANDFGSE--VIPMAAE---E-C----NVQAYLF---NGYW--EDI--GT--IKSFFDANLAL--TDQP-PKFHFYDPLKPIFTSPRFL
94   LNLLRW--RFPTANDFGSE--IIPASAK---E-F----FIKAYLF---NDYW--EDI--GT--IRSFFEANLAL--TAHP-PRFSFYDATKPMYTSRRNL
95   LNLLRW--RYPTSNDFGSE--IIPLAVM---E-H----NVEAFLF---RDYW--EDI--GT--IKTFYEANMGL--TEEF-PKFEFYNPKTPIFTSPRFL
96   LKLLRW--SYPSCNDFGSE--IIPSAVR---D-H----NVQAYLF---NDYW--EDI--GT--VKSFFDANLGL--TKQP-PKFEFYDPQTPFFTSPRFL
97   LKLLRW--RFPTSNDFGSE--IIPAAVM---E-H----NVQAYIF---KDYW--EDI--GT--IKSFYEANLAL--AEEP-PKFEFYDPKTPFYTSPGFL
98   LKLLRC--SYPSCNDFGSE--IIPSAVK---E-H----NVQAYLF---NDYW--EDI--GT--IKSLFDANLAL--TEQP-PKFEFYDPKTPFFTSPRFL
99   LNLLRW--RFPTANDFGSE--IIPASAK---E-I----DVKAYLF---NDYW--EDI--GT--IKSFFEANLAL--AEQP-PRFSFYDADKPMYTSRRNL
100  LDLLKS--KYTQLHDFGSE--ILPRAVL---D-H----SVQACIF---TGYW--EDV--GT--IKSFFDANLAL--TEQP-SKFDFYDPKTPFFTAPRCL
101  LDLLKS--RYAELHDFGSE--ILPKALH---E-H----NVQAYVF---TDYW--EDI--GT--IRSFFDANMAL--CEQP-PKFEFYDPKTPFFTSPRYL
102  RKLLRG--HYPTANDFGLE--VIPMAAK---D-Y----DVQAYLF---DGYW--EDI--GT--IKSFFEANLAL--TDQS-PNFYFYDPVKPIFTSPRFL
103  LNLLKS--RYAELHDFGSE--ILPRALH---E-H----NVQAYVF---TDYW--EDI--GT--IRSFFDANMAL--CEQP-PKFEFYDPKTPFFTSPRYL
104  LRLLRG--HYPTANDFGSE--VIPMAAK---D-Y----NVQAYLF---DGYW--EDI--GT--IKSFFEANLAL--TDQS-PNFHFYEPVKPIFTSPRFL
105  LNLLRW--RFPTANDFGSE--IIPAAAK---E-I----NVKAYLF---NDYW--EDI--GT--IKSFFEANLAL--AEQP-PRFSFYDASKPMYTSRRNL
106  LNLLRW--RFPTANDFGSE--IIPASAK---E-I----NVKAYLF---NDYW--EDI--GT--IKSFFEANLSL--AEQP-PRFSFYDANKPMYTSRRNL
107  LNLLKS--RYAELHDFGSE--ILPRALH---E-H----NVQAYVF---ADYW--EDI--GT--IRSFFDANMAL--CEQP-PKFEFYDPKTPFFTSPRYL
108  LRLLRG--HYPTANDFGSE--VIPMAAK---D-Y----NVQAYLF---DGYW--EDI--GT--IKSFFEANLAL--TDQS-PNFYFYDPVKPIFTSPRFL
```

```
109  LDILKS--KYAHLQDFGSE--ILPRAVL---E-H----NVKACVF---TEYW--EDI--GT--IKSFFDANLAL--TEQP-PKFEFYDPKTPFFTSPRYL
110  LDLLKS--RYAELHDFGSE--ILPKALH---E-H----NVQAYVF---TDYW--EDI--GT--IRSFFDANMAL--CEQP-PKFEFYDPKTPFFTSPRYL
111  LDLLKS--KYTQLHDFGSE--ILPRAVL---E-H----NVQTCIF---MGYW--EDV--GT--IKSFFDANLAL--TEQP-SKFDFYDPKTPFFTAPRYL
112  LNLLRW--RFPTANDFGSE--IIPAAAK---E-I----NVKAYLF---NDYW--EDI--GT--IKSFFEANLAL--AEQP-PRFSFYDADKPMYTSRRNL
113  LNLLKS--RYAELHDFGSE--ILPRALH---D-H----NVQAYVF---TDYW--EDI--GT--IRSFFDANMAL--CEQP-PKFEFYDPKTPFFTSPRYL
114  LNLLRW--RFPTANDFGSE--IIPAAAR---E-I----NVKAYLF---NDYW--EDI--GT--IKSFFEANLAL--AEQP-SKFSFYDASKPMYTSRRNL
115  LNLLKS--RYAELHDFGSE--ILPRALH---D-H----NVQAYVF---TDYW--EDI--GT--IRSFFDANMAL--CEQP-PKFEFYDPKTPFFTSPRYL
116  LNLLRW--RFPTANDFGSE--IIPASAK---E-L----FVKAYLF---NDYW--EDI--GT--IKSFCEANLSL--TRHP-PNFSFYDATKPIYTSRRNL
117  LNLLKS--RYAELHDFGSE--ILPRALH---D-H----NVQAYVF---TDYW--EDI--GQ--SDPSFDANMAL--CEQP-PKFEFYDPKTPFFTSPRYL
118  LNLLRW--RFPTANDFGSE--IIPAAAR---E-I----NVKAYLF---NDYW--EDI--GT--IKSFFEANLAL--AEQP-SKFSFYDASKPMYTSRRNL
119  LNLLKS--RYAELHDFGSE--ILPRALH---D-H----NVQAYVF---TDYW--EDI--GT--IRSFFDANMAL--CEQP-PKFEFYDPKTPFFTSPRYL
120  LNLLKS--RYAELHDFGSE--ILPRALH---D-H----NVQAYVF---TDYW--EDI--GT--IRSFFDANRAL--CEQP-PKFEFYDPKTPFFTSPRYL
121  IDLLND---HPQHKDFGKE--VIPEALA---GGM----TLKSYVF---DDYW--EDI--GT--IGAFYEANLAL--TQQPSPPFSFYDEDFPIYTRPRYL
122  FKLLKD---APDQTDFGKE--VIPGAAK---D-H----NVQAYLF---NDYW--EDI--GT--IEAFFEANLAL--TQQPQPAFSFYDENAPIYTRSRYL
123  IKLLKE---SLQSTDFGKE--IIPDASK---D-Y----NVQAYLF---DDYW--EDI--GT--IEAFYHANLAL--TKQPLPPFSFYDEKAPIYTRPRYL
124  NDLLKN---NPEQTDFGKE--IIPGAAK---D-Y----NLQAYLF---KGYW--EDI--GT--IEAFYEANLAL--NRQPRPRFSFYNEKAPIYTRARNL
125  IDLLKQ---QPDCTDFGKE--IIPNAIK---D-L----NIQAYLF---NDYW--EDI--GT--IEAFFNANLAL--AKQPNPSFSFYDKAAPIYTRARYL
126  FDLLAK---NPTATDFGKE--IIPEALS---RGD----NLQSFLF---DDYW--EDI--GT--IGAFYEANLAL--TDQPNPAFSFYDEQFPIYTRPRYL
127  FDLLAK---NPSATDFGKE--LIPASLE---RGD----HIQSYLF---DDYW--EDI--GT--IGAFYEANLAL--TDQPNPAFSFYEESFPIYTRPRYL
128  FDLLAQ---NPGSTDFGKE--IIPTALG---QGD----NLRAYLF---DDYW--EDI--GT--IGAFYEANLAL--TDQPRPAFSFYDEKFPIYTRPRYL
129  IKLLTE----TEQTDFGKE--ILPNAAP---D-Y----NLQAYLF---NDYW--EDI--GT--IEAFYNANLAL--TQQPQPPFSFYDEKAPIYTRSRYL
130  FDLLND---HPTYKDFGKE--VIPEALS---KGM----SLKSYVF---DDYW--EDI--GT--IGAFYEANLAL--TQQPKPPFSFYDEDFPIYTRPRYL
131  FDLLDK---HPGHKDFGKE--IIPEALK---RGD----KLQSYVF---DDYW--EDI--GT--IGAFYEANLAL--TQQPTPPFSFYDEKFPIYTRPRYL
132  FDLLHQ---NPTHKDFGKE--IIPEALA---RGD----RLKSYVF---DDYW--EDI--GT--IGAFYEANLAL--TQQPTPPFSFYDAEFPIYTRPRYL
133  LKLLRD---DPSRTDFGKE--ILPACLD---D-Y----NVQAYLF---DDYW--EDI--GT--IEAFYKANLAL--TSQNAPPFSFYHP-APIYTRPRYL
134  FDLLNK---FPSYTDFGKD--IIPEALS---RGD----TLKSYVF---DDYW--EDI--GT--IGAFFESNLAL--TQQPKPPFSFYDEKFPIYTRPRYL
135  FDLLNK---YPSYKDFGKE--VIPEALS---RGD----ALKSYVF---DAYW--EDI--GT--IGAFYESNLAL--TQQPTPPFSFYDEKFPIYTRARYL
136  IDLLQQ---MADATDFGKE--IIPAAAR---S-H----LVQTYLF---NGYW--EDI--GT--IGSFYEANLAL--TQQPQPPFSFYDENAPIYTRPRYL
137  AQLLEE---NPDQTDFGKE--IIPFSAK---D-Y----NLQAYLF---KGYW--EDI--GT--IKAFYEANLAL--NRQPSPRFSFYNEEYPIYTRSRYL
138  FKLLRE---SVERTDFGKE--IIPDASK---D-Y----NVQAYLF---DDYW--EDI--GT--IEAFYHANLAL--TQQPQPPFSFYDEHAPIYTRARYL
139  FDLLHK---NPSHKDFGKE--VIPEALA---RGD----RLQSYVF---DEYW--EDI--GT--IGAFYEANLAL--TQQPNPPFSFYDEKFPIYTRPRYL
140  IEMLKV----KEHTDFGKE--VLPAAIG---K-Y----HLQAYLF---KGYW--EDI--GT--IEAFYRANLAL--VQQPNPPFSFFDSEMPIYTRPRFL
141  IEMLKV----KEHTDFGKE--VLPSAIG---K-Y----HLQAYPF---KGYW--EDI--GT--IEAFYRANLAL--VQQPNPPFSFFDSEMPIYTRPRFL
142  FDLLHK---NPSHKDFGKE--VIPEALA---RGD----RLQSYVF---DEYW--EDI--GT--IGAFYEANLAL--TQQPNPPFSFYDEKFPIYTRPRYL
143  FDLLDS---NPGYKDFGKE--VIPEALK---RGD----KLKSYVF---DDYW--EDI--GT--IGAFYEANLAL--TQQPTPPFSFYDEKFPIYTRPRYL
144  IKLLKE---ALERTDFGKE--IIPDAAK---D-H----NVQAYLF---DDYW--EDI--GT--IEAFYNANLAL--TQQPMPPFSFYDEEAPIYTRARYL
145  FDLLNK---HPNYKDFGKE--VIPEALN---RGD----VLKSYVF---DDYW--EDI--GT--IGAFFESNLAL--TQQPKPPFSFYDEKFPIYTRARYL
146  FDLLNK---FPNYTDFGKD--IIPEALN---RGD----TLKSYVF---DDYW--EDI--GT--IGAFFESNLAL--TEQPKPPFSFYDEKFPIYTRPRFL
```

```
147  FDLLNK---FPSYTDFGKE--IIPEALG---RGD----KLKSYVF---NDYW--EDI--GT--IGAFFESNLAL--TQQPTPPFSFYDEKFPIYTRPRYL
148  FDLLNK---FPNYTDFGKD--IIPEALN---RGD----TLKSYVF---DDYW--EDI--GT--IGAFFESNLAL--TEQPKPPFSFYDEKFPIYTRPRFL
149  FDLLNK---FPNYTDFGKD--IIPEALG---RGD----NLKSYVF---DDYW--EDI--GT--IGAFFESNLAL--TRQPKPPFSFYDEKFPIYTRPRYL
150  FDLLDA---NPGHKDFGKE--VIPEALS---RGD----NLKSYVF---DDYW--EDI--GT--IGAFYEANLAL--TQQPTPPFSFYDEAFPIYTRPRYL
151  GKLLRQ---NPEQTDFGKE--IIPGAKT---D-Y----NLQAYLY---KGYW--EDI--GT--IEAFYESNLAL--TQQPQPPFSFYDEKAPIYTRPRYL
152  FDLLNK---FPNYTDFGKD--IIPEALK---RGD----TLKSYVF---DDYW--EDI--GT--IGAFFESNLAL--TEQPKPPFSFYDEKFPIYTRPRFL
153  HNLLEK---YEGATDFGKE--IIPDSAS---D-H----NLQAYLF---DDYW--EDI--GT--IEAFYEANLAL--TKQPSPDFSFYNEKAPIYTRGRYL
154  AQLLEE---NPDQTDFGKE--IIPFSAK---D-Y----NLQAYLF---KGYW--EDI--GT--IKAFYEANLAL--NRQPSPRFSFYNEEYPIYTRSRYL
155  IDLLSH---HPEQTDFGKE--VIPAAAT---R-Y----NTQAFLF---NDYW--EDI--GT--IASFYEANLAL--TQQPSPPFSFYDEQAPIYTRARYL
156  IDLLKQ---SPQSTDFGKE--IIPMAAT---D-H----NVQTYLF---NDYW--EDI--GT--ISSFYEANLAL--TRQPQPPFSFYDEKAPIYTRPRYL
157  IDLLSH---HPEQTDFGKE--VIPAAAT---R-Y----NTQAFLF---NDYW--EDI--GT--IASFYEANLAL--TQQPSPPFSFYDEQAPIYTRARYL
158  FDLLDK---HPGHKDFGKE--IIPEALA---RGD----KLQSYVF---DDYW--EDI--GT--IGAFYEANLAL--TQQPTPPFSFYDEKFPIYTRPRYL
159  FDLLDK---HPGHKDFGKE--IIPEALK---RGD----KLQSYVF---DDYW--EDI--GT--IGAFYEANLAL--TQQPTPPFSFYDEKFPIYTRPRYL
160  IDLLES---NIAHTDFGKE--VIPTAAA---N-H----NIQAFLF---DDYW--EDI--GT--IEAFYEANLAL--AQQPKPKFSFYDEQAPIYTRARYL
161  IDLLKN---SPDSTDFGKE--IIPSSAK---D-Y----NVQAYLF---NDYW--EDI--GT--IEAFYEANLAL--TRQPQPPFSFYDEKSPIYTRSRYL
162  IKLIED----SEDTDFGKE--ILPKSAQ---S-Y----NLQAYPF---QGYW--EDI--GT--IKSFYEANLAL--TQQPQPPFSFYDEQAPIYTRSRYL
163  IKLLKE---SLERTDFGKE--IIPDASK---D-H----NVQAYLF---DDYW--EDI--GT--IEAFYNANLAL--TQQPMPPFSFYDEEAPIYTRARYL
164  IDLLKQ---QPDCTDFGKE--IIPNAIK---D-L----NIQAYLF---NDYW--EDI--GT--IEAFFNANLAL--AKQPNPSFSFYDKAAPIYTRARYL
165  NDLLKN---NPEQTDFGKE--IIPGAAK---D-Y----NLQAYLF---KGYW--EDI--GT--IEAFYEANLAL--NRQPRPSFSFYNEKAPIYTRARNL
166  LDLLKE---GKDKTDFGKE--IIPDAAK---D-Y----NVQAYLF---DDYW--ADI--GT--IEAFYEANLGL--TKQPIPPFSFYDEKAPIYTRARYL
167  FDLLNK---FPSYTDFGKD--IIPEALN---RGD----KLKSYVF---DDYW--EDI--GT--IGAFFESNLAL--TEQPKPPFSFYDEKFPIYTRPRFL
168  FDLLNK---FPSYTDFGKE--IIPEALG---RGD----KLKSYVF---NDYW--EDI--GT--IGAFFESNLAL--TQQPTPPFSFYDEKFPIYTRPRYL
169  FRLLAE---NPGATDFGKE--IIPKALD---DGF----KLRSYLF---DDYW--EDI--GT--IRAFYEANLAL--TTQPRPPFSFYDKRFPIYTRHRYL
170  FDLLHQ---NPTHKDFGKE--VIPEALQ---RGD----RLKSYVF---DDYW--EDI--GT--IGAFYEANLAL--TQQPTPPFSFYDAEFPIYTRPRYL
171  IDLLDA---NKEQTDFGKE--IIPSAAK---D-Y----NLQAYLF---KGYW--EDI--GT--IEAFYESNLAL--TQQPNPAFSFYDEKAPIYTRSRYL
172  FDLLNK---FPSYTDFGKD--IIPEALN---RGD----SLKSYVF---DDYW--EDI--GT--IGAFFESNLAL--TEQPKPPFSFYDEKFPIYTRPRFL
173  FDLLNS---NPTATDFGKE--IIPASLA---RGD----QLRSYLF---DDYW--EDI--GT--IGAFYEANLAL--TQQPNPPFSFYDEKFPIYTRPRYL
174  GKLLRA---NLEQTDFGKE--IIPAASA---D-H----NVQAYLF---KGYW--EDI--GT--IEAFYESNLAL--TQQPYPAFSFYDEKAPIYTRARYL
175  IKLLKE---SLERTDFGKE--IIPDAAQ---D-H----NVQAYLF---DDYW--EDI--GT--IEAFYNANLAL--TQQPMPPFSFYDEAAPIYTRARYL
176  KEVLEA---NSEHTDFGNE--VIPASMP---K-Y----NIQAYLF---NDYW--QDI--GT--IEAFYNANLSL--TRQPSPSFSFYQEDAPIYTRARYL
177  IKLLKE---ASERTDFGKE--IIPDAAN---D-Y----NVQAYLF---NDYW--EDI--GT--IEAFYNANLTL--TQQPRPPFSFYDEQAPIYTRARYL
178  NDLLKN---NPEQTDFGKE--IIPGAAK---D-Y----NLQAYLF---KGYW--EDI--GT--IEAFYEANLAL--NRQPRPSFSFYNEKAPIYTRARNL
179  IDLLKQ---QPDCTDFGKE--IIPNAIK---D-L----NIQAYLF---NDYW--EDI--GT--IEAFFNANLAL--AKQPNPSFSFYDKAAPIYTRARYL
180  FDLLDA---NPGHKDFGKE--VIPEALS---RGD----VLKSYVF---DDYW--EDI--GT--IGAFYEANLAL--TQQPTPPFSFYDEAFPIYTRPRYL
181  TQLLEN---NPEQTDFGKE--VIPNAAV---Q-Y----NLQAYLF---DGYW--EDI--GT--VQAFYEANLAL--NHQPNPAFSFYNEQSPIYTHARYL
182  IKLLKE---SPNQTDFGKE--IIPASAK---D-H----NVQAYLF---DDYW--EDI--GT--IEAFYDANMAL--TKQPQPPFSFYDENAPIYTRPRFL
183  MKLLKE---SPEQTDFGKE--IIPNSAK---D-Y----NVQAYLF---DGYW--EDI--GT--IEAFYDSNLAL--TKQPHPPFSFYDEQAPIYTRQRYL
184  IKLLRE---SLEKTDFGKE--IIPDAAK---D-H----NVQAYLF---DGYW--EDI--GT--IEAFYNANLAL--TQQPVPPFSFYDEEAPIYTRARYL
```

```
185  IKLLRE---SLEKTDFGKE--IIPDAAK---D-H----NVQAYLF---DGYW--EDI--GT--IEAFYNANLAL--TQQPVPPFSFYDEEAPIYTRARYL
186  FKLLKD---APDQTDFGKE--VIPGAAK---D-H----NVQAYLF---NDYW--EDI--GT--IEAFFEANLAL--TQQPQPAFSFYDENAPIYTRSRYL
187  TDLLRN---NPEQTDFGKE--IIPGSAK---D-Y----NLQAYLF---KGYW--EDI--GT--IEAFYEANLAL--NRQPLPRFSFYNEKAPIYTRARNL
188  FDLLDK---HPGHKDFGKE--IIPEALA---RGD----KLQSYVF---DDYW--EDI--GT--IGAFYEANLAL--TQQPTPPFSFYDEKFPIYTRPRYL
189  ISFLNS--EYPKDNDFGGE--IIPKAAA---DGY----HVQAYLF---KDYW--EDI--GT--IKSFFEANLAL--AKHP-PQFEFYDARAPIYTSPRFL
190  DELLTE--KFPDCHDFGGE--IIPKANE---LGK----HVQAFLY---KGYW--EDI--GT--IEAFYNANLQC--NDPDAPKFSFYESGSPIYTQSRFL
191  LNFLNA--EYPKDNDFGGE--IIPKAAA---DGY----HVQAYLF---NDYW--EDI--GT--IKSFFEANLAL--AKNP-PQFEFYDARAPIYTSPRFL
192  EKLLME--DFPTCHDFGGE--IIPNAKD---LGM----HVQAFLY---DGYW--EDI--GT--IKAFFDANLAC--NDPEKAKFSFYQTGAPIYTQSRFL
193  LQLLND--SYAKANDFGGE--IIPSAAK---D-H----NVVAYPF---YGYW--EDI--GT--IKSFFEENLKL--CRHP-ATFEFYDPQSPIYTSPRVL
194  RELLLN--RMPGANDFGNE--VIPGAKD---AGF----KVQAFAF---DGYW--EDI--GT--VEAFYNANLAL--TDPEKAQFSFYDKDAPIYTMSRFL
195  VKFLEK--DYPEDNDFGGE--IIPRAAA---DGA----KVQAYLF---NDYW--EDI--GT--MKSFFEANLNL--AKDP-PNFEFYNAEAPIYTSPRFL
196  RECLLE--NFKEADDFGGE--IIPMAAQ---MGL----KVQAFLY---EGYW--EDI--GT--VDAFFHANLSC--NDPN-PAFNFHEMNAPIYTQSRFL
197  RELLLN--RMPGANDFGNE--VIPGAKD---AGY----KVQAYAF---KGYW--EDI--GT--VEAFYNANLAL--ADPSKAQFSFYDKDAPIYTMSRFL
198  CKLLNE--TYAKANDFGGE--IIPEAAK---N-H----NVVAYPF---YGYW--EDI--GT--IKSFFEENLKL--CRHP-ATFEFYDPQSPIYTSPRVL
199  VKFLDD--DYPEDNDFGGE--IIPKASA---DGA----RVQAYLF---NDYW--EDI--GT--MKSFFEANLAL--AKDP-PNFEFYNAEAPIYTSPRFL
200  EQVLQD--DFPEANDFGGE--IIPMAAQ---KGM----KVVAHLY---DGYW--EDI--GT--VDAFFHANLEC--NDPN-PKFSFYDRNAPIYTQSRFL

                 410       420       430       440       450       460       470       480       490       500
     ....|....|....|....|....|....|....|....|....|....|....|....|....|....|....|....|....|....|....|....|
1    PP--SKMLDADVTDSVIG--EG--CVIK--N---CKIHHSVVG--LR--SCI--SEG--AIIEDSLLM--GADYYET--DADRKLLAAK--GSVPI--GI
2    PP--SKMLDADVTDSVIG--EG--CVIK--N---CKIHHSVVG--LR--SCI--SEG--AIIEDSLLM--GADYYET--DADRKLLAAK--GSVPI--GI
3    PP--SKMLDADVTDSVIG--EG--CVIK--N---CKIHHSVVG--LR--SCI--SEG--AIIEDSLLM--GADYYET--DVDRKLLAAK--GSVPI--GI
4    PP--SKMLDADVTDSVIG--EG--CVIK--N---CKIHHSVVG--LR--SCI--SEG--AIIEDSLLM--GADYYET--DADRKLLAAK--GSVPI--GI
5    PP--SKMLDADVTDSVIG--EG--CVIK--N---CKIHHSVVG--LR--SCI--SEG--AIIEDSLLM--GADYYET--ATEKSLLSAK--GSVPI--GI
6    PP--SKMLDADVTDSVIG--EG--CVIK--N---CKIHHSVVG--LR--SCI--SEG--AIIEDSLLM--GADYYET--ASEKSLLSAK--GSVPI--GI
7    PP--SKMLDADITDSVIG--EG--CVIK--N---CKIFHSVVG--LR--SCI--SEG--AIIEDTLLM--GADYYET--EADKSFLAAK--GSVPI--GI
8    PP--SKMLDADITDSVIG--EG--CVIK--N---CKIHHSVVG--LR--SCI--SEG--AIIEDTLLM--GADYYET--DADRRFLAAK--GSVPI--GI
9    PP--SKMLDADVTDSVIG--EG--CVIK--N---CKIHHSVVG--LR--SCI--SEG--AIIEDTLLM--GADYYET--EADKRFLAAK--GSVPI--GI
10   PP--SKMLDADVTDSVIG--EG--CVIK--N---CKIHHSVVG--LR--SCI--SEG--AIIEDTLLM--GADYYET--EADKRFLAAK--GSVPI--GI
11   PP--SKMLDADVTDSVIG--EG--CVIK--N---CKIHHSVIG--LR--SCI--SEG--AIIEDTLLM--GADYYET--DADRRFLAAK--GSVPI--GI
12   PP--SKMLDADVTDSVIG--EG--CVIK--N---CKIHHSVVG--LR--SCI--SEG--AIIEDTLLM--GADYYET--DADRRFLAAK--GSVPI--GI
13   PP--SKMLDADVTDSVIG--EG--CVIK--N---CKIHHSVVG--LR--SCI--SEG--AIIEDTLLM--GADYYET--DADRRFLMAK--GSVPI--GI
14   PP--SKMLDADVTDSVIG--EG--CVIK--N---CKIHHSVIG--LR--SCI--SEG--AIIEDTLLM--GADYYET--DADRTLLAAK--GRVPI--GI
16   PP--SKMLDADITDSVIG--EG--CVIK--N---CKIHHSVVG--LR--SCI--SEG--AIIEDTLLM--GADYYET--DADRRFLAAK--GGVPI--GI
17   PP--SKMLDADITDSVIG--EG--CVIK--N---CKIFHSVVG--LR--SCI--SEG--AIIEDTLLM--GADYYET--EADKRFLAAK--GSVPI--GI
18   PP--SKMLDADITDSVIG--EG--CVIK--N---CKIHHSVVG--LR--SCI--SEG--AIIEDTLLM--GADYYET--DADRRFLAAK--GSVPI--GI
19   PP--SKMLDADITDSVIG--EG--CVIK--N---CKIFHSVIG--LR--SCI--SEG--AIIEDTLLM--GADYYET--DADKRFLAAK--GSVPI--GI
20   PP--SKMLDADITDSVIG--EG--CVIK--N---CKIHHSVVG--IR--TCI--SEG--AIIEDTLLM--GADYYET--DADRRLLAAK--GSVPI--GI
```

```
21   PP--SKMLDADITDSVIG--EG--CVIK--N---CKIHHSVVG--IR--TCI--SEG--AIIEDTLLM--GADYYET--DADRRLLAAK--GSVPI--GI
22   PP--SKMLDADVTDSVIG--EF--CVIK--N---CKIHHSVVG--LR--SCI--SEG--AIIEDTLLM--GADYYET--DADRRFLAAK--GSVPI--GI
23   PP--SKMLDADVTDSVIG--EG--CVIK--N---CKIHHSVVG--LR--SCI--SEG--AIIEDTLLM--GADYYET--DADRRFLAAK--GSVPI--GI
24   PP--SKMLDADITDSVIG--EG--CVIK--N---CKIHHSVIG--LR--SCI--SEG--AVIEDTLLM--GADYYET--DVDRRLMAKK--GSVPI--GI
25   PP--SKMLDADVTDSVIG--EG--CVIK--N---CKIHHSVVG--LR--SCI--SEG--AIIEDTLLM--GADYYET--DADRRLLAAK--GSVPI--GI
26   PP--SKMLDADVTDSVIG--EG--CVIK--N---CKIHHSVVG--VR--SCI--SEG--AIIEDSLLM--GADYYET--DADRRLLAAK--GSIPI--GI
27   PP--SKMLNADVTDSVIG--EG--CVIK--N---CKIHHSVIG--LR--SCI--SEG--AIIEDTLLM--GADYYET--DSDRRILAAK--GGIPI--GI
28   PP--SKMLDADVTDSVIG--EG--CVIK--N---CKIHHSVVG--LR--SCI--AEG--AIIEDTLLM--GADYYET--DADRRFLAAK--GGVPI--GI
29   PP--SKMLDADVTDSVIG--EG--CVIK--N---CKIHHSVVG--LR--SCI--SEG--AIIEDTLLM--GADYYET--DADKRFLAAK--GSVPI--GI
30   PP--SKMLDADITDSVIG--EG--CVIK--N---CKIHHSVVG--LR--SCI--SEG--AIIEDTLLM--GADYYET--DADRRFLAAK--GGVPI--GI
31   PP--SKMLDADITDSVIG--EG--CVIK--N---CKIFHSVVG--LR--SCI--SEG--AIIEDTLLM--GADYYET--EADKRFLAAK--GSVPI--GI
32   PP--SKVLDADVTDSVIG--EG--CVIK--H---CTINHSVVG--LR--SCI--SEG--AVIEDSLLM--GADYYET--ENDKNVLSET--GGIPI--GI
33   PP--SKVLDADVTDSVIG--EG--CVIK--N---CKINHSVVG--LR--SCI--SEG--AIIEDSLLM--GADYYET--EADKKLLAEK--GGIPI--GI
34   PP--SKVLDADVTDSVIG--EG--CVIK--N---CKINHSVVG--LR--SCI--SEG--AIIEDSLLM--GADYYET--EADKKLLAEK--GGIPI--GI
36   PP--SKVLDADVTDSVIG--EG--CVIK--N---CKIHHSVVG--LR--SCI--SEG--AIIEDTLLM--GADYYET--EADKKLLAEN--GGIPI--GI
37   PP--SKVLDADVTDSVIG--EG--CVIK--N---CKIHHSVVG--LR--SCI--SEG--AIIEDSLLM--GADYYET--EADKKLLGEK--GGIPI--GI
38   PP--SKVLDADVTDSVIG--EG--CVIR--H---CTINHSVVG--LR--SCI--SEG--AVIEDSLLM--GADYYET--ETDKKALSET--GGIPI--GI
39   PP--SKVLDADVTDSVIG--EG--CVIK--N---CKIHHSVVG--LR--SCI--SEG--AIIEDSLLM--GADYYET--EADKKLLGEK--GGIPI--GI
41   PP--SKVLDADVTDSVIG--EG--CVIK--N---CKIHHSVVG--LR--SCI--SEG--AIIEDTLLM--GADYYET--EADKQLLAEK--GGIPI--GI
42   PP--SKVLDADVTDSVIG--EG--CVIK--N---CKIHHSVVG--LR--SCI--SEG--AIIEDTLLM--GADYYET--EADKQLLAEK--GGIPI--GI
43   PP--SKVLDADVTDSVIG--EG--CVIK--H---CTINHSVVG--LR--SCI--SEG--AVIEDSLLM--GADYYET--EDDKKVLSEN--GGIPI--GI
44   PP--SRVLDADVTDSVIG--EG--CVIN--H---CKINHSVVG--LR--SCI--SEG--AVIEDSLLM--GADYYET--ENDKKVLSET--GGIPI--GI
45   PP--SKVLDADVTDSVIG--EG--CVIK--N---CKIHHSVVG--LR--SCI--SEG--AIIEDTLLM--GADYYET--EADKKLLAEK--GGIPI--GI
46   PP--SKVLDADVTDSVIG--EG--CVIK--N---CKIHHSVVG--LR--SCI--SEG--AIIEDTLLM--GADYYET--EADKKLLAEK--GGIPI--GI
47   PP--SKVLNADVTDSVIG--EG--CVIN--H---CTINHSVVG--LR--SCI--SEG--AVIEDSLLM--GADYYET--ENDKKVLSES--GGIPI--GI
48   PP--SKVLDADVTDSVIG--EG--CVIK--N---CKIHHSVVG--LR--SCI--SEG--AIIEDTLLM--GADYYET--EADKKLLAEK--GGIPI--GI
49   PP--TKIDNCKIKDAIIS--HG--CFLR--D---CSVEHSIVG--ER--SRL--DCG--VELKDTFMM--GADYYQT--ESEIASLLAE--GKVPI--GI
50   PP--TKVDKSRIVDAIIS--HG--CFLR--E---CNIQHSIVG--VR--SRL--DYG--VEFKDTMMM--GADYYQT--ECEIASLLAE--GKVPI--GV
51   PP--SAIDNSKIVDSIVS--HG--IFLT--N---CFVEHSVVG--IR--SRI--GTN--VHLKDTVML--GADYYET--DAEIRSQLAE--GKVPL--GI
52   PP--SKIDNSKLIDSIIS--HG--SFLT--N---CLIEHSIVG--IR--SRV--GSN--VQLKDTVML--GADYYET--EAEVAALLAE--GNVPI--GI
53   PP--TKVDKCRILDSIVS--HG--CFLR--E---CSVQHSIVG--IR--SRL--ESG--VELQDTMMM--GADFYQT--EAEIASLLAE--GKVPV--GV
54   PP--TKTEKCRIVNSVIS--HG--CFLG--E---CSIQRSIIG--ER--SRL--DYG--VELQDTLML--GADSYQT--ESEIASLLAE--GNVPI--GI
55   PP--TKAEKCRMVDSIIS--HG--CFLR--E---CSVQRSIIG--ER--SRL--DYG--VELQDTLML--GADYYQT--ESEIASLLAE--GKVPI--GI
56   PP--TKIDNCKIKDAIIS--HG--CFLR--D---CTVEHSIVG--ER--SRL--DCG--VELKDTFMM--GADYYQT--ESEIASLLAE--GKVPI--GI
57   PP--TKVDKSRIVDAIIS--HG--GFLR--E---CNIQHSIVG--VR--SRL--DYG--VEFKDTMMM--GADYYQT--ESEIASLLAE--GKVPI--GV
58   PP--SAIDNSKIVDSIVS--HG--SFLT--N---CFVEHSVVG--IR--SRI--GTN--VHLKDTVML--GADYYET--DAEIASQLAE--GKVPL--GI
59   PP--TKIDNCKIKDAIIS--HG--CFLR--D---CTVEHSIVG--ER--SRL--DCG--VELKDTFMM--GADYYQT--ESEIASLLAE--GKVPI--GI
60   PP--TKVDKSRIVDAIIS--HG--CFLR--E---CNIQHSIVG--VR--SRL--DYG--VEFKDTMMM--GADYYQT--ESEIASLLAE--GKVPI--GV
```

```
61   PP--SKIDNSKLVDSIIS--HG--SFLT--N---CLIEHSIVG--IR--SRV--GSN--VQLKDTVML--GADYYET--EAEVASLLAE--GKVPI--GI
62   PP--TKVDKCRILDSIVS--HG--CFLR--E---CSVQHSIVG--IR--SRI--ESG--VELQDTMMM--GADFYQT--EAEIASLLAE--GKVPV--GV
63   PP--TKTEKCRIVNSIIS--HG--CFLG--E---CSIQRSIIG--ER--SRL--DYG--VELQDTLML--GADSYQT--ESEIASLLAE--GNVPI--GI
64   PP--TKAEKCRMVDSIIS--HG--CFLR--E---CSIQRSIIG--ER--SRL--DYG--VELQDTLML--GADYYQT--ESEIASLLAE--GKVPI--GI
66   PP--TKMEKCEVINSLIS--DG--CFLK--E---CTVEHSIVG--IR--SRL--DSG--VQLKDTMIM--GADYYQT--EAEIASLLAA--GNVPI--GI
67   PP--SKIDNSKIVDSIIS--HG--SFLN--N---SFIEHSVVG--IR--SRI--NSN--VHLKDTVML--GADYYET--DAEVVALLAE--GRVPI--GI
68   PP--TKAIKCKIMDAIIS--HG--CFLS--E---SRVQHSIVG--VR--SRL--ESG--SELQDTMMM--GADYYQT--DSEIATLLEE--GKVPI--GV
69   PP--TKAIKCKIVDAIIS--HG--CFLS--E---CRVQHSIVG--VR--SRL--ESG--SELQDTMMM--GADYYQT--DSEIATLLKE--GKVPI--GV
70   PP--TKIDKCRIVDAIIS--HG--CFLR--E---CTVQHSIVG--ER--SRL--DYG--VELQDTVMM--GADYYQT--ESEIASLLAE--GKVPI--GI
71   PP--TKIDKCRIVDAIIS--HG--CFLR--E---CTVQHSIVG--ER--SRL--DYG--VELQDTVMM--GADYYQT--ESEIASLLAE--GKVPI--GI
72   PP--SKIDNSKIVDSIIS--HG--SFLN--N---SFIEHSVVG--IR--SRI--NSN--IHLKDTVML--GADYYET--DAEVAALLAE--GRVPI--GI
73   PP--TKVEKCKIVDAIIS--HG--CFLR--E---CSVQHSIVG--VR--SRL--ESG--VELQDTMMM--GADYYQT--EYEIASLVAE--GKVPI--GV
74   PP--TKIDKCRIVDAIIS--HG--CFLR--E---CTVQHSIVG--ER--SRL--DYG--VEVLDTVMM--GADYYQT--ESEIASLLAE--GKVPI--GI
75   PP--TKVEKCKIVDAIIS--HG--CFLR--E---CSIQHSIVG--VR--SRL--ESG--VELQDTMMM--GADYYQT--EYEIASLLAE--GKVPI--GV
76   PP--TKIDKCQIVDAIIS--HG--CFLR--E---CTVQHSIVG--ER--SRL--DYG--VELLDTVMM--GADYYQT--ESEIASLLAE--GKVPI--GI
77   PP--TKMEKCEVINSLIS--DG--CFLK--E---CTVEHSIVG--IR--SRL--DSG--VQLKDTMIM--GADYYQT--EAEIASLLAA--GNVPI--GI
80   PP--TKIDKCRIVDAIIS--HG--CFLR--E---CSVQHSIVG--ER--SRL--DYG--VELQDTVMM--GADYYQT--ESEIASELAE--GKVPI--GI
81   PP--TKIEKCRVKDSIVS--HG--CFLR--E---CSVERSIVG--VR--SRL--EYG--VELKDTMMI--GADYYQT--EAEIAASLAE--GRVPV--GV
82   PP--TKFDKCRIVNAIIS--HG--CFLR--E---CTVQHSVVG--ER--SRL--DYG--VELKDTVML--GADCYQT--EVEIASLLAE--GEVPI--GV
84   PP--SKIDSSKIVDSIIS--HG--SFLN--N---CFIEHSVIG--IR--SRI--NSN--AHLQDTVML--GADFYET--EAEVASVVAE--GSVPV--GI
87   PP--SKIENCKIVDSIIS--HG--SFLT--N---SFIEHSVVG--IR--SRI--NSN--VHLKDTVML--GADFYET--DDEVAALLAE--GRVPI--GI
88   PP--TKIERCQVKDSIIS--HG--CFLR--E---CSVEHSIVG--VR--SRL--EYG--VELKDTMMM--GADYYQT--EAEVAASLAG--GKVPI--GV
89   PP--TKIDKCRIVDAIIS--HG--CFLR--E---CTVRHSVVG--ER--SRL--DYG--VELKDTVML--GADYYQT--ETEIASLLAE--GKVPI--GV
90   PP--TKVDQCRIVDAIIS--HG--CFLQ--E---CSIKHSIVG--VR--SRL--ESA--VELMDTMMM--GADYYQT--ESEIASLQAE--GKVPI--GV
91   PP--TKVEECRILDAIIS--HG--CFLR--E---CSVQRSIVG--VR--SRL--EYG--VELKDTMMM--GADYYQT--ESEIASLLAE--GKVPI--GV
92   PP--TKIEKCRVMDSIIS--HG--CFLR--E---CSVEHSIVG--IR--SRL--DYG--VEMKDTMMM--GADYYQT--EEEIAAFLAE--GKVPI--GV
94   PP--SKIDDSKIVDSIIS--HG--SFLN--N---CFIEHSVVG--IR--SRV--NSN--VHLKDTVML--GADYYET--DSEVASLLAE--GRVPI--GI
95   PP--TKIEQCQVVDAIIS--HG--CFLR--E---CSVKHSIVG--ER--SRL--DYG--VELKDTLMM--GADFYQT--ESEIASLLAE--GNVPI--GI
96   PP--TKVDRCRIVDAIIS--HG--CFLR--E---CSVQHSIVG--VR--SRL--ESG--VELTDTMMM--GADYYQT--ESEIASLLAE--GKVPI--GV
97   PP--TKIDKCRIVDAIIS--HG--CFLR--E---CTVQHSVVG--ER--SRL--DYG--VELKDTVML--GADHYQT--EAEIASLLAE--GKVPI--GV
98   PP--TKVDKCRIVDAIIS--HG--CFLR--E---CSVQHSIVG--VR--SRL--ESG--VELTDTMMM--GADYYQT--ESEIASVLAE--GKVPI--GV
99   PP--SMVNNSKITDSIIS--HG--CFLD--N---CRIEHSVVG--VR--SRI--GSN--VHLKDTVML--GADYYET--AVERGELLAE--GKVPI--GI
100  PP--TQLDKCKMKYAFIS--DG--CLLR--E---CNIEHSVIG--VC--SRV--SSG--CELKDSVMM--GADTYET--EEEASKLLLA--GKVPV--GI
101  PP--TKSDKCRIKDAIIS--HG--CFLR--E---CAIEHSIVG--VP--SRL--NSG--CELKNTMMM--GADLYET--EDEISRLLAE--GKVPI--GV
102  PP--TKVENCKVLNSIVS--HG--CFLT--E---CSVEHSVIG--IR--SRL--EPG--VQLKDTMMM--GADYYQT--EAERLSELSV--GKVPV--GV
103  PP--TKSDKCRIKEAIIS--HG--CFLR--E---CTIEHSIVG--VR--SRL--NSG--CELKNAMMM--GADLYET--EDEISRLLSE--GKVPI--GV
104  PP--TKVEDCKVLNSIVS--HG--CFLT--E---CSVEHSVIG--IR--SRL--QPG--VQLKDTMMM--GADYYQT--EAERFSELSD--GKVPV--GV
105  PP--SMISSSKITDSIIS--HG--CFLD--N---CRVEHSVVG--VR--SRV--GSN--VHLKDTVML--GADFYET--DVERSDQLAE--GKVPI--GI
```

```
106  PP--SMINNSKITDSIIS--HG--CFLD--S---CRIEHSVVG--IR--SRI--GSN--VHLKDTVML--GADFYET--DLERGELLAE--GKVPI--GI
107  PP--TKSDKCRIKDAIIS--HG--CFLR--E---CTIEHSIVG--VR--SRL--NSA--CELKNTMMM--GADLYET--EDEISRLLSE--GKVPI--GV
108  PP--TKVENCKVLNSIVS--HG--CFLT--E---CSVDRSVIG--VR--SRL--EPG--VQLKDTMMM--GADYYQT--EAERFSELSD--GKVPV--GV
109  PP--ARLEKCKIKDAIIS--DG--CSFS--E---CTIEHSVIG--IS--SRV--SIG--CELKDTMMM--GADQYET--EEETSKLLFE--GKVPI--GI
110  PP--TKSDKCRIKDAIIS--HG--CFLR--E---CAIEHSIVG--VR--SRL--NSG--CELKNTMMM--GADLYET--EDEISRLLSE--GKVPI--GV
111  PP--TQLDKCKIKDASIS--DG--CLLR--E---CSIEHSVIG--VC--SRV--SYG--CELKDCVMM--GADIYET--EEEASKLLLA--GEVPV--GI
112  PP--SMVNNSKITDSIIS--HG--CFLD--N---CRIEHSVVG--VR--SRI--GSN--VHLKDTVML--GADYYET--DAERRELLAE--GNVPI--GI
113  PP--TKSDKCRIKEAIIS--HG--CFLR--E---CKIEHSIIG--VR--SRL--NSG--SELKNAMMM--GADSYET--EDEISRLMSE--GKVPI--GV
114  PP--SMISGSKITDSIIS--HG--CFLD--K---CRVEHSVVG--IR--SRI--GSN--VHLKDTVML--GADFYET--DAERGDQLAE--GKVPI--GI
115  PP--TKSDKCRIKEAIIS--HG--CFLR--E---CKIEHSIIG--VR--SRL--NSG--SELKNAMMM--GADSYET--EDEISRLMSE--GKVPI--GV
116  PP--TAINNSKIVDSIIS--HG--SLLS--N---CLIEHSVVG--IR--SRI--NDN--VHLKDTVML--GADLYET--DAEIAALLAE--GRVPV--GI
117  PP--TKSDKCRIKEAIIS--HG--CFLR--E---CKIEHSIIG--VR--SRL--NSG--SELKNAMMM--GADSYET--EDEISRLMSE--GKVPI--GV
118  PP--SMISGSKITDSIIS--HG--CFLD--K---CRVEHSVVG--IR--SRI--GSN--VHLKDTVML--GADFYET--DMERGDQLAE--GKVPI--GI
119  PP--TKSDKCRIKEAIIS--HG--CFLR--E---CKIEHSIIG--VR--SRL--NSG--SELKNAMMM--GADSYET--EDEISRLMSE--GKVPI--GV
120  PP--TKSDKCRIKEAIIL--HG--CFLR--E---CKIEHTAF-------SRL--NSG--SELKNAMMM--GADSYET--EDEMSRLMSE--GKVPI--GV
121  PP--SKLVDAQITESIIG--EG--TILK--S---CSIHHCVLG--VR--SRV--END--VVLQDSLLM--GADFFES--STERSVLRER--GGIPV--GV
122  PP--SKMLDCQITESIIA--EG--CILK--E---CRIDHSVLG--LR--SRV--ESG--SLVEDTMLM--GSDFYQP--FAERQYGLEK--GSVPI--GI
123  PP--SKLLSCHVTESIIG--EG--CILK--D---CRIQHSVLG--VR--SRI--EAG--CVIEESLLM--GADFYQP--FVERQCNLEK--GDIPV--GI
124  PP--TKVLNSNITESMIS--EG--CMIK--D---CRIHNSVLG--IR--SRI--ETD--CVVEDSLLM--GADYYES--LDDRQSLLDQ--GKIPI--GI
125  PP--TKQQKCQVIESMIS--EG--CILK--E---CYIENSVIG--IR--SRI--DSG--CTIKNVLLM--GADYYQS--DFENEGDCSL--ENIPI--GI
126  PP--SKMLDAQVTQSIIG--EG--SMLK--A---CSIHHCVLG--VR--TRV--EDE--AVLQDTLVM--GSDFFES--SEERAVLRER--GGIPL--GV
127  PP--SKLLDSQVTQSIIG--EG--SILK--A---CSIHHCVLG--VR--SRV--EED--AVLQDTLVM--GNDFFES--SAERNALRHR--GGTPV--GV
128  PP--SKLLDAQVTQSIIG--EG--SLLQ--D---CSIHHCVLG--VR--SRI--ESE--VVLQDTLVM--GADFFES--SEERAVLRER--GGIPV--GV
129  PP--SKILDCQITESIIS--EG--SILK--Q---CRIGHSVLG--LR--SRI--EAG--CVIEDTLVM--GSDYYEP--FAERQSNIQQ--GKIPM--GI
130  PP--SKVGDSQIIDSIIG--EG--SIIK--S---CSVNHCVLG--IR--SRI--ENS--VVVQDSLVM--GSDFYES--TQEREELRRN--GGIPL--GV
131  PP--SKLVDAQIVNSIIG--EG--SILK--S---CSINHCVLG--VR--SRV--ETD--VVLQDTLVM--GADFFES--NDEREAIRQK--GGIPV--GV
132  PP--SKLVDSQITDSIIG--EG--SILK--S---CSIHHSVLG--VR--SRV--EDD--VVLQDSLLM--GSDFFES--SSERAVLKER--GGIPL--GV
133  PP--SKLIDCQIAESIIT--EG--CIIK--Q---ARIFHSVLG--LR--SRI--ESG--VRIEDSLLM--GADFYET--PIQREESLRR--GLPPV--GI
134  PP--SKLVDAQITDSIVC--EG--TILK--S---CSILHCVLG--VR--SRI--ESD--SVIEDTLVM--GSDFFES--LEERIELRKG--GGTPL--GV
135  PP--SKLVDAQITDSIVG--EG--SILK--S---CSIHHCVLG--VR--SRI--ESD--VVLEDSLVM--GSDFYES--AEERIALRKG--GGIPL--GV
136  PP--SKILSSTITESIIS--EG--CILK--E---CQVHRSVLG--VR--SRV--ESG--CVIDHSLLM--GADYYQD--SAQRSQLRLQ--HKIPI--GI
137  PP--TKALNCTITESMVS--EG--CILK--D---CRIHNSILG--IR--TRI--EAN--CTIEDTMLM--GADYYES--PSLRESKAQE--GKIPM--GI
138  PP--TKLLDCQITESIIG--EG--CILK--N---CRIQHSVLG--VR--SRI--ESG--CVIEESLLM--GADFYQA--SVERQCSLIE--NDIPV--GI
139  PP--TKLVDAQITESIIG--EG--SILK--S---CSIHHCVLG--VR--SRV--ESD--VVLQDSLVM--GSDFYES--SEERTLLRQG--GGIPL--GV
140  PP--NKILDSQIVNSMIA--DG--CIIK--N---AQIRNSIIG--IR--SRL--EAN--TIIENTLVM--GADYYES--AEERQARLEE--GIPPV--GI
141  PP--NKILDSQIVNSMIA--DG--CIIK--N---AQIRNSIIG--IR--SRL--EAN--TIVENTLVM--GADYYES--AEERQAKLEA--GIPPV--GI
142  PP--TKLVDAQITESIIG--EG--SILK--S---CSIHHCVLG--VR--SRV--ESD--VVLQDSLVM--GSDFYES--SEERTLLRQG--GGIPL--GV
143  PP--SKLVDAQITNSIVG--EG--SILK--S---CSIHHCVLG--VR--SRI--ETD--VVLQDTLVM--GADFFES--SDERAVLRER--GGIPV--GV
```

```
144  PP--TKLLDCHVTESIIG--EG--CILK--N---CRIQHSVLG--VR--SRI--ETG--CMIEESLLM--GADFYQA--SVERQCSIDK--GDIPV--GI
145  PP--SKLVDAQITDSIVG--EG--SILK--A---CSIHHCVLG--VR--SRI--ESD--VVLQDTLVM--GSDFYES--GEERIALRSG--GGIPL--GV
146  PP--SKLVDAQITDSIVC--EG--TILK--S---CSILHCVLG--VR--SRI--ESD--SVLEDTLVM--GADFFES--PEERFELRKG--GGTPL--GV
147  PP--SKIVDTQITDSIVS--EG--SILK--S---CSIHHCVLG--VR--SRI--ESD--VVLNETLVM--GSDFYES--YEERIALRNG--GGIPL--GV
148  PP--SKLVDAQITDSIVC--EG--TILK--S---CSILHCVLG--VR--SRI--ESD--SVLEDTLVM--GADFFES--PEERIELRKG--GGTPL--GV
149  PP--SKLVDAQITDSIVC--EG--TILK--S---CSILHCVLG--VR--SRI--ESD--SVIEDALVM--GADFFES--QEERVELRKG--GGTPL--GV
150  PP--SKFVDSQITDSIIS--EG--SIIK--A---CSIHHSVLG--VR--SRV--ENN--VVLQDSLLM--GADFFES--QGERETLRAR--GGIPV--GV
151  PP--TKVLNCTITESMIS--EG--CILK--D---CRIHHSVLG--IR--SRV--ESD--CTIEDSMLM--GADYYES--STKRKAVLEA--GKVPQ--GI
152  PP--SKLVDAQITDSIVC--EG--TILK--S---CSILHCVLG--VR--SRI--ESD--SILEDTLVM--GADFFES--PEERIELRKG--GGTPL--GV
153  PP--TKMLNSTVTESMIG--EG--CMIK--Q---CRIHHSVLG--IR--SRI--ESD--CTIEDTLVM--GNDFYES--SSERDTLKAR--GEIAA--GI
154  PP--TKALNCTITESMVS--EG--CILK--D---CRIHNSILG--IR--TRI--EAN--CTIEDTMLM--GADYYES--PSLRESKAQE--GKIPM--GI
155  PP--TKLLDCQVTQSIIG--EG--CILK--Q---CTVQNSVLG--IR--SRI--EAD--CVIQDALLM--GADFYET--SELRHQNRAN--GKVPM--GI
156  PP--SKLLDCQVTESIIG--EG--CILK--N---CQIQHSVLG--VR--SRI--ESG--CVIDNALLM--GADFYQP--FAERDHKIKN--NSVPL--GI
157  PP--TKLLDCQVTQSIIG--EG--CILK--Q---CTVQNSVLG--IR--SRI--EAD--CVIQDALLM--GADFYET--SELRHQNRAN--GKVPM--GI
158  PP--SKLVDAQITNSIVG--EG--SILK--S---CSIHHCVLG--VR--SRI--ESD--CVLQDTLVM--GADFFES--PDERAVLKER--GGIPL--GV
159  PP--SKLVDAQIVNSIIG--EG--SILK--S---CSINHCVLG--VR--SRV--ETD--VVLQDTLVM--GADFFES--NEERETIRQQ--GGIPV--GV
160  PP--SKILDCRVTESIVG--EG--CIVK--K---SQIHHSVLG--VR--SYV--DDH--CTLDNVLWL--GSDYYQS--LSERQADLDQ--GRVPL--GI
161  PP--TKQLDCHVTESMIA--EG--CIIK--N---CQINRSVLG--VR--SRV--ESG--CTLDNALVM--GADYYQP--FAERASGMGD--TSIPI--GI
162  PP--SKLLDCEITESIVG--EG--CILK--K---CRIDHCVLG--VR--SRI--EAN--CIIQDSLLM--GSDFYES--PTERRYGLKK--GSVPL--GI
163  PP--TKLLDCHVTESIIG--EG--CILK--N---CRIQHSVLG--VR--SRI--ETG--CVIEESLLM--GADFYQA--SVERQCSIDK--GDIPV--GI
164  PP--TKQLKCEVIQSMIS--EG--CVLK--D---CYIENSVIG--IR--SRI--DSG--CTIKNVLLM--GADYYQS--DFENEGDCSL--ENIPI--GI
165  PP--TKVLNCNITESMIS--EG--CMIK--D---CRIHNSVLG--IR--SRI--ETD--CVVEDSLLM--GADYYES--LETRQSLLDQ--GKIPV--GI
166  PP--TKVLNADVTESMIS--EG--CIIK--N---CRIHHSVLG--IR--TRV--EAD--CTIEDTMIM--GADYYQP--YEKRQDCLRR--GKPPI--GI
167  PP--SKLVDAQITDSIVC--EG--TILK--S---CSILHCVLG--VR--TRI--ESD--SVLEDTLVM--GADFFES--PEERIELRRG--GGTPL--GV
168  PP--SKIVDTQITDSIVS--EG--SILK--S---CSIHHCVLG--VR--SRI--ESD--VVLNETLVM--GSDFYES--YEERIALRNG--GGIPL--GV
169  PP--SKLQDAQVTDSIVG--EG--SILK--A---CSIHHCVLG--VR--SRI--EDE--VALQDTLVM--GNDFYES--GEERAILRER--GGIPM--GV
170  PP--SKLVDSQITDSIIG--EG--SILK--S---CSIHHSVLG--VR--SRV--EDE--VVLQDSLLM--GSDFFES--SSERAVLRER--GGIPL--GV
171  PP--TKMLNCTVTESMIS--EG--CILK--E---CRIHHSILG--IR--SRV--GKD--CTIEDTMLM--GADFYES--FPERESLIGN--AKIPV--GI
172  PP--SKLVDAQITDSIVC--EG--TILK--S---CSILHCVLG--VR--TRI--ESD--SVLEDTLVM--GADFFES--PEERIELRRG--GGTPL--GV
173  PP--SKLQDAQVTESIIG--EG--SLLK--A---CSIHHCVLG--VR--SRV--EDR--VVLQDTLVM--GSDYFES--SEERATLRQR--GGIPL--GV
174  PP--TKMVDCTITESMIS--EG--CILK--E---CRIHHSVLG--IR--ARV--EAG--CTIEDSLLM--GLDFYES--SAQRKAASQA--GKVPQ--GI
175  PP--SKLLNCDITESMIG--EG--CILK--N---CRIQHSVLG--VR--SRI--ESG--CVIEESLLM--GADYYQP--SVERQCSLEQ--GDIPV--GI
176  PP--SKLLDCRVTESIIG--EG--CILK--D---CRINNSVLG--LR--SRV--EAG--TVIEDTLIM--GADYYQS--LTERLSAQEQ--GQVTL--GI
177  PP--SKLLDCHVTQSIIG--EG--CILK--N---CRIENSVLG--VR--SRI--ESG--CIIQDSMIM--GADMYQP--FAERQSDCDH--RSVPL--GI
178  PP--TKVLNCNITESMIS--EG--CMIK--D---CRIHNSVLG--IR--SRI--ETD--CVVEDSLLM--GADYYES--LETRQSLLDQ--GKIPV--GI
179  PP--TKQLKCEVIQSMIS--EG--CVLK--D---CYIENSVIG--IR--SRI--DSG--CTIKNVLLM--GADYYQS--DFENEGDCSL--ENIPI--GI
180  PP--SKFVDSQITDSIIS--EG--SIIK--A---CSIHHSVLG--VR--SRV--ENN--VVLQDSLLM--GADFFES--QSERETLRAR--GGIPV--GV
181  PP--TKVFDSHITKSMIS--EG--CIIK--K---CRIHNSILG--IR--SRI--EMN--CHIEDTMIM--GADFYES--STVNSSYSSP--KEIPI--GI
```

```
182  PP--TKLLDTHVTESIIA--EG--CILK--Q---CRIDHSVLG--VR--SRI--EAG--CTIQDTLVM--GADFYEP--DAERHSSLGT--GGVAL--GI
183  PP--SKLLDCQVTESIIA--EG--CILK--E---CRIDHSVLG--VR--SRI--EAG--CNIEDSLIM--GSDFYEP--FAERQSGSDK--GGVPV--GI
184  PP--SKLLDCDIKESMIG--EG--CILK--N---CRIQHSVLG--VR--SRV--ESG--SIVEESLIM--GSDFYQP--SVERVCNLDK--GDIPL--GI
185  PP--SKLLDCDIKESMIG--EG--CILK--N---CRIQHSVLG--VR--SRV--ESG--SIVEESLIM--GSDFYQP--SVERVCNLDK--GDIPL--GI
186  PP--SKMLDCQITESIIA--EG--CILK--E---CRIDHSVLG--LR--SRV--ESG--SLVEDTMLM--GSDFYQP--FAERQYGLEK--GSVPI--GI
187  PP--TKVLNCNITESMIS--EG--CMIK--D---CRINNSVLG--IR--SRI--ESD--CVVEDSLLM--GADFYES--LDTRQSLLDQ--GKIPV--GI
188  PP--SKLVDAQITNSIVG--EG--SILK--S---CSIHHCVLG--VR--SRI--ESD--CVLQDTLVM--GADFFES--PDERAVLKER--GGIPL--GV
189  PP--AKIEKCHVKDAIIS--HG--CSLA--D---CCVENAIVG--LR--SQV--GKG--CKIERAMII--GADFYES--EDQKAKVIAS--GGVPV--GI
190  PP--SKLLDVQVSRSTIG--DG--CFIK--K---STISNSMIG--LR--TSI--SEG--CVIEDSMIM--GADYYEE--THECEDLP----DCTPI--GI
191  PP--AKVEKCHVKDAIIS--HG--CSLA--D---CSVEDAIIG--LR--SQI--GKG--CTIKHAMII--GADYYET--DEQKMALVEA--GGVPV--GI
192  PP--SKLLDAEVSKCTIG--DG--CFIK--K---SKLTNAMIG--LR--TNI--QED--CVIEDVMIM--GADYYEE--THECEDLP----GCTPI--GI
193  PP--ATVRNCKVTDAIIA--QG--SFVS--D---CTINNAVIG--IR--SII--GQN--CTIQDALVM--GADYYES--DDQRATLLKK--GGVPV--GI
194  PP--SKVMDCDVNMSIIG--DG--CVIK--AG--SKIHNSIIG--IR--SLI--GSD--CIIDSAMMM--GSDYYET--LEECEYVP----GCLPM--GV
195  PP--AKVERCHVKESIIS--HG--ASLA--D---CQVEESIIG--LR--SVV--NKG--CRIKRAMII--GADFYES--DEKKASLLAS--GEVPV--GI
196  PP--SKVQDCEIERSTIG--DG--CFIT--K---AKLKNVMVG--LR--STV--NAN--CDLEDTLVM--GADYYET--YDEAKTSALP--GGVPI--GI
197  PP--SKVLDADVSMSIIG--DG--CVIK--AG--SKIHNSIIG--IR--SLV--GSD--CIIDSAMMM--GADYYET--LEECEYVP----GCLPM--GV
198  PP--ATVRNCKVSDAIIA--QG--SFVA--D---SSISNAVIG--IR--SII--GSG--CTVQDALIM--GADYYQS--DEQRAALLAA--GDVPV--GI
199  PP--AKIERCHVKDSIIS--HG--AALA--D---CSVEESIVG--LR--SRV--EAG--TKIKRTMII--GADFYES--EEKRKAILAA--GGVPV--GI
200  PP--SKVQDCEIERSTIG--DG--CTIK--Q---AKLKNVMVG--LR--STV--NEG--CDLEDTLVM--GADYYES--LEECDPASLP--GCTPI--GI

                510       520       530       540       550       560       570       580       590
     ....|....|....|....|....|....|....|....|....|....|....|....|....|....|....|....|....|....|.
1    G--KN--CHIKRAIID--KN--ARIG--DN--VKI--INKDNV---QEAARE--TD--GYFIKS--G--IVTVI--KD--ALI--PSGIII
2    G--KN--CHIKRAIID--KN--ARIG--DN--VEI--INKDNV---QEAARE--TD--GYFIKS--G--IVTVI--KD--ALI--PSGIII
3    G--KN--CHIKRAIID--KN--ARIG--DN--VKI--INKDDV---QEAARE--TD--GYFIKS--G--IVTVI--KD--ALI--PSGIII
4    G--KN--CHIKRAIID--KN--ARIG--DN--VKI--INKDNV---QEAARE--TD--GYFIKS--G--IVTVI--KD--ALI--PSGIVI
5    G--KN--SHIKRAIID--KN--ARIG--DN--VKI--INSDNV---QEAARE--TD--GYFIKS--G--IVTVI--KD--ALI--PTGTVI
6    G--KN--SHIKRAIID--KN--ARIG--DN--VKI--INSDNV---QEAARE--TD--GYFIKS--G--IVTVI--KD--ALI--PTGTLI
7    G--RN--SHIKRAIVD--KN--ARIG--EN--VKI--INSDNV---QEAARE--TE--GYFIKS--G--IVTII--KD--ALI--PSGTVI
8    G--KN--SHIKRAIID--KN--ARIG--DD--VKI--INSDNV---QEAARE--TE--GYFIKS--G--IVTVI--KD--ALI--PSGTVI
9    G--RN--SHIKRAIID--KN--ARIG--EN--VKI--INSDNV---QEAARE--TD--GYFIKS--G--IVTVI--KD--ALI--PSGTVI
10   G--RN--SHIKRAIID--KN--ARIG--EN--VKI--INSDNV---QEAARE--TD--GYFIKS--G--IVTVI--KD--ALI--PSGTVI
11   G--KN--SHIKRAIID--KN--ARIG--DN--VKI--INGDNV---QEAARE--TD--GYFIKS--G--IVTVI--KD--ALI--PSGTVI
12   G--RN--SHIKRAIID--KN--ARIG--DN--VKI--INSDNV---QEAARE--TD--GYFIKS--G--IVTVI--KD--ALI--PSGTVI
13   G--KN--SHIKRAIID--KN--ARIG--DN--VKI--INSDNV---QEAARE--TD--GYFIKS--G--IVTVI--KD--ALL--PSGTII
14   G--EN--SHIKRAIID--KN--ARIG--DN--VKI--INTDNV---QEAARE--TD--GYFIKS--G--IVTVI--KD--ALI--PSGTVI
16   G--KN--SHIKRAIID--KN--ARIG--DD--VKI--INSDNV---QEAARE--TE--GYFIKS--G--IVTVI--KD--ALI--PSGTVI
17   G--KN--SHIKRAIVD--KN--ARIG--EN--VKI--INSDNV---QEAARE--TE--GYFIKS--G--IVTII--KD--ALI--PSGTVI
```

```
18   G--KN--SHIRRAIID--KN--ARIG--DN--VKI--INSDNV---QEAARE--TE--GYFIKS--G--IVTVI--KD--ALI--PSGTVI
19   G--RN--SHIKRAIVD--KN--ARIG--EN--VKI--INSDNV---QEAARE--TD--GYFIKS--G--IVTVI--KD--ALI--PSGTVI
20   G--RN--SHIKRAIID--KN--ARIG--ED--VKI--VNGDNV---QEAARE--TD--GYFIKS--G--IVTVI--KD--ALI--PSGTVI
21   G--RN--SHIKRAIID--KN--ARIG--EN--VKI--VNGDNV---QEAARE--TD--GYFIKS--G--IVTVI--KD--ALI--PSGTII
22   G--KN--SHIKRAIID--KN--ARIG--NN--VKI--VNRDSV---QEAARE--TD--GYFIKS--G--IDTII--KD--ALI--PSGTII
23   G--KN--SHIKRAIID--KD--ARIG--DN--VKI--VNSDSV---QEAARE--TD--GYFIKS--G--IVTII--KD--ALI--PSGTII
24   G--KN--SHIKRAIID--KN--ARIG--DN--VKI--IKSDNV---QETARE--TD--GYFIKS--G--IVTVI--KD--AWI--PSGTVI
25   G--RN--SHIKRAIID--KN--ARIG--ND--VKI--INNDNV---QEAARE--TE--GYFIKS--G--IVTII--KD--ALI--PSGTII
26   G--RN--SHIKRAIID--KN--ARIG--DN--VKI--INSDDV---QEAARE--TD--GYFIKS--G--IVTVI--KD--ALI--PSGTVI
27   G--KN--SHIKRAIID--KN--VRIG--EN--VKI--INSDNV---QEAARE--TD--GYFIKS--G--IVTVI--KD--ALI--PSSTII
28   G--KN--THIKRAIID--KN--ARIG--EN--VKI--VNGDNV---QEAARE--TD--GYFIKS--G--IVTVI--KD--ALI--PSGTMI
29   G--RN--SHVKRAIID--KN--ARIG--EN--VKI--LNSDNV---QEAARE--TD--GYFIKS--G--IVTVI--KD--ALI--PSGTVI
30   G--KN--SHIRRAIID--KN--ARIG--DD--VKI--INSDNV---QEAARE--TE--GYFIKS--G--IVTVI--KD--ALI--PSGTVI
31   G--KN--SHIKRAIVD--KN--ARIG--EN--VKI--INSDNV---QEAARE--TE--GYFIKS--G--IVTII--KD--ALI--PSGTVL
32   G--KN--SHIRKAIID--KN--ARIG--EN--VKI--INFDNV---QEAVRE--TE--GYFIKS--G--IVTVI--KD--ALI--PSGTII
33   G--KN--SCIRRAIID--KN--ARIG--DN--VKI--LNADNV---QEAAME--TD--GYFIKG--G--IVTVI--KD--ALL--PSGTVI
34   G--KN--SCIRRAIID--KN--ARIG--DN--VKI--LNADNV---QEAARE--TD--GYFIKG--G--IVTVI--KD--ALL--PSGTVI
36   G--KN--SHIRKAIID--KN--ARIG--DN--VKI--LNADNV---QEAARE--TD--GYFIKG--G--IVTVI--KD--ALL--PSGTVI
37   G--KN--CHIRRAIID--KN--ARIG--DN--VKI--INVDNV---QEAARE--TD--GYFIKS--G--IVTVI--KD--ALL--PSGTVI
38   G--KN--AHIRKAIID--KN--ARIG--EN--VKI--INVDNI---QEASRE--TD--GYFIKS--G--IVTVI--KD--ALI--PSGTVI
39   G--KN--CHIRRAIID--KN--ARIG--DN--VKI--INVDNV---QEAARE--TD--GYFIKS--G--IVTVI--KD--ALL--PSGTVI
41   G--KN--SHIKRAIID--KN--ARIG--DN--VKI--INVDNV---QEAARE--TD--GYFIKS--G--IVTVI--KD--ALL--PSGTVI
42   G--KN--SHIKRAIID--KN--ARIG--DN--VKI--INVDNV---QEAARE--TD--GYFIKS--G--IVTVI--KD--ALL--PSGTVI
43   G--KN--AHIRKAIID--KN--ARIG--EN--VKI--INFDNV---QEAVRE--TE--GYFIKS--G--IVTVI--KD--ALI--PSGTII
44   G--KN--THIKKAIID--KN--ARIG--EN--VKI--INVDDI---QEASRE--SD--GYFIKS--G--IVTVI--KD--ALI--PSGTVI
45   G--KN--SHIKRAIID--KN--ARIG--DN--VMI--INVDNV---QEAARE--TD--GYFIKS--G--IVTVI--KD--ALL--PSGTVI
46   G--KN--SHIKRAIID--KN--ARIG--DN--VMI--INVDNV---QEAARE--TD--GYFIKS--G--IVTVI--KD--ALL--PSGTVI
47   G--KN--AHIRKAIID--KN--ARIG--EN--VKI--INVDDI---QEASRE--SD--GYFIKS--G--IVTVI--KD--ALI--PSGTVI
48   G--KN--SHIKRAIID--KN--ARIG--DN--VMI--INVDNV---QEAARE--TD--GYFIKS--G--IVTVI--KD--ALL--PSGTVI
49   G--EN--TKIRKCIID--KN--AKIG--KN--VSI--INKDGV---QEADRP--EE--GFYIRS--G--IIIIL--EK--ATI--RDGTVI
50   G--PN--TKIQNCIID--KN--AKIG--KD--VVI--LNKEGV---EEADRS--AE--GFYIRS--G--ITVIM--KN--ATI--KDGTVI
51   G--EN--TRIKDCIID--KN--ARIG--KN--VVI--ANSEGV---QEADRS--SE--GFYMAS--G--ITVIS--KN--STI--PDGTVI
52   G--EN--TKIQECIID--KN--ARVG--KN--VII--ANSEGI---QEADRS--SD--GFYIRS--G--ITVIL--KN--SVI--KDGVVI
53   G--QN--TKIKNCIID--KN--AKIG--KN--VVI--ANADGV---EEGDRP--EE--GFHIRS--G--ITVVL--KN--ATI--RDGLHI
54   G--RD--TKIRKCIID--KN--AKIG--KN--VVI--MNKDDV---KEADRP--EE--GFYIRS--G--ITVVV--EK--ATI--KDGTVI
55   G--KD--TKIRKCIID--KN--AKIG--KN--VII--MNKGDV---QEADRP--EE--GFYIRS--G--ITVIV--EK--ATI--QDGTVI
56   G--EN--TKIRKCIID--KN--AKIG--KN--VSI--INKDGV---QEADRP--EE--GFYIRS--G--IIIIA--EK--ATI--RDGTVI
57   G--PN--TKIQKCIID--KN--AKIG--KD--VVI--LNKQGV---EEADRS--AE--GFYIRS--G--ITVIM--KN--ATI--KDGTVI
```

```
58   G--EN--TRIKECIID--KN--ARIG--KN--VVI--ANSEGV---QEADRS--SE--GFYIRS--G--ITVIL--KN--STI--PDGTVI
59   G--EN--TKIRKCIID--KN--AKIG--KN--VSI--INKDGV---QEADRP--EE--GFYIRS--G--IIIIS--EK--ATI--RDGTVI
60   G--PN--TKIQKCIID--KN--AKIG--KD--VVI--LNKQGV---EEADRS--AE--GFYIRS--G--ITVIM--KN--ATI--KDGTVI
61   G--EN--TKIKECIID--KN--ARVG--KN--VII--ANSEGI---QEADRS--SD--GFYIRS--G--ITVIL--KN--SII--KDGVVI
62   G--QN--TKIRNCIID--KN--AKIG--KN--VVI--ANAEGV---EEGDRP--EE--GFHIRS--G--ITVVL--KN--ATI--RDGLHI
63   G--RD--TKIRKCIID--KN--AKIG--KN--VMI--LNKDDV---KEADRP--EE--GFYIRS--G--ITVVV--EK--ATI--KDSTVI
64   G--RD--TKVRKCIID--KN--AKIG--KN--VII--MNKGDV---QEADRP--EE--GFYIRL--G--ITVIV--EK--ATI--QDGTVI
66   G--KN--TKIVNCIID--KN--ARIG--NN--VII--ANKDNV---QEADRP--SE--GFYIRS--G--ITVVL--KE--SVI--SNGTII
67   G--EN--TKIKDCIID--KN--ARIG--KN--VVI--ANSEGI---QEADRS--SE--GFYIRS--G--VTIVL--KN--SVI--EDGFII
68   G--EN--TRIRNCIID--KN--ARIG--RN--VII--ANTDGV---QEADRP--AE--GFYIRS--G--IVVVV--KN--ATI--EDGTVI
69   G--EN--TKIRNCIID--KN--ARIG--RN--VII--ANTDGV---QEADRP--ME--GFYIRS--G--IVVVA--NN--ATI--EDGTVI
70   G--RN--TKIRNCIID--KN--AKIG--KD--VII--MNKDGV---QEADRP--ED--GFYIRS--G--ITVIL--EK--ATI--EDGTVI
71   G--RN--TKIRNCIID--KN--AKIG--KD--VII--MNKDGV---QEADRP--ED--GFYIRS--G--ITVIL--EK--ATI--EDGTVI
72   G--EN--TKIKDCIID--KN--ARIG--KN--VVI--ANSEGI---QEADRS--SE--GFYIRS--G--VTIVL--KN--SVI--EDGFII
73   G--AN--TKIRNCIID--KN--AKIG--RN--VII--ANTDGV---QEADRA--KE--GFYIRS--G--ITVTL--KN--ATI--KDGTVI
74   G--RN--TKIRNCIID--KN--AKIG--KD--VII--ANKDGV---QEADRP--ED--GFYIRS--G--ITIIM--EK--ATI--EDGTVI
75   G--EN--TKIRNCIID--KN--AKIG--RN--VVI--ENIDGV---QEADRA--KE--GFYIRS--G--ITITL--KN--ATI--KDGTVI
76   G--RN--TKIRNCIID--KN--AKIG--KD--VII--ANKDGV---QEADRP--ED--GFYIRS--G--ITIIM--EK--ATI--EDGTIV
77   G--KN--TKIVNCIID--KN--ARIG--NS--VII--ANKDNV---QEADKP--TD--GFYIRS--G--ITVVL--KD--SVI--SNDTII
80   G--SN--SKVRKCIID--KN--ARIG--KD--VII--MNKDGV---QEADRP--ED--GFYIRS--G--ITIVM--EK--ATI--EDGTVI
81   G--KD--TKIMNCIID--KN--ARIG--KN--VII--ANKEGV---QEAERP--SE--GFYIRS--G--ITVVL--KN--SVI--KDGTII
82   G--RN--TKIRNCIID--KN--AKIG--KD--VII--MNKDGV---QEADRE--EE--GFYIRS--G--ITIIS--EK--ATI--EDGTVI
84   G--EN--TKIKECIID--KN--ARIG--KN--VVI--ANSEGI---QEADRS--ME--GFYIRS--G--VTVIL--KN--SVI--QDGTVI
87   G--EN--TKIRECIID--KN--ARIG--KN--VVI--ANSEGI---QEADRS--SE--GFYIRS--G--VTIIL--KN--SVI--QDGFVI
88   G--QE--TKIMNCIID--KN--ARIG--KN--VVI--ANKDHV---EEADRP--SE--GFYIRS--G--ITVVL--KN--SEI--KDGTII
89   G--RN--TKIKNCIID--KN--AKIG--KD--VVI--VNKDGV---QEADRP--EE--GFYIRS--G--ITIIM--EK--ATI--EDGTVI
90   G--QN--TKIRNCIID--KN--AKIG--RG--VVI--TNADGV---QEAERP--EE--GFYIRS--G--ITVIM--EN--ATI--NDGTII
91   G--QN--TRIRNCIID--KN--AKIG--RD--VVI--ANADGV---QEADRP--SE--GFYIRS--G--ITVIL--KN--ATI--NDGTII
92   G--KG--TKIMNCIID--KN--ARIG--KN--VVI--TNKDKV---EEADRP--SE--GFYIRS--G--ITVVL--KN--SVI--MDETII
94   G--EN--TRIKDCIID--KN--ARIG--KN--VVI--SNSEGI---QEADRS--LE--GFYIRS--G--ITIIL--KN--FTI--KDGFVI
95   G--RN--TKIRNCIID--KN--AKIG--KD--AVI--VNKDGV---QEADRP--DD--GFYIRS--G--ITIIL--EK--ATI--KDGTVI
96   G--QN--TKIRNCIID--KN--AKIG--KD--VII--TNADGV---QEADRP--SE--GFYIRS--G--ITAVL--KN--AAI--KDGTLI
97   G--RN--TKIRNCIID--KN--AKIG--KD--VII--TNKDGV---QEADRE--EK--GFYIRS--G--ITIIL--EK--ATI--EDGTVI
98   G--QN--TKIRNCIID--KN--AKIG--KD--VII--TNADGV---QEADRP--SE--GFYIRS--G--ITAVL--KN--ATI--KDGTII
99   G--EN--TTIQKCIID--KN--ARIG--KK--VVI--SNSEGV---DEADRT--SE--GFYIRS--G--ITVVL--KN--AII--ADGLVI
100  G--RN--TKIRNCIID--MN--ARIG--KN--VVI--TNSKGI---QEADHP--EE--GYYIRS--G--IVVIL--KN--ATI--NDGSVI
101  G--EN--TKISNCIID--MN--CQGW--KE--RLH--NKQRGRS--KSPDRP--GR--RILIRS--G--IVVVL--KN--ATI--KDGTVI
102  G--EN--TKIRNCIID--KN--ARIG--KN--VVI--MISENV---QEADRP--AE--GYYIRS--G--ITVVL--KN--AVI--LNGTKI
```

```
103 G--EN--AKISNCIID--MN--ARIG--RD--VII--ANSEGV---EEADRA--EE--GYYIRS--G--IVVIL--KN--ATI--KDGTVV
104 G--EN--TKIRNCIID--KN--ARIG--KN--VVI--MNSENV---QEADRP--SE--GYYIRS--G--ITVVL--KN--AVI--PDDTII
105 G--EN--TTIQNCIID--KN--ARIG--KN--VTI--ANSEGV---QEADRT--SE--GFHIRS--G--ITVVL--KN--SVI--ADGLVI
106 G--EN--TKIQNCIID--KN--ARIG--KN--VTI--SNSEGV---QEADRT--SE--GFYIRS--G--ITIVL--KN--SII--ADGLVI
107 G--EN--TKINNCIID--MN--ARVG--RN--VVI--TNSEGV---QESDRP--EE--GYYIRS--G--IVVIL--KN--ATI--KDGKVI
108 G--EN--TIIRNCIID--KN--ARIG--KN--VMI--MNSQNV---QEAERP--LE--GFYIRS--G--ITVVL--KN--AVI--PDGTVI
109 G--EN--TKIRNCIID--MN--ARIG--RN--VII--ANTQGV---QESDHP--EE--GYYIRS--G--IVVIL--KN--ATI--KDGTVI
110 G--EN--TKISNCIID--MN--ARVG--RN--VSI--TNTEGV---QEADRP--EL--GYYIRS--G--IVVIL--KN--ATI--KDGTVI
111 G--GN--TKIRNCIID--IN--ARIG--KN--VVI--TNSKGI---QEADHP--EE--GYYIKS--G--IVVIL--KN--ATI--KDGSVI
112 G--EN--TTIQKCIID--KN--ARIG--KN--VII--SNSEGV---VEADRT--SE--GFYIRT--G--VTVVL--KN--SII--ADGLVI
113 G--EN--TKISNCIID--MN--ARIG--RD--VVI--SNKEGV---QEADRP--EE--GYYIRS--G--IVVIQ--KN--ATI--KDGTVV
114 G--EN--TSIQNCIID--KN--ARIG--KN--VTI--ANTEGV---QESDRT--SE--GFHIRS--G--ITVVL--KN--SVI--ADGLVI
115 G--EN--TKISNCIID--MN--ARIG--RD--VVI--SNKEGV---QEADRP--EE--GYYIRS--G--IVVIQ--KN--ATI--KDGTVV
116 G--EN--TKIKDCIID--KN--ARIG--KN--ATI--SNVDGV---QEADRS--AE--GFYTRS--G--ITVIL--KN--STI--PDGFAI
117 G--EN--TKISNCIID--MN--ARIG--RD--VVI--SNKEGV---QEADRP--EE--GYYIRS--G--IVVIQ--KN--ATI--KDGTVV
118 G--EN--TSIQNCIID--KN--ARIG--KN--VTI--ANAEGV---QESDRA--SE--GFHIRS--G--ITVVL--KN--SVI--ADGLVI
119 G--EN--TKISNCIID--MN--ARIG--RD--VVI--SNKEGV---QEADRP--EE--GYYIRS--G--IVVIQ--KN--ATI--KDGTVV
120 G--EN--TKISNCIID--MN--ARIG--RD--VVI--SNKEGV---QEADRP--EE--GYYIRS--G--IVVIQ--KN--ATI--KDGTVV
121 G--QG--TTVKRAILD--KN--TRIG--SN--VTI--VNKDHV---EEADRP--EL--GFYIRN--G--IVVVV--KN--ASI--PDGTVI
122 G--NN--TTIRRAIVD--KN--ARIG--RH--VQI--INKDHV---QEAERE--ED--GFYIRG--G--ITVIL--KN--AVI--QDGTII
123 G--TD--TIIRRAIID--KN--ACIG--HD--VKI--INKDNV---QEAERE--NQ--GFYIRS--G--IVVVL--KG--AVI--ADGTII
124 G--KG--STIRRAIID--KN--ARIG--RN--VTI--VNKENI---EESNRE--DE--GFYIRN--G--IVVAI--KN--AII--PDGTVI
125 G--SN--TTIDHAIID--KN--ARIG--CN--VKI--INKDNV---SEAEKE--DQ--GFYIRS--N--IITIL--KD--AVI--PHDTVI
126 G--RG--TTVKRAILD--KN--VRIG--RD--VTI--VNKDRV---EEADRP--EL--GFYIRN--G--IVVVV--KN--ATI--ADGTVI
127 G--RG--TTVKRAILD--KN--ARIG--DN--VTI--VNKDNV---EEADRP--EL--GFYIRN--G--IVVVV--KN--ASI--PDHSVI
128 G--RG--TTVRRAILD--KN--VRIG--RN--VTI--VNKDGI---EEADRP--EL--GFYIRN--G--IVVVE--KN--ATI--ADGTVI
129 G--AD--TTIRRAIVD--KN--ARIG--SN--VTI--TNKEDV---EQAERE--EL--GFYIRS--G--IVTIL--KN--AVI--PDGTVI
130 G--EG--STVKRAILD--KN--TRIG--RN--VTI--INKDNV---EEADRP--EL--GFYIRN--G--IVVVC--KN--ATI--PDGMVI
131 G--PG--TTVKRAILD--KN--TRIG--SN--VSI--INKDHV---EEADRS--DL--GFYIRN--G--IVVVQ--KN--ATI--QDGTVI
132 G--KG--TTVKRAILD--KN--ARIG--SN--VTI--VNKDHV---EEADRP--EH--GFYIRN--G--IVVVV--KN--ASI--PDGTVI
133 G--ER--CVLQKAIID--KN--ARIG--ND--VRI--LNKERP---DSADHP--ER--GFYIRH--G--IVIVP--KD--TVI--PDGTVI
134 G--EG--STIKRAILD--KN--ARIG--DN--VVI--VNKDRV---EEADKP--DV--GFYIRN--G--IVVVV--KN--ATI--ANGTII
135 G--QG--TTVKRAILD--KN--TRIG--EN--VTI--INKDRI---EEADRA--DQ--GFYIRN--G--IVVVV--KN--ASI--LDGTII
136 G--AN--SVIRRAIVD--KN--ACIG--RD--VKI--INKDNV---EESNRE--DQ--GFYIRS--G--VVVII--KN--AVI--PDGTII
137 G--EG--STIRRAIVD--KN--ARIG--RN--VTI--VNKENI---DESNQE--ES--GFYIRN--G--IVVIL--KN--ATI--ADGTVI
138 G--TD--TIIRGAIID--KN--ARIG--HD--VKI--VNKDNV---QEAERE--NQ--GFYIRS--G--IVVVL--KN--AVI--PDGTII
139 G--EG--TTVKGAILD--KN--TRIG--NN--VTI--VNKDHV---EEADRA--DE--GFYIRN--G--IVVVV--KN--ATI--SDGTVI
140 G--AN--SHIVNAIVD--KN--ARIG--RN--VRI--LNKDHV---TEAQRE--EE--GIWISN--G--IVTII--KD--SVI--PDNTII
```

```
141 G--AN--SHIVNAIVD--KN--ARIG--RN--VRI--LNKDHV---SEAQRE--EE--GIWISN--G--IVTII--KD--SVI--PDNTVI
142 G--QG--TTVKGAILD--KN--TRIG--NN--VTI--VNKDHV---EEADRA--DE--GFYIRN--G--IVVVV--KN--ATI--SDGTVI
143 G--QG--TTVKRAILD--KN--ARIG--SN--VTI--VNKDHV---EEADRS--DQ--GFYIRN--G--IVVVV--KN--ATI--QDGTVI
144 G--PD--TIIRRAIID--KN--ARIG--HD--VKI--INKDNV---QEADRE--SQ--GFYIRS--G--IVVVL--KN--AVI--TDGTII
145 G--QG--TTVKRAILD--KN--ARIG--EN--VAI--VNKDNV---EEADRP--EE--GFYIRN--G--IVVVV--KN--ATI--SDGTII
146 G--EG--TTVKRAILD--KN--TRIG--DN--VVI--INKDRV---EEADKP--EL--GFYIRN--G--IVVVV--KN--ATI--ANGTVI
147 G--QG--TTVKRAILD--KN--ARIG--DN--VTI--VNKDNV---EEADRA--DQ--GFYIRN--G--IVVIV--KN--ATI--PDGTII
148 G--EG--TTVKRAILD--KN--TRIG--DN--VVI--INKDRV---EEADKP--EL--GFYIRN--G--IVVVV--KN--ATI--ANGTVI
149 G--VG--STIKRAILD--KN--ARIG--DN--VVI--VNKDRV---EEADKP--EL--GFYIRN--G--IVVVV--KN--ATI--ANGTII
150 G--EG--TTVKGAILD--KN--ARIG--KN--VTI--VNKDRV---EEADRP--DQ--GFYIRN--G--IIVVV--KN--ASI--ADDTVI
151 G--AG--TTIRRAIID--KN--ARIG--RN--VLI--INKDRI---EEAERE--DE--GFLIRS--G--IVVVI--KN--ATI--PDGTVI
152 G--EG--TTVKRAILD--KN--TRIG--DN--VVI--INKDRV---EEADKP--EL--GFYIRN--G--IVVVV--KN--ATI--ANGTVI
153 G--SG--TTIRRAIID--KN--ARIG--KN--VMI--VNKENV---QEANRE--EL--GFYIRN--G--IVVVI--KN--VTI--ADGTVI
154 G--EG--STIRRAIVD--KN--ARIG--RN--VTI--VNKENI---DESNQE--ES--GFYIRN--G--IVVIL--KN--ATI--ADGTVI
155 G--SG--STIRRAIVD--KN--AHIG--QN--VQI--VNKDHV---EEADRE--DL--GFMIRS--G--IVVVV--KG--AVI--PDNTVI
156 G--AD--TIVRRAIVD--KN--ACIG--RN--VKI--VNKDHV---EEANRE--SE--GFYIRN--G--IVVVL--KN--AVI--PDNTVI
157 G--SG--STIRRAIVD--KN--AHIG--QN--VQI--VNKDHV---EEADRE--DL--GFMIRS--G--IVVVV--KG--AVI--PDNTVI
158 G--KG--TTVKRAILD--KN--TRIG--SG--VSI--INKDNV---EEADRS--DQ--GFYIRN--G--IVVVQ--KN--ATI--ADGTVI
159 G--PG--TTVKRAILD--KN--TRIG--SN--VSI--INKDHV---EEADRS--DL--GFYIRN--G--IVVVQ--KN--ATI--QDGTVI
160 G--EN--TVIRKAIVD--KN--ARIG--KN--VKI--VNKAQV---EEANHE--DE--GFYIRS--G--IVVIL--KN--AII--PDGTEI
161 G--EN--TKISRAIID--KN--ARIG--RN--VKI--VNKDNV---EESNQE--EH--GFYIRS--G--IVVVL--KN--AEI--PDNTII
162 G--AE--TKIRGAIID--KN--ARIG--CN--VQI--INKDNV---EEAQRE--EE--GFIIRS--G--IVVVL--KN--ATI--PDGTVI
163 G--PD--TIIRRAIID--KN--ARIG--HD--VKI--INKDNV---QEADRE--SQ--GFYIRS--G--IVVVL--KN--AVI--TDGTII
164 G--SN--TTIDHAIID--KN--ARIG--CN--VKI--INKDNV---SEAEKE--DQ--GFYIRS--N--IITVV--KD--AVI--PHDTVI
165 G--KG--STIRRAIVD--KN--ARIG--QN--VTI--VNKENI---EESNRE--DD--GFYIRN--G--IVVVI--KN--AVI--PDGTVI
166 G--EG--TTIRRAIID--KN--ARIG--KN--VMI--VNKENV---EESNRE--EL--GYYIRS--G--ITVVL--KN--AVI--PDGTVI
167 G--EG--TTVKRAILD--KN--TRIG--DN--VVI--INKDRV---EEADKP--EL--GFYIRN--G--IVVVV--KN--ATI--ANGTVI
168 G--QG--TTVKRAILD--KN--ARIG--DN--VTI--VNKDNV---EEADRA--DQ--GFYIRN--G--IVVIV--KN--ATI--PDGTII
169 G--RG--TTVKKAILD--KN--VRIG--SN--VSI--INKDNV---EEADRA--EQ--GFYIRG--G--IVVIT--KN--ASI--PDGMVI
170 G--KG--TTVKRAILD--KN--ARIG--SN--VTI--VNKDHV---EEADRP--EH--GFYIRN--G--IVVVV--KN--ASI--PDGTVI
171 G--SG--STIRRAIVD--KN--ARIG--SN--VLI--VNKDRV---EEANRE--DL--GFYVRS--G--IVVIF--KN--ATI--PDGTVI
172 G--EG--TTVKRAILD--KN--TRIG--DN--VVI--INKDRV---DEADKP--EL--GFYIRN--G--IVVVV--KN--ATI--ANGTVI
173 G--SG--TTVRGAILD--KN--VRIG--RD--VTI--VNKDRV---EEADRP--EL--NFYIRN--G--IVVVV--KN--GTI--ADGTVI
174 G--AG--TTIRRAIID--KN--AHIG--KN--VLI--INKDRI---EEADRE--DQ--GFLIRN--G--IVVVM--KN--ATI--PDGTVI
175 G--TN--TIIRRAIID--KN--ARIG--HD--VKI--INKDNV---QEAERE--KQ--GFFIRS--G--IVVVL--KN--AVI--PDGTII
176 G--KD--TVIRRAIID--KN--ACIG--NN--VKI--FNKDRV---EEANCE--SE--GFYIRN--G--IVVVL--KN--AVI--PHGAVI
177 G--SN--TIIRRAIID--KN--THIG--CD--VQI--VNKDNV---QEAERE--SQ--GFYIRS--G--IVVVL--KN--AVI--PDGTII
178 G--KG--STIRRAIVD--KN--ARIG--QN--VTI--VNKENI---EESNRE--DD--GFYIRN--G--IVVVI--KN--AVI--PDGTVI
```

```
179  G--SN--TTIDHAIID--KN--ARIG--CN--VKI--INKDNV---SEAEKE--DQ--GFYIRS--N--IITVV--KD--AVI--PHDTVI
180  G--EG--TTVKRAILD--KN--ARIG--KN--VTI--VNKDHV---EEADRP--EH--GFYIRN--G--IVVVV--KN--ASI--ADDTVI
181  G--KN--SLIKHAIID--KN--ARIG--EN--VII--LNKNDI---QESSRE--DE--GFYICD--G--IVVII--KN--AVI--QSGTVI
182  G--AD--TTIRRAIVD--KN--ARIG--RN--VQI--INKDRV---EEANRE--NQ--GFYIRS--G--IIVVL--KN--ATI--PDGTII
183  G--AQ--TRIRRAIVD--KN--ARIG--RH--VQI--INKDRV---EEAERE--DQ--GFYIRS--G--IVVVL--KN--AII--SDGTII
184  G--TD--TIIRRAIID--KN--ARIG--HN--VRI--INKDNV---QEAERE--KQ--GFYIRS--G--IVVVL--KN--AVI--PDGTII
185  G--TD--TIIRRAIID--KN--ARIG--HN--VRI--INKDNV---QEAERE--KQ--GFYIRS--G--IVVVL--KN--AVI--PDGTII
186  G--NN--TTIRRAIVD--KN--ARIG--RH--VQI--INKDHV---QEAERE--ED--GFYIRG--G--ITVIL--KN--AVI--PDGTII
187  G--KG--STIRRAIVD--KN--ARIG--TN--VNI--VNKENI---EESNRE--DD--GFYIRN--G--IVVVI--KN--AVI--PDGTVI
188  G--KG--TTVRRAILD--KN--TRIG--SG--VSI--INKDNV---EEADRS--DQ--GFYIRN--G--IVVVQ--KN--ATI--ADGTVI
189  G--EG--CTITNAIID--KN--ARIG--KN--CII--TNASGI---DDLEDE--EN--GVYIRS--G--IVTIL--RN--ATI--PDGTVI
190  G--AG--TVIRRAIVD--KN--ARIG--MD--CQL--INKDNV---QEANEE--EK--GYIIKD--G--IIVIV--KD--SYI--PNGTII
191  G--EG--CSISNAIID--KN--ARIG--KN--CII--TNAAGV---EDLEDE--EN--GIYIRS--G--IVTIL--RN--ATI--PDGTVI
192  G--AG--TTIKRAIID--KN--ARIG--MD--CQI--INKDNV---QEANHE--DK--GYIIKD--G--IVVIC--KD--AII--PNGTVI
193  G--AN--SVITNAIID--KN--ARVG--KN--VKI--VNKEGV---TEGTRE--AE--GIYIRS--G--IVVID--KG--ALV--PDNTTI
194  G--DG--SIIRRAIVD--KN--ARIG--PK--CQI--INKDGV---KEANRE--DQ--GFVIKD--G--IVVVI--KD--SHI--PAGTII
195  G--EG--TIIENAIID--KN--ARVG--KN--CVI--TNAAGV---EDLADE--ER--GVFIRN--G--IITIL--RN--CTI--PDGTII
196  G--AG--TKIRKAIID--KN--ARIG--EN--CQI--LNEAGV---MDKDCE--NE--GYIIRD--G--IIVVI--KD--AVI--KPGTVI
197  G--DG--SVVRKAIID--KN--ARIG--PK--CQI--INKDGV---KEANRE--EQ--GFVIKD--G--IVVVI--KD--SCI--PAGTII
198  G--AN--SIISNAIID--KN--ARVG--KN--VRI--VNKDGV---SEGTRE--SE--GIYIRS--G--IVVID--KG--AKV--PDNATI
199  G--EN--TIIENAIID--KN--ARVG--KN--CVI--TNKDNI---EDLADE--ER--GVFIRN--G--IVTIL--RN--CTI--PDGTVI
200  G--AG--TKIRKAIID--KN--ARIG--EN--CQI--LNEAGV---MDKDCE--SE--GYIIRD--G--IIVVI--KD--AVI--KAGTVI
```
