# Supplementary material for: Resurrecting the Regulatory Properties of the Ostreococcus tauri ADP-Glucose Pyrophosphorylase Large Subunit
Source: Front Plant Sci. 2018 Oct 30;9:1564. doi: 10.3389/fpls.2018.01564 (PMC6218581; doi:10.3389/fpls.2018.01564)
Supplement: Supplementary file 2 [file Table_1.pdf]

Table S1. Protein sequences coding for ADP-Glc PPases from oxygenic photosynthetic organisms used to generate sequence logos presented in Figure 3. Numbers correspond to protein sequences used for the alignment shown in Figure S1. Color code: red, S subunits from plants; blue, L subunits from plants; green, cyanobacteria; orange, S subunits from green algae; purple, L subunits from green algae. Sequences were obtained from Kuhn et al. (2013).

Number	Accession	Annotation	Organism	Taxonomy
1	62738704	Chain A, Crystal Structure Of Potato Tuber Adp-Glucose Pyrophosphorylase	*Solanum tuberosum*	dicot
2	27819107	ADP-glucose pyrophosphorylase small subunit	Solanum tuberosum	dicot
3	77416911	ADP-glucose pyrophosphorylase small subunit-like	Solanum tuberosum	dicot
4	556351	ADP-glucose pyrophosphorylase small subunit	Solanum tuberosum	dicot
5	15238933	glucose-1-phosphate adenylyltransferase small subunit	*Arabidopsis thaliana*	dicot
6	297791999	hypothetical protein ARALYDRAFT_494882 agps	*Arabidopsis lyrata* subsp. lyrata	dicot
7	357495273	Glucose-1-phosphate adenylyltransferase	*Medicago truncatula*	dicot
8	357462397	Glucose-1-phosphate adenylyltransferase	*Medicago truncatula*	dicot
9	356552274	PREDICTED: glucose-1-phosphate adenylyltransferase small subunit, isoform 1	*Glycine max*	dicot
10	356501687	PREDICTED: glucose-1-phosphate adenylyltransferase small subunit, isoform 2	*Glycine max*	dicot
11	224131934	predicted protein	*Populus trichocarpa*	dicot
12	255567204	glucose-1-phosphate adenylyltransferase, putative	*Ricinus communis*	dicot
13	225447450	PREDICTED: hypothetical protein	*Vitis vinifera*	dicot
14	13487709	ADP-glucose pyrophosphorylase small subunit	*Brassica rapa* subsp. pekinensis	dicot
16	1237080	ADP-glucose pyrophosphorylase	*Pisum sativum*	dicot
17	1237082	ADP-glucose pyrophosphorylase	*Pisum sativum*	dicot
18	13487787	ADP-glucose pyrophosphorylase small subunit CagpS1	*Cicer arietinum*	dicot
19	16950559	ADP-glucose pyrophosphorylase small subunit CagpS2	*Cicer arietinum*	dicot
20	2642636	ADP-glucose pyrophosphorylase small subunit	*Citrullus lanatus* subsp. vulgaris	dicot
21	2625084	ADP-glucose pyrophosphorylase small subunit	*Cucumis melo*	dicot

22	5917789	ADP-glucose pyrophosphorylase small subunit	*Citrus unshiu*	dicot
23	111660950	ADP-glucose pyrophosphorylase small subunit	*Citrus sinensis*	dicot
24	41350641	ADP-glucose pyrophosphorylase small subunit	*Fragaria* x ananassa	dicot
25	45505207	ADP-glucose pyrophosphorylase small subunit	*Ipomoea batatas*	dicot
26	45505205	ADP-glucose pyrophosphorylase small subunit	*Ipomoea batatas*	dicot
27	7671232	ADP-glucose pyrophosphorylase	*Perilla frutescens*	dicot
28	7671230	ADP-glucose pyrophosphorylase catalytic subunit	*Perilla frutescens*	dicot
29	29421116	ADP-glucose pyrophosphorylase small subunit PvAGPS1	*Phaseolus vulgaris*	dicot
30	440595	ADP-glucose pyrophosphorylase	*Vicia faba* var. minor	dicot
31	440593	ADP-glucose pyrophosphorylase	*Vicia faba* var. minor	dicot
32	162462257	ADP-glucose pyrophosphorylase small subunit	*Zea mays*	monocot
33	162461970	LOC541902	*Zea mays*	monocot
34	73747074	ADP-glucose pyrophosphorylase small subunit	*Zea mays*	monocot
36	14582768	ADP-glucose pyrophosphorylase small subunit	*Zea mays*	monocot
37	115476014	Os08g0345800	*Oryza sativa* Japonica Group	monocot
38	115478426	Os09g0298200	*Oryza sativa* Japonica Group	monocot
39	125987830	GLGS_ORYSJ	*Oryza sativa* Japonica Group	monocot
41	357145854	PREDICTED: glucose-1-phosphate adenylyltransferase small subunit, isoform 2	*Brachypodium distachyon*	monocot
42	357145851	PREDICTED: glucose-1-phosphate adenylyltransferase small subunit, isoform 1	*Brachypodium distachyon*	monocot
43	242048788	hypothetical protein SORBIDRAFT_02g020410	*Sorghum bicolor*	monocot
44	27464770	ADP-glucose pyrophosphorylase small subunit	*Hordeum vulgare* subsp. vulgare	monocot
45	51556842	ADP-glucose pyrophosphorylase small subunit a	*Hordeum vulgare*	monocot
46	1707940	GLGS_HORVU	*Hordeum vulgare*	monocot
47	52430025	ADP-glucose pyrophosphorylase small subunit	*Triticum aestivum*	monocot
48	21687	ADP-glucose pyrophosphorylase	*Triticum aestivum*	monocot
49	232166	GLGL1_SOLTU	*Solanum tuberosum*	dicot

50	1707929	GLGL2_SOLTU	*Solanum tuberosum*	dicot
51	1707932	GLGL3_SOLTU	*Solanum tuberosum*	dicot
52	14916987	GLGL1_ARATH	*Arabidopsis thaliana*	dicot
53	12644324	GLGL2_ARATH	*Arabidopsis thaliana*	dicot
54	17433716	GLGL3_ARATH	*Arabidopsis thaliana*	dicot
55	11386853	GLGL4_ARATH	*Arabidopsis thaliana*	dicot
56	1947084	ADP-glucose pyrophosphorylase large subunit agpl1	*Solanum lycopersicum*	dicot
57	1840114	ADP-glucose pyrophosphorylase large subunit agpl2	*Solanum lycopersicum*	dicot
58	1840116	ADP-glucose pyrophosphorylase large subunit agpl3	*Solanum lycopersicum*	dicot
59	1778434	ADP-glucose pyrophosphorylase large subunit agp-s1	*Solanum lycopersicum*	dicot
60	1778436	ADP-glucose pyrophosphorylase large subunit agp-s2	*Solanum lycopersicum*	dicot
61	297812109	hypothetical protein ARALYDRAFT_488807 agpl1	*Arabidopsis lyrata* subsp. lyrata	dicot
62	297845724	hypothetical protein ARALYDRAFT_472972 agpl2	*Arabidopsis lyrata* subsp. lyrata	dicot
63	297797902	hypothetical protein ARALYDRAFT_490693 agpl3	*Arabidopsis lyrata* subsp. lyrata	dicot
64	297821353	predicted protein agpl4	*Arabidopsis lyrata* subsp. lyrata	dicot
66	356571037	PREDICTED: glucose-1-phosphate adenylyltransferase large subunit 2	*Glycine max*	dicot
67	356563435	PREDICTED: glucose-1-phosphate adenylyltransferase large subunit 1	*Glycine max*	dicot
68	356562361	PREDICTED: glucose-1-phosphate adenylyltransferase large subunit	*Glycine max*	dicot
69	356553863	PREDICTED: glucose-1-phosphate adenylyltransferase large subunit	*Glycine max*	dicot
70	356545193	PREDICTED: glucose-1-phosphate adenylyltransferase large subunit 1-like	*Glycine max*	dicot
71	356538761	PREDICTED: glucose-1-phosphate adenylyltransferase large subunit 1-like	*Glycine max*	dicot
72	356521967	PREDICTED: glucose-1-phosphate adenylyltransferase large subunit 1	*Glycine max*	dicot
73	356518710	PREDICTED: glucose-1-phosphate adenylyltransferase large subunit	*Glycine max*	dicot
74	356517038	PREDICTED: glucose-1-phosphate adenylyltransferase large subunit 1-like	*Glycine max*	dicot
75	356509672	PREDICTED: glucose-1-phosphate adenylyltransferase large subunit	*Glycine max*	dicot
76	356508352	PREDICTED: glucose-1-phosphate adenylyltransferase large subunit 1-like	*Glycine max*	dicot

77	356503982	PREDICTED: glucose-1-phosphate adenylyltransferase large subunit 2	*Glycine max*	dicot
80	357473317	Glucose-1-phosphate adenylyltransferase	*Medicago truncatula*	dicot
81	224128113	predicted protein	*Populus trichocarpa*	dicot
82	224103389	predicted protein	*Populus trichocarpa*	dicot
84	224100249	predicted protein	*Populus trichocarpa*	dicot
87	255552303	glucose-1-phosphate adenylyltransferase, putative	*Ricinus communis*	dicot
88	255548169	glucose-1-phosphate adenylyltransferase, putative	*Ricinus communis*	dicot
89	255543725	glucose-1-phosphate adenylyltransferase, putative	*Ricinus communis*	dicot
90	255538708	glucose-1-phosphate adenylyltransferase, putative	*Ricinus communis*	dicot
91	225458219	PREDICTED: hypothetical protein	*Vitis vinifera*	dicot
92	225437808	PREDICTED: hypothetical protein	*Vitis vinifera*	dicot
94	225432564	PREDICTED: hypothetical protein	*Vitis vinifera*	dicot
95	225428422	PREDICTED: hypothetical protein	*Vitis vinifera*	dicot
96	224085694	predicted protein	*Populus trichocarpa*	dicot
97	224080375	predicted protein	*Populus trichocarpa*	dicot
98	224062107	predicted protein	*Populus trichocarpa*	dicot
99	162460455	plastid ADP-glucose pyrophosphorylase large subunit	*Zea mays*	monocot
100	189027076	glucose-1-phosphate adenylyltransferase large subunit 1	*Zea mays*	monocot
101	162458350	glucose-1-phosphate adenylyltransferase large subunit 2	*Zea mays*	monocot
102	162463875	putative glucose-1-phosphate adenylyltransferase large subunit 3 precursor	*Zea mays*	monocot
103	357132398	PREDICTED: glucose-1-phosphate adenylyltransferase large subunit	*Brachypodium distachyon*	monocot
104	357119087	PREDICTED: glucose-1-phosphate adenylyltransferase large subunit 2	*Brachypodium distachyon*	monocot
105	357116651	PREDICTED: glucose-1-phosphate adenylyltransferase large subunit 1	*Brachypodium distachyon*	monocot
106	115455167	Os03g0735000	*Oryza sativa* Japonica Group	monocot
107	297604962	Os05g0580000	*Oryza sativa* Japonica Group	monocot
108	115471355	Os07g0243200	*Oryza sativa* Japonica Group	monocot

109	115438749	Os01g0633100	*Oryza sativa* Japonica Group	monocot
110	242088961	hypothetical protein SORBIDRAFT_09g029610	*Sorghum bicolor*	monocot
111	242053733	hypothetical protein SORBIDRAFT_03g028850	*Sorghum bicolor*	monocot
112	242033053	hypothetical protein SORBIDRAFT_01g008940	*Sorghum bicolor*	monocot
113	1279513	glucose-1-phosphate adenylyltransferase	*Hordeum vulgare* subsp. vulgare	monocot
114	2105137	ADP-glucose pyrophosphorylase large subunit	*Hordeum vulgare* subsp. vulgare	monocot
115	445623	ADP glucose pyrophosphorylase : SUBUNIT=L	*Hordeum vulgare*	monocot
116	22347636	ADP-glucose pyrophosphorylase large subunit	*Oncidium Goldiana*	monocot
117	32812836	ADP-glucose pyrophosphorylase large subunit	*Triticum aestivum*	monocot
118	89277026	plastid ADP-glucose pyrophosphorylase large subunit	*Triticum aestivum*	monocot
119	1707930	GLGL2_WHEAT	*Triticum aestivum*	monocot
120	121293	GLGL3_WHEAT	*Triticum aestivum*	monocot
121	87124328	ADP-glucose pyrophosphorylase	*Synechococcus* sp. RS9917	cyanobacteria
122	284051940	glucose-1-phosphate adenylyltransferase	*Arthrospira platensis* str. Paraca	cyanobacteria
123	119509469	glucose-1-phosphate adenylyltransferase	*Nodularia spumigena* CCY9414	cyanobacteria
124	126660345	glucose-1-phosphate adenylyltransferase	*Cyanothece* sp. CCY0110	cyanobacteria
125	126658160	glucose-1-phosphate adenylyltransferase	*Cyanothece* sp. CCY0110	cyanobacteria
126	318041355	glucose-1-phosphate adenylyltransferase	*Synechococcus* sp. CB0101	cyanobacteria
127	317969822	glucose-1-phosphate adenylyltransferase	*Synechococcus* sp. CB0205	cyanobacteria
128	87302823	glucose-1-phosphate adenylyltransferase	*Synechococcus* sp. WH 5701	cyanobacteria
129	119490200	glucose-1-phosphate adenylyltransferase	*Lyngbya* sp. PCC 8106	cyanobacteria
130	116074714	glucose-1-phosphate adenylyltransferase	*Synechococcus* sp. RS9916	cyanobacteria
131	116070673	glucose-1-phosphate adenylyltransferase	*Synechococcus* sp. BL107	cyanobacteria
132	88808518	glucose-1-phosphate adenylyltransferase	*Synechococcus* sp. WH 7805	cyanobacteria
133	37523829	glucose-1-phosphate adenylyltransferase	*Gloeobacter violaceus* PCC 7421	cyanobacteria
134	33861326	glucose-1-phosphate adenylyltransferase	*Prochlorococcus marinus* str. CCMP1986	cyanobacteria

135	33240292	glucose-1-phosphate adenylyltransferase	*Prochlorococcus marinus* str. CCMP1375	cyanobacteria
136	22298830	glucose-1-phosphate adenylyltransferase	*Thermosynechococcus elongatus* BP-1	cyanobacteria
137	218248785	glucose-1-phosphate adenylyltransferase	*Cyanothece* sp. PCC 8801	cyanobacteria
138	186686123	glucose-1-phosphate adenylyltransferase	*Nostoc punctiforme* PCC 73102	cyanobacteria
139	124023387	glucose-1-phosphate adenylyltransferase	*Prochlorococcus marinus* str. MIT 9303	cyanobacteria
140	86608545	glucose-1-phosphate adenylyltransferase	*Synechococcus* sp. JA-2-3B'a(2-13)	cyanobacteria
141	86606226	glucose-1-phosphate adenylyltransferase	*Synechococcus* sp. JA-3-3Ab	cyanobacteria
142	33862839	glucose-1-phosphate adenylyltransferase	*Prochlorococcus marinus* str. MIT 9313	cyanobacteria
143	33865652	glucose-1-phosphate adenylyltransferase	*Synechococcus* sp. WH 8102	cyanobacteria
144	17232137	glucose-1-phosphate adenylyltransferase	*Anabaena* sp. PCC 7120	cyanobacteria
145	159903534	glucose-1-phosphate adenylyltransferase	*Prochlorococcus marinus* str. MIT 9211	cyanobacteria
146	126696167	glucose-1-phosphate adenylyltransferase	*Prochlorococcus marinus* str. MIT 9301	cyanobacteria
147	124025514	glucose-1-phosphate adenylyltransferase	*Prochlorococcus marinus* str. NATL1A	cyanobacteria
148	123968364	glucose-1-phosphate adenylyltransferase	*Prochlorococcus marinus* str. AS9601	cyanobacteria
149	123966049	glucose-1-phosphate adenylyltransferase	*Prochlorococcus marinus* str. MIT 9515	cyanobacteria
150	113954397	glucose-1-phosphate adenylyltransferase	*Synechococcus* sp. CC9311	cyanobacteria
151	218437477	glucose-1-phosphate adenylyltransferase	*Cyanothece* sp. PCC 7424	cyanobacteria
152	78779161	glucose-1-phosphate adenylyltransferase	*Prochlorococcus marinus* str. MIT 9312	cyanobacteria
153	16332282	glucose-1-phosphate adenylyltransferase	*Synechocystis* sp. PCC 6803	cyanobacteria
154	257061844	glucose-1-phosphate adenylyltransferase	*Cyanothece* sp. PCC 8802	cyanobacteria
155	56750930	glucose-1-phosphate adenylyltransferase	*Synechococcus elongatus* PCC 6301	cyanobacteria
156	220910118	glucose-1-phosphate adenylyltransferase	*Cyanothece* sp. PCC 7425	cyanobacteria
157	81299414	glucose-1-phosphate adenylyltransferase	*Synechococcus elongatus* PCC 7942	cyanobacteria
158	78212786	glucose-1-phosphate adenylyltransferase	*Synechococcus* sp. CC9605	cyanobacteria
159	78184800	glucose-1-phosphate adenylyltransferase	*Synechococcus* sp. CC9902	cyanobacteria
160	158335435	glucose-1-phosphate adenylyltransferase	*Acaryochloris marina* MBIC11017	cyanobacteria

161	158335089	glucose-1-phosphate adenylyltransferase	*Acaryochloris marina* MBIC11017	cyanobacteria
162	113477795	glucose-1-phosphate adenylyltransferase	*Trichodesmium erythraeum* IMS101	cyanobacteria
163	75908241	glucose-1-phosphate adenylyltransferase	*Anabaena variabilis* ATCC 29413	cyanobacteria
164	172037571	glucose-1-phosphate adenylyltransferase	*Cyanothece* sp. ATCC 51142	cyanobacteria
165	172035903	glucose-1-phosphate adenylyltransferase	*Cyanothece* sp. ATCC 51142	cyanobacteria
166	170076729	glucose-1-phosphate adenylyltransferase	*Synechococcus* sp. PCC 7002	cyanobacteria
167	157413198	glucose-1-phosphate adenylyltransferase	*Prochlorococcus marinus* str. MIT 9215	cyanobacteria
168	72382015	glucose-1-phosphate adenylyltransferase	*Prochlorococcus marinus* str. NATL2A	cyanobacteria
169	148242352	glucose-1-phosphate adenylyltransferase	*Synechococcus* sp. RCC307	cyanobacteria
170	148239634	glucose-1-phosphate adenylyltransferase	*Synechococcus* sp. WH 7803	cyanobacteria
171	166365546	glucose-1-phosphate adenylyltransferase	*Microcystis aeruginosa* NIES-843	cyanobacteria
172	254526910	glucose-1-phosphate adenylyltransferase	*Prochlorococcus marinus* str. MIT 9202	cyanobacteria
173	254432095	glucose-1-phosphate adenylyltransferase	*Cyanobium* sp. PCC 7001	cyanobacteria
174	307151922	glucose-1-phosphate adenylyltransferase	*Cyanothece* sp. PCC 7822	cyanobacteria
175	298492804	glucose-1-phosphate adenylyltransferase	*Nostoc azollae* 0708	cyanobacteria
176	332709240	glucose-1-phosphate adenylyltransferase	*Lyngbya majuscula* 3L	cyanobacteria
177	354565610	glucose-1-phosphate adenylyltransferase	*Fischerella* sp. JSC-11	cyanobacteria
178	354556049	glucose-1-phosphate adenylyltransferase	*Cyanothece* sp. ATCC 51472	cyanobacteria
179	354553549	glucose-1-phosphate adenylyltransferase	*Cyanothece* sp. ATCC 51472	cyanobacteria
180	352094384	glucose-1-phosphate adenylyltransferase	*Synechococcus* sp. WH 8016	cyanobacteria
181	284929352	glucose-1-phosphate adenylyltransferase	cyanobacterium UCYN-A	cyanobacteria
182	334120832	glucose-1-phosphate adenylyltransferase	*Microcoleus vaginatus* FGP-2	cyanobacteria
183	300865383	glucose-1-phosphate adenylyltransferase	*Oscillatoria* sp. PCC 6506	cyanobacteria
184	282899378	glucose-1-phosphate adenylyltransferase	*Cylindrospermopsis raciborskii* CS-505	cyanobacteria
185	282895605	glucose-1-phosphate adenylyltransferase	*Raphidiopsis brookii* D9	cyanobacteria
186	209527099	glucose-1-phosphate adenylyltransferase	*Arthrospira maxima* CS-328	cyanobacteria

187	67924676	glucose-1-phosphate adenylyltransferase	*Crocosphaera watsonii* WH 8501	cyanobacteria
188	260436638	glucose-1-phosphate adenylyltransferase	*Synechococcus* sp. WH 8109	cyanobacteria
190	303271247	adp-glucose pyrophosphorylase	*Micromonas pusilla* CCMP1545	green alga
192	255080070	adp-glucose pyrophosphorylase	*Micromonas* sp. RCC299	green alga
194	159467349	ADP-glucose pyrophosphorylase small subunit	*Chlamydomonas reinhardtii*	green alga
196	308806175	AGPSU1 (ISS)	*Ostreococcus tauri*	green alga
197	302849075	hypothetical protein VOLCADRAFT_76956	*Volvox carteri* f. nagariensis	green alga
200	145349062	predicted protein	*Ostreococcus lucimarinus* CCE9901	green alga
189	303273364	adp-glucose pyrophosphorylase	*Micromonas pusilla* CCMP1545	green alga
191	255070935	adp-glucose pyrophosphorylase	*Micromonas* sp. RCC299	green alga
193	159470605	ADP-glucose pyrophosphorylase large subunit	*Chlamydomonas reinhardtii*	green alga
195	308814250	AGPLU2 (ISS)	*Ostreococcus tauri*	green alga
198	302840808	hypothetical protein VOLCADRAFT_75183	*Volvox carteri* f. nagariensis	green alga
199	145356323	predicted protein	*Ostreococcus lucimarinus* CCE9901	green alga
